# High-Affinity and Proteolytically Stable Peptidic Fluorescent NTS_1_R Ligands

**DOI:** 10.1021/acs.jmedchem.5c01701

**Published:** 2025-09-03

**Authors:** Fabian J. Ertl, Anna Friedel, Elena J. Schmid, Carina Höring, Nataliya Archipowa, Pierre Koch, Simone Maschauer, Roger J. Kutta, Olaf Prante, Max Keller

**Affiliations:** † Institute of Pharmacy, Faculty of Chemistry and Pharmacy, 9147 University of Regensburg, Universitätsstraße 31, Regensburg D-93053, Germany; ‡ Department of Nuclear Medicine, Molecular Imaging and Radiochemistry, 9171 Friedrich-Alexander-Universität Erlangen-Nürnberg (FAU), Kussmaulallee 12, Erlangen D-91054, Germany; § Institute of Biophysics and Physical Biochemistry, Faculty of Biology and Preclinical Medicine, 9147 University of Regensburg, Universitätsstraße 31, Regensburg D-93053, Germany; ∥ Institute of Physical and Theoretical Chemistry, Faculty of Chemistry and Pharmacy, University of Regensburg, Universitätsstraße 31, Regensburg D-93053, Germany; ⊥ Bavarian Cancer Research Center (BZKF), Translational Research Group TRAFO, Kussmaulallee 12, D-91054 Erlangen, Germany; # FAU NeW - Research Center New Bioactive Compounds, Friedrich-Alexander-Universität Erlangen-Nürnberg (FAU), Nikolaus-Fiebiger-Str. 10, Erlangen D-91058, Germany; 7 Bavarian Cancer Research Center (BZKF), Translational Research Group TRAFO, Franz-Josef-Strauß-Allee 11, Regensburg D-93053, Germany

## Abstract

Labeled ligands for the neurotensin receptor 1 (NTS_1_R), which is expressed in the CNS, the gastrointestinal tract, and in malignant tumors, are needed to investigate NTS_1_R-ligand binding and NTS_1_R expression. Aiming for fluorescence-labeled neurotensin(8–13)-derived NTS_1_R ligands with high affinity and proteolytic stability, several previous approaches were combined: (1) replacement of Arg^8^ by an amino-functionalized carbamoylated arginine, allowing conjugation to a fluorophore, (2) *N* ^α^-methylation of Arg^8^ and replacement of Tyr by β,β-dimethyl-l-Tyr^11^, conferring proteolytic stability, and (3) replacement of Leu^13^ by trimethylsilyl-Ala, boosting binding affinity. This strategy gave fluorescent NTS_1_R ligands with unprecedented NTS_1_R binding affinity (5-TAMRA-labeled ligand **19**: *K*_i_ 0.14 nM, sulfo-Cy5-labeled probe **21**: *K*_i_ 0.094 nM) and high stability in human plasma (*t*_1/2_ ≫ 48 h). Their suitability for competition binding studies (flow cytometry; **19**, **21**) and the imaging of NTS_1_R expression in living cells (confocal microscopy, biomolecular imaging; **19**, **21**) and tumor tissue (biomolecular imaging; **21**) is demonstrated.

## Graphical abstract

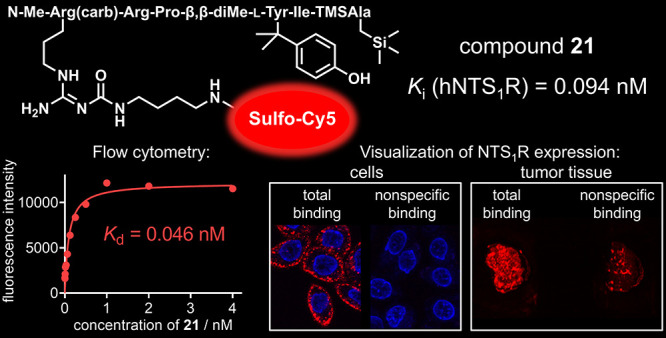

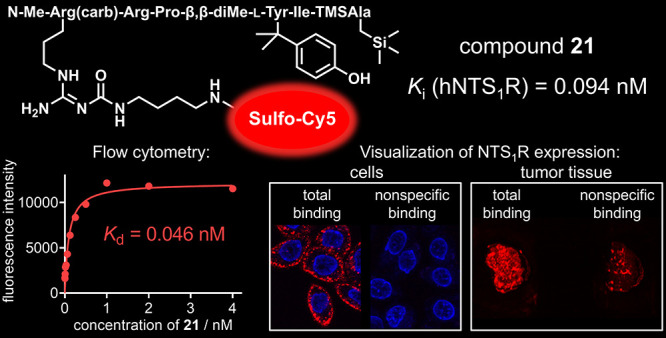

## Introduction

The neurotensin receptor 1 (NTS_1_R), a class A G-protein coupled receptor (GPCR), is one of three receptors of the neurotensin receptor family. The hexapeptide neuromedin N and the tridecapeptide neurotensin (NT) represent endogenous NTS_1_R agonists (Figure 1A). The bioactivity of neurotensin is conferred by the C-terminal hexapeptide sequence, NT(8–13).^1^ Thus, NT(8–13) has served as a lead structure for the development of various NTS_1_R ligands including radio- and fluorescence labeled analogs. In healthy tissues, the NTS_1_R is mainly expressed in the brain and gastrointestinal tract.^2,3^ With regard to neoplastic diseases, the NTS_1_R is (over)­expressed, e.g., in pancreatic adenocarcinoma, prostate and colon carcinoma, and in about one-third of primary breast cancer tumors.^4–9^ Therefore, the NTS_1_R is considered a promising target for tumor diagnostics and treatment using metabolically stable, appropriately radiolabeled NTS_1_R ligands.^10^

**Figure 1:**
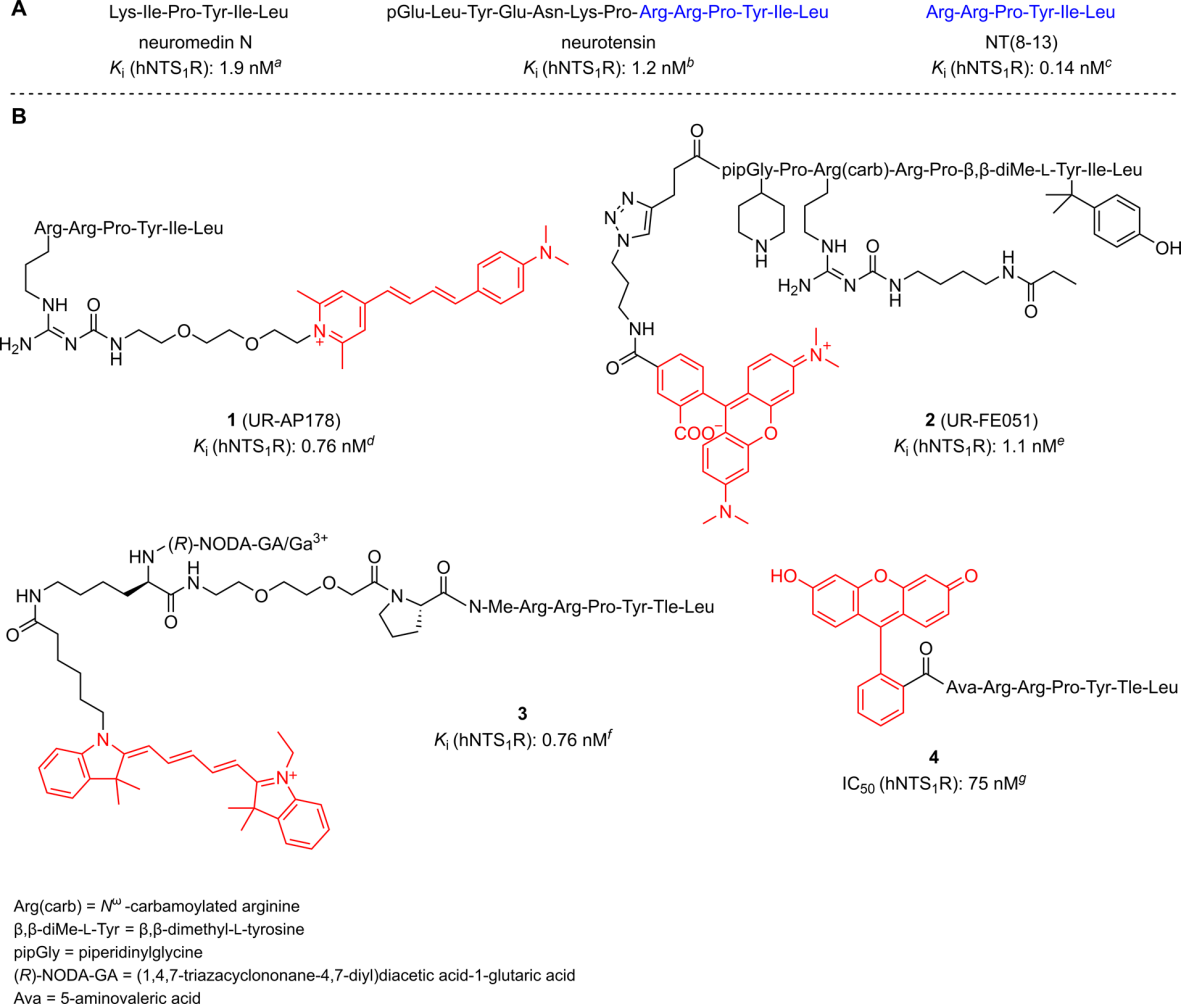
(A) Peptide sequences and NTS_1_R binding affinities of the endogenous NTS_1_R ligands neuromedin N and neurotensin, and the lead compound NT(8–13). (B) Reported fluorescent NTS_1_R ligands with binding affinities in the low nanomolar range. Fluorescent dyes are shown in red. ^*a*^Skrzydelski et al.,^17^ ^*b*^Granier et al.,^18^ ^*c*^Keller et al.,^12^ ^*d*^Keller et al.,^13^ ^*e*^Ertl et al.,^16^ ^*f*^Renard et al.,^14^ ^*g*^Maes et al.^11^

Besides radioligands, fluorescently labeled NTS_1_R ligands also represent useful tool compounds which can substitute radioligands, e.g., in competition binding assays needed to determine binding affinities of new NTS_1_R ligands. Advantages of fluorescent probes over radioligands are mild safety issues, unproblematic waste disposal, and accessibility to a broad range of methods such as flow cytometry, high-content imaging, fluorescence anisotropy or NanoBRET, most of them being compatible with multimode plate reader measurements. Moreover, fluorescent probes can be used to study receptor–ligand binding by fluorescence microscopy. Unlike radiochemical assays, most fluorescence-based techniques allow for measurement under homogeneous conditions, meaning that a separation of bound from free ligand is not required.

To date, several fluorescent NTS_1_R ligands, all derived from NT(8–13), have been reported (e.g., **1**–**4**, Figure 1B). These probes were characterized by high-content imaging, flow cytometry, fluorescence anisotropy, NanoBRET or fluorescence microscopy.^11–16^ Among the reported fluorescently labeled NTS_1_R ligands, compounds **1**–**3** exhibit the highest binding affinities (*K*_i_ = 0.76–1.1 nM) allowing an application as probes for cell-based binding assays.

Yet, there is interest in fluorescently labeled NTS_1_R ligands with even higher binding affinity since this comes along with reduced nonspecific binding and potentially with longer residence time, which is particularly advantageous for fluorescence-based imaging of receptor expression, e.g., in tumor tissue.

The design of the novel fluorescently labeled NT(8–13)-derivatives in this study was guided by different reported studies. First, Leu^13^ is replaced by (trimethylsilyl)­alanine (TMSAla) in the synthesized NT(8–13) analogs because Fanelli and coworkers reported that this structural modification leads to markedly increased NTS_1_R binding affinity of hexapeptides derived from NT(8–13) (see compounds **5** and **6**, Figure 2).^19^

**Figure 2:**
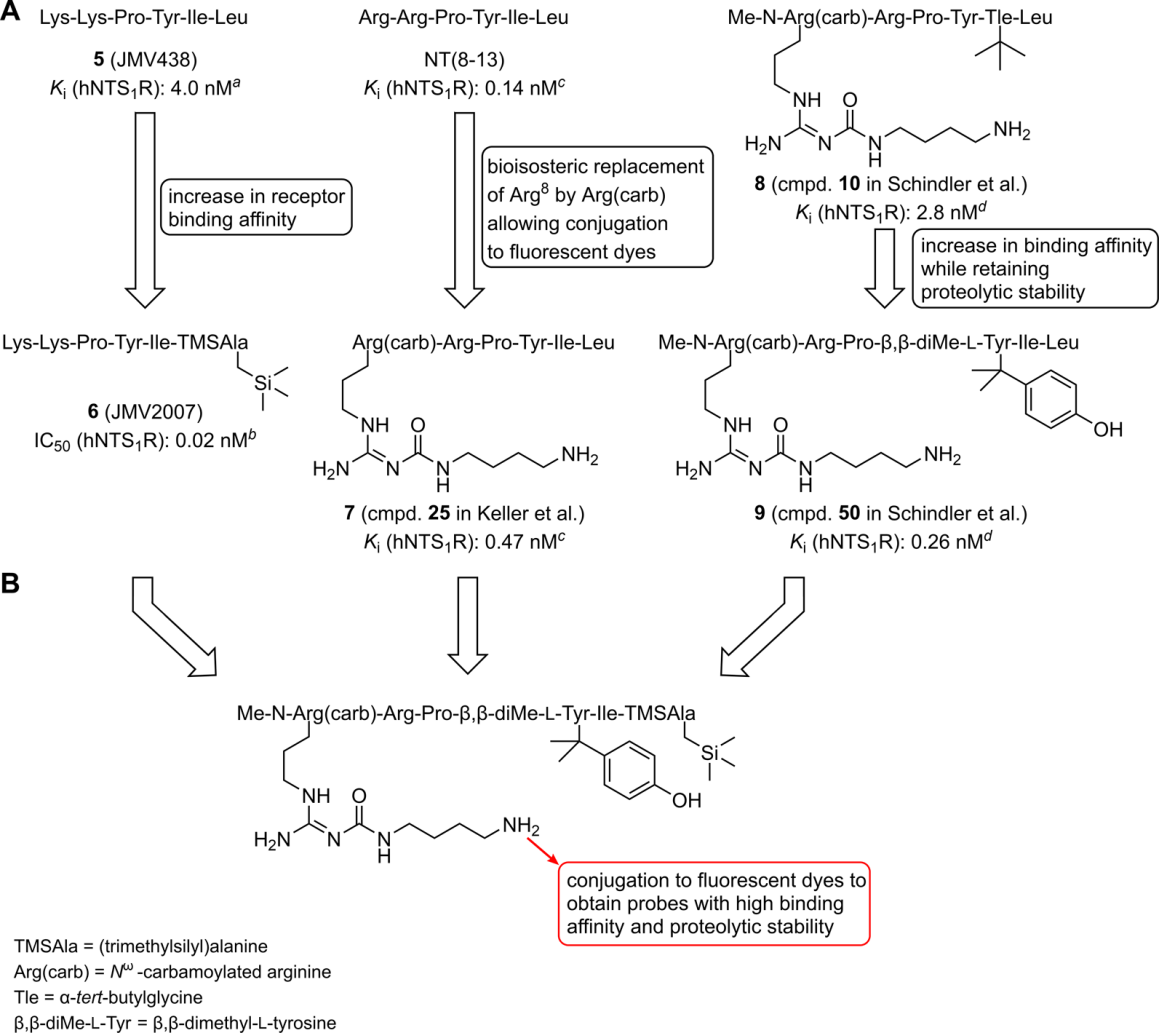
(A) Schematic presentation of reported amino acid replacements in NT(8–13) or NT(8–13) derivatives useful to increase NTS_1_R binding affinity (replacement of Leu^13^ by TMSAla), for peptide conjugation (replacement of Arg^8^ by an amino-functionalized carbamoylated arginine), and to achieve proteolytic stability (methylation of the N-terminus and replacement of Tyr^11^ by β,β-dimethyl-l-Tyr). (B) Aim of the present study: combination of approaches shown in A in one molecule followed by the synthesis of fluorescently labeled NTS_1_R ligands. ^*a*^Vivancos et al.,^20^ *^b^* Fanelli et al.,^19^ *^c^* Keller et al.,^12^ *^d^* Schindler et al.^21^

Second, Arg^8^ is bioisosterically exchanged by an amino-functionalized *N* ^ω^-carbamoylated arginine (Arg­(carb)) (e.g., compound **7**, Figure 2).^12^ Attachment of fluorescent dyes to the side chain of this carbamoylated arginine was previously demonstrated to be well tolerated with respect to NTS_1_R binding.^13,16^ Third, the N-terminus is methylated and Tyr^11^ is replaced by β,β-dimethyltyrosine, in order to obtain proteolytically stable peptides. The N-terminal methylation was reported to prevent N-terminal degradation by proteases^22^ and the incorporation of β,β-dimethyl-Tyr was recently shown to result in an effective stabilization of the C-terminus against proteolytic cleavage (see compounds **8** and **9**, Figure 2).^21^ Notably, the replacement of Tyr^11^ by β,β-dimethyl-Tyr represents a favorable alternative to the replacement of Ile^12^ by α-*tert*-butylglycine (standard modification for the stabilization of the C-terminus), since NTS_1_R binding is affected by the latter but not by the former modification.^21^

For fluorescence labeling of the peptides, the rhodamine-based dye 5-TAMRA and the indolinium-type cyanine dye sulfo-Cy5 were used. The precursor compounds and the labeled peptides were characterized in a radiochemical NTS_1_R competition binding assay. The labeled probes were also studied in a functional NTS_1_R Ca^2+^ assay and were photophysically characterized (fluorescent quantum yields and fluorescence lifetimes). Moreover, NTS_1_R binding of the fluorescent ligands was investigated by flow cytometry (saturation binding, kinetic studies, and competition binding), confocal microscopy, and in tumor sections using a biomolecular imager.

## Results and Discussion

### Synthesis

The unnatural, Fmoc-protected amino acids (**10**–**13**) used for the peptide synthesis are shown in Scheme 1A. *N* ^α^-methylated Fmoc/Pbf-protected arginine (**11**) and racemic Fmoc/*t*Bu-protected β,β-dimethyl-Tyr (**12**) were commercially available (note: the desired l-configured stereoisomer of **12** was not available as a pure enantiomer). The Fmoc/Boc-protected amino-functionalized, carbamoylated arginine (**13**) was prepared as reported.^12^ The Fmoc-protected TMSAla (**10**) was synthesized according to a published procedure yielding (*R*)-**10** with a reported ee of 98%.^23^ Here, this asymmetric synthesis, using (1*R*,2*R*,5*R*)-2-hydroxy-3-pinanone (**23**) as chiral auxiliary (see Scheme S1), gave (*R*)-**10** with an ee of 86% (determined with capillary electrophoresis, electropherograms shown in the Supporting Information).

**Scheme 1:**
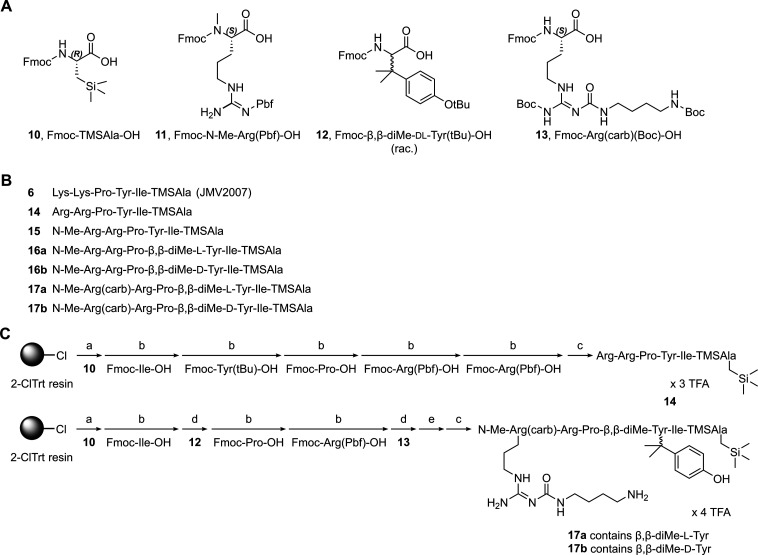
Structures of the Unnatural Amino acids **10**–**13**, Sequences of the Synthesized Peptides **6** and **14**–**17b**, and Exemplary Schematic Presentation of the Syntheses of **14** and **17** ^a^

a (A) Structures of used unnatural amino acids. (B) Amino acid sequences of the synthesized NT(8–13) derivatives. Overall yields for SPPS: 13–52%. (C) SPPS shown for **14** and **17**. Reagents and conditions: (a) anchoring of **10** to a 2-ClTrt resin: **10**/DIPEA (1/2.5 equiv), CH_2_Cl_2_, rt, overnight, Fmoc deprotection: 20% piperidine in DMF/NMP 8:2 v/v, rt, 2 × 10 min; (b) amino acid coupling: Fmoc-amino acid/HOBt/HBTU/DIPEA (5/5/4.9/10 equiv), DMF/NMP 8:2 v/v, 35 °C, 2 × 45 min (“double” coupling), Fmoc deprotection: as under (a); (c) (1) TFA/CH_2_Cl_2_ 1:3 v/v, rt, 2 × 20 min; (2) TFA/H_2_O 95:5 v/v, rt, 5 h; (d) **12** or **13**/HOBt/HBTU/DIPEA (3/3/2.95/6 equiv), DMF/NMP 8:2 v/v, 35 °C, 16 h (“single” coupling), Fmoc deprotection: as under (a); (e) N-terminal methylation according to reported procedures:^21,24^ (1) 2-nitrobenzenesulfonylchloride/collidine (3/5 equiv), CH_2_Cl_2_, rt, 2 h; (2) MTBD/methyl-4-nitrobenzenesulfonate (4/5 equiv), DMF, rt, 30 min; (3) DBU/2-mercaptoethanol (5/10 equiv), DMF, rt, 30 min. The separation of the diastereomers **17a** and **17b** was performed by achiral preparative RP-HPLC. The wavy bond in **17a/b** indicates either configuration, l or d.

All peptides (**6**, **14**–**17b**; sequences shown in Scheme 1B) were synthesized by solid-phase peptide synthesis (SPPS) on a 2-ClTrt resin. Peptide **14** represents the NT(8–13) derivative containing TMSAla instead of Leu^13^. To our best knowledge, this peptide has not been described to date. Peptide **15** represents the N-terminally methylated congener of **14** and in **16** Tyr^11^ is additionally replaced by β,β-dimethyl-Tyr (**16a**: β,β-dimethyl-l-Tyr, **16b**: β,β-dimethyl-d-Tyr). Finally, **17**, representing the precursor for fluorescence labeling, contains the *N* ^α^-methylated, amino-functionalized *N* ^ω^-carbamoylated arginine in position 8, β,β-dimethyl-l-Tyr (**17a**) or β,β-dimethyl-d-Tyr (**17b**) in position 11, and TMSAla in position 13 (note: the amino acid positions in the synthesized hexapeptides were assigned with the numbers corresponding to the respective amino acid positions in NT(8–13)). For **14** and **17**, the solid-phase synthesis is exemplarily illustrated in Scheme 1C. Regarding the separation of the diastereomers **16a** and **16b** as well as **17a** and **17b**, this could be conveniently achieved by achiral preparative RP-HPLC. The assignment of the absolute configuration to β,β-dimethyl-Tyr in **16a**–**17b** was carried out as recently reported for structurally related NT(8–13) derivatives.^16^ This approach is based on NT(8–13) analogs also containing β,β-dimethyl-l-Tyr or β,β-dimethyl-d-Tyr in position 11, for which the configuration was assigned using CD spectroscopy (structures shown in Figure S1A).^21^ As described by Ertl et al.,^16^ the elution order in RP-HPLC and the NTS_1_R binding affinities served as criteria for the assignment. Derivatives containing β,β-dimethyl-l-Tyr consistently elute first (RP-HPLC) and show markedly higher NTS_1_R binding affinities compared to the analogs containing β,β-dimethyl-d-Tyr (see Figure S1).

The fluorescently labeled peptides **19** and **21** were obtained by treatment of the precursor **17a** with the succinimidyl ester of the 5-TAMRA dye (**18**) or sulfo-Cy5 dye (**20**) in the presence of a base (Scheme 2). The fluorophores were chosen based on different criteria. Both dyes are commercially available at low to moderate costs and filter sets matching the excitation and emission wavelengths of these dyes are available for most instruments used for fluorescence detection. A favorable feature of the 5-TAMRA dye is its high photostability,^25^ whereas the sulfo-Cy5 dye is excitable at higher wavelengths (>600 nm) which is advantageous with respect to an application at cells and tissues. Moreover, both dyes contain a negatively charged acid function conveying increased polarity and solubility.

**Scheme 2:**
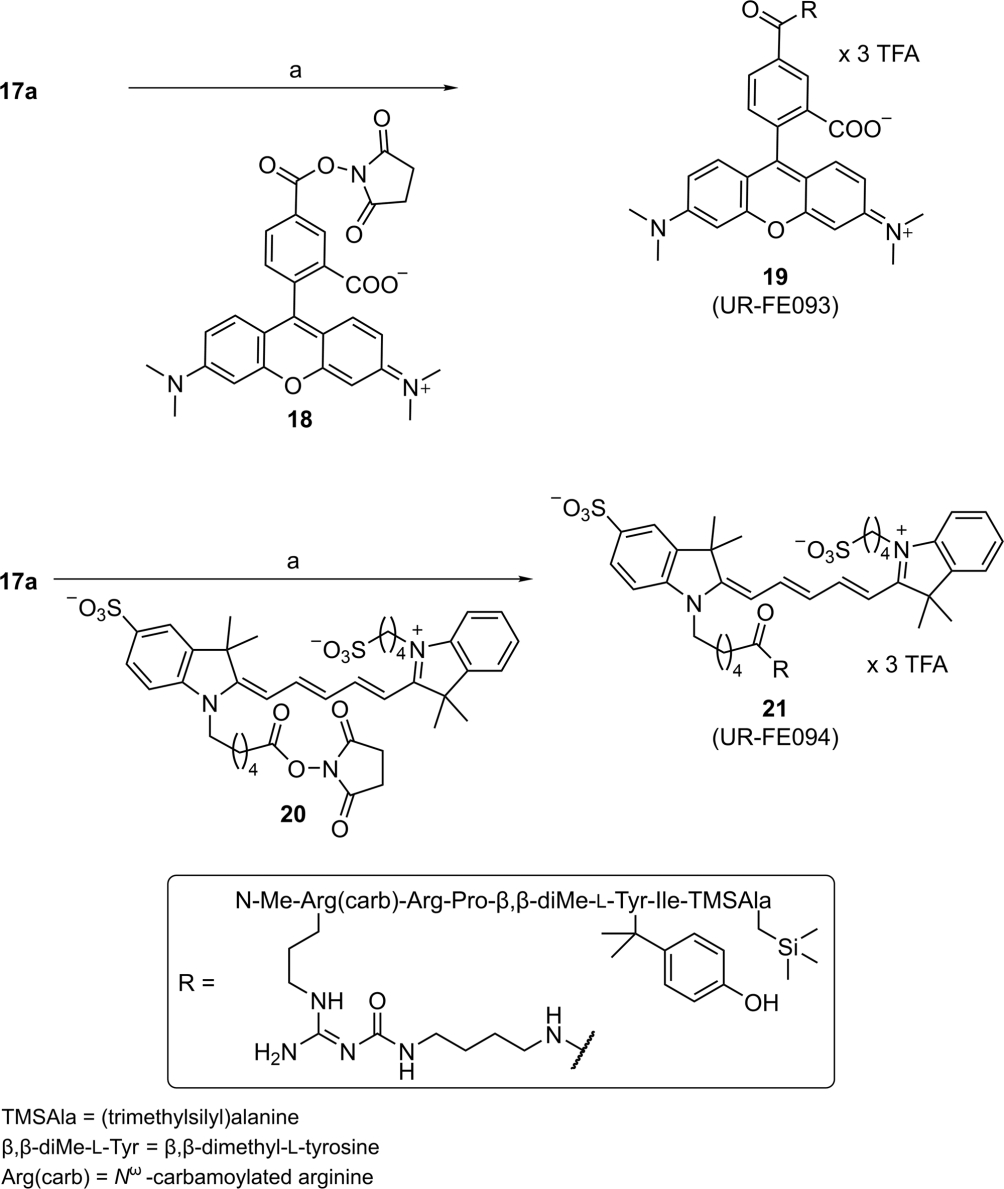
Synthesis of the Fluorescently Labeled Peptides **19** and **21** ^a^

a Reagents and conditions: (a) DIPEA, DMF/NMP 8:2 v/v, rt, 1 h; 40% (**19**), 30% (**21**).

### Chemical and Proteolytic Stability

The chemical stability of the fluorescently labeled ligands **19** and **21** was studied in phosphate-buffered saline (PBS, pH = 7.4) at room temperature over 48 h. Both compounds showed excellent stability (Figures S2 and S3). In the case of **21**, adsorption of the fluorescent ligand to the vessel (visible by eye) resulted in a moderate decrease (ca. 33%) in peptide concentration over 48 h (cf. Figure S3). The adsorption as the cause for the decrease in concentration is also supported by the fact that no additional peak was observed in the RP-HPLC chromatograms. The proteolytic stability of **15**, **17a**, **19**, and **21** was investigated in human plasma at 37 °C over 48 h. NT(8–13), studied as a control, showed very low plasma stability (complete degradation after 30 min, Table 1). The NT(8–13) analog **15**, being N-terminally methylated and containing TMSAla instead of Leu^13^ (cf. Scheme 1B), showed moderate proteolytic stability (ca. 50% intact peptide after 48 h). In contrast, the labeling precursor **17a** and the fluorescent ligands **19** and **21**, containing β,β-dimethyl-l-Tyr in position 11, exhibited high proteolytic stability (Table 1). The apparent low degradation of peptide **21** (10% after 48 h) can be attributed to the adsorption of **21** to the vessel material as also observed in the case of stability studies in PBS (discussed above). These results show that replacement of Leu^13^ by TMSAla as the only C-terminal modification in the NT(8–13) sequence is not sufficient to achieve high proteolytic stability. Consequently, the results confirm that N-terminal methylation in combination with the incorporation of β,β-dimethyl-Tyr in position 11 is highly effective in terms of proteolytic stability of NT(8–13) derivatives.

**Table 1:** Stabilities of NT(8–13), **15**, **17a**, **19**, and **21** in Human Plasma/PBS 1:2 v/v (37 °C)

	% intact compound in plasma after the specified incubation times^a^					
compd.	10 min	30 min	2 h	6 h	24 h	48 h
NT(8–13)	17 ± 1	<1	<1	<1	n.d.	n.d.
**15**	n.d.	n.d.	83 ± 1	80 ± 1	65 ± 1	47 ± 1
**17a**	n.d.	n.d.	>99	>99	>99	>99
**19**	n.d.	n.d.	>99	95 ± 2	93 ± 2	97 ± 1
**21**	n.d.	n.d.	97 ± 1	93 ± 1	90 ± 1	90 ± 2

a The initial concentration of the peptide in human plasma/PBS (1:2 v/v) was 80 μM. Data represent mean values ± SEM from two or three independent experiments (SEM not given when no degradation was observed).

### Neurotensin Receptor Binding Studied in Radiochemical Competition Binding Assays

NTS_1_R binding affinities were determined in a radioligand competition binding assay as reported^12^ using intact HT-29 colon carcinoma cells which express NTS_1_R endogenously but no NTS_2_R.^12^ The tritium-labeled NT(8–13) analog [^3^H]­UR-MK300 (structure shown in Figure S4) was used as radioligand. The obtained *K*_i_ values of **6**, **14**-**17b**, **19**, and **21** are presented in Table 2 and competition binding curves are shown in Figure S5A. The previously described peptide **6**, containing two lysines, exhibited high NTS_1_R binding affinity with a *K*_i_ value of 0.055 nM (Table 2) confirming the reported high binding affinity (IC_50_ = 0.02 nM^19^). Peptide **14**, containing TMSAla instead of Leu^13^ as the only structural difference compared to NT(8–13), displayed similar binding affinity as **6**. This confirmed the former observation that replacement of Arg^8^ and Arg^9^ by Lys^8^ and Lys^9^ does not affect NTS_1_R binding.^20,26–29^ N-methylation of **14** (peptide **15**) had no impact on NTS_1_R binding as shown in Table 2. This finding is consistent with a previously reported *N* ^α^-methyl scan of NT(8–13).^22^ Replacement of Tyr^11^ by β,β-dimethyl-l-Tyr in **15**, resulting in **16a**, did also not affect binding to the NTS_1_R (*K*_i_ of **16a**: 0.050 nM). Likewise, replacement of Arg^8^ in **16a** by the amino-functionalized *N* ^ω^-carbamoylated arginine (peptide **17a**) did not influence the NTS_1_R binding affinity (*K*_i_ of **17a**: 0.055 nM). As stated before, the peptides containing β,β-dimethyl-d-Tyr (**16b** and **17b**) showed markedly lower binding affinities (Table 2). The fluorescently labeled peptides **19** and **21**, bearing a bulky fluorescent dye at the side chain of Arg^8^, exhibited only slightly higher *K*_i_ values (*K*_i_ = 0.14 and 0.094 nM, respectively) compared to the precursor **17a**. This confirms that conjugation of NT(8–13) derivatives *via* an *N* ^ω^-carbamoylated arginine, incorporated in position 8, is a favorable approach in terms of preserving high NTS_1_R binding affinity. As a consequence, **19** and **21** bind to the NTS_1_R with similar binding affinity to NT(8–13) (*K*_i_ = 0.093 nM).

**Table 2:** NTS_1_R Binding Affinities of NT(8–13), **6**, **14-17b**, **19**, and **21**, and NTS_1_R Agonistic Potencies and NTS_2_R Binding Affinities of **19** and **21**

	NTS_1_R		NTS_2_R
compd.	p*K*_i_ ± SEM/*K*_i_ [nM]^a^	pEC_50_ ± SEM/EC_50_ [nM]^b^	p*K*_i_ ± SEM/*K*_i_ [nM]^c^
NT(8–13)	10.03 ± 0.03/0.093	8.96 ± 0.07/1.2	9.23 ^16^/0.62 ^16^
**6**	10.28 ± 0.06/0.055	n.d.	n.d.
**14**	10.10 ± 0.02/0.079	n.d.	n.d.
**15**	10.43 ± 0.1/0.041	n.d.	n.d.
**16a**	10.30 ± 0.05/0.050	n.d.	n.d.
**16b**	7.91 ± 0.06/12	n.d.	n.d.
**17a**	10.39 ± 0.2/0.055	n.d.	n.d.
**17b**	8.42 ± 0.1/4.2	n.d.	n.d.
**19**	9.86 ± 0.09/0.14	9.00 ± 0.09/1.1	9.11 ± 0.02/0.78
**21**	10.04 ± 0.06/0.094	8.89 ± 0.09/1.4	9.14 ± 0.07/0.75

a Determined by radioligand competition binding with [^3^H]­UR-MK300 ^12^ at HT-29 colon carcinoma cells.

b Determined in a Fura-2 Ca^2+^ assay at HT-29 cells.

c Determined by radioligand competition binding with [^3^H]­UR-MK300 ^12^ at membranes of HEK293T-hNTS_2_R cells. Data represent mean values ± SEM (p*K*_i_, pEC_50_) or mean values (*K*_i_, EC_50_) from at least three independent experiments performed in triplicate. n.d. not determined.

In addition to NTS_1_R binding, NTS_2_R binding affinities were determined for the fluorescent ligands **19** and **21** in a radiochemical competition binding assay using membranes of HEK293T-hNTS_2_R cells^21^ and [^3^H]­UR-MK300 as radioligand (competition binding curves see Figure S5B). With p*K*_i_ values of 9.11 and 9.14, respectively, both probes bind with slightly lower affinity to NTS_2_R than to NTS_1_R (Table 2).

### NTS_1_R Agonism

The agonistic potencies of NT(8–13) and the fluorescently labeled NTS_1_R ligands **19** and **21** were determined in a Fura-2 Ca^2+^ assay using HT-29 cells (concentration–response curves shown in Figure S6). Peptides **19** and **21** proved to be full agonists with potencies comparable to that of NT(8–13) (Table 2). The pEC_50_ values were consistently circa one log unit lower than the corresponding p*K*_i_ values, which can be explained by the nonequilibrium conditions in the case of the Fura-2 Ca^2+^ assay: as the signal occurs immediately after addition of the agonist, the receptor occupancy at the time of the readout is considerably lower than the hypothetical receptor occupancy that would be achieved in equilibrium for the respective concentrations of receptor and receptor ligand. An impairment of the optical readout of the assay by the 5-TAMRA label, whose absorption spectrum (λ_max_ = 555–558 nm, cf. Table 3) largely overlaps with the emission spectrum of the Fura-2 Ca^2+^ complex (λ_max_ = 505 nm),^30^ can be excluded based on the results of previous studies using a 5-TAMRA dummy ligand.^31^

**Table 3:** Maxima of the Absorption, Excitation, and Emission Spectra, Fluorescence Lifetimes and Fluorescence Quantum Yields of **19** and **21** in PBS in the Presence or Absence of BSA (1% w/w)

	λ_abs_/λ_ex_/λ_em_ [nm]|Δ[eV]^a^		τ_fl_ [ns]^b^		Φ_fl_ (%)	
compd.	PBS	PBS + 1% BSA	PBS	PBS + 1% BSA	PBS	PBS + 1% BSA
**19**	558/552/583|0.12	555/553/578|0.097	2.7	3.5	41	34
**21**	647/648/665|0.049	650/651/669|0.051	1.4	2.7	29	39

a Calculated based on λ_ex_ and λ_em_.

b Only the main component of a biexponential fit with a contribution >90% is presented.

### Photophysical Characterization

The absorption of the first electronic transition and the emission spectrum of both compounds are their corresponding mirror images indicating a rather constrained molecular scaffold both in the electronic ground and first excited state (Figure S7A,C). However, in the presence of BSA (1% w/w) the spectral distributions shift. In the case of **19**, the spectra blue-shift, while in the case of **21** the spectra red-shift. This already indicates an interaction with the protein’s surface. Further, while in the case of **21** the excitation spectrum always resembles the corresponding absorption spectrum with or without BSA, the excitation spectrum of **19** recorded in the absence of BSA already shows a blueshift compared to the corresponding absorption spectrum. This indicates a significant structural rearrangement of the molecular framework in the excited state compared to the ground state. The impact of BSA in form of interactions to the chromophore is further manifested in altered fluorescence quantum yields and lifetimes (Table 3). Each chromophoric system shows a prolonged excited singlet state lifetime in the presence of BSA. To note, the emission decay is biexponential and the second lifetime is of small amplitude (cf. Figure S7B,D), which may arise either from small impurities or an equilibrium between different conformers. Interestingly, while **19** shows a decreased fluorescence quantum yield, **21** shows an increased fluorescence quantum yield. In the case of **19**, this may indicate a deactivation process induced by the interaction with the protein. An electron transfer from a redox active amino acid to the chromophore or the hindrance for structural changes in the excited state, which could result in a brighter emissive configuration, may represent two potential explanations. This will be addressed by transient absorption spectroscopy and presented elsewhere. In the case of **21**, the emissive character of the electronically excited chromophore is apparently inversely effected. Here, the data suggest a situation in which, in the excited state, the molecule undergoes structural changes forming a less emissive configuration that is hindered when attached to the protein’s surface. Also, in this case the transient absorption will inform on the mechanistic scenario in more detail, which will be presented elsewhere. The observed changes in quantum yield are in accordance with reported studies on 5-TAMRA-labeled and sulfo-Cy5-labeled fluorescent GPCR ligands.^13,31–33^

### Investigation of NTS_1_R Binding of **19** and **21** by Flow Cytometry

The flow cytometric binding experiments were carried out at 23 °C using a BD FACSCanto II cytometer equipped with an argon laser (488 nm) and a red laser (640 nm). Saturation binding experiments with **19** and **21** were performed at live human HT-29 colon carcinoma cells and stably transfected CHO-hNTS_1_R cells. For both ligands, specific binding was saturable (Figures 3A and S8), however, the apparent *K*_d_ values determined at CHO-hNTS_1_R cells were higher (10–20-fold) compared to the apparent *K*_d_ values obtained from HT-29 cells (Table 4) which were in good agreement with the *K*_i_ values determined by competition binding with [^3^H]­UR-MK300 at HT-29 cells (Table 2). The higher *K*_d_ values determined at CHO-hNTS_1_R cells can be explained by the markedly higher NTS_1_R expression in CHO-hNTS_1_R cells (ca. 300,000 receptors/cell) compared to HT-29 cells (ca. 45,000 receptors/cell).^12^ As the studied ligands **19** and **21** show subnanomolar binding affinities, a high receptor concentration in saturation binding experiments leads to ligand depletion and consequently results in higher apparent *K*_d_ values.^34^ This effect can be reduced by using less cells in the experiment. Typically, for this kind of flow cytometric analysis, cell densities ≥100,000 cells/mL are used to obtain a reasonable number of gated events (≥2000), required for the calculation of representative fluorescence intensity mean values, within a reasonable measuring time. To explore the influence of the receptor concentration and associated with this the effect of ligand depletion on the discrepancy between the apparent *K*_d_ and the true *K*_d_, additional saturation binding experiments were performed with CHO-hNTS_1_R cells using a considerably lower cell density of 15,000 cells/mL. These conditions resulted in 200–300 gated events, just high enough to enable a statistically reasonable data analysis. The apparent *K*_d_ values of **19** and **21** obtained from these saturation binding experiments were markedly lower (factor > 10) than the apparent *K*_d_ values obtained from saturation binding studies using 150,000 cells/mL (Table 4 and Figure S8) and similar to the *K*_d_ values determined at HT-29 cells. The apparent *K*_d_ values determined at low receptor concentrations (HT-29 cells, CHO-hNTS_1_R cells at a density of 15,000 cells/mL) should better resemble the true *K*_d_, although the latter is potentially even lower than the experimentally determined dissociation constants.

**Figure 3:**
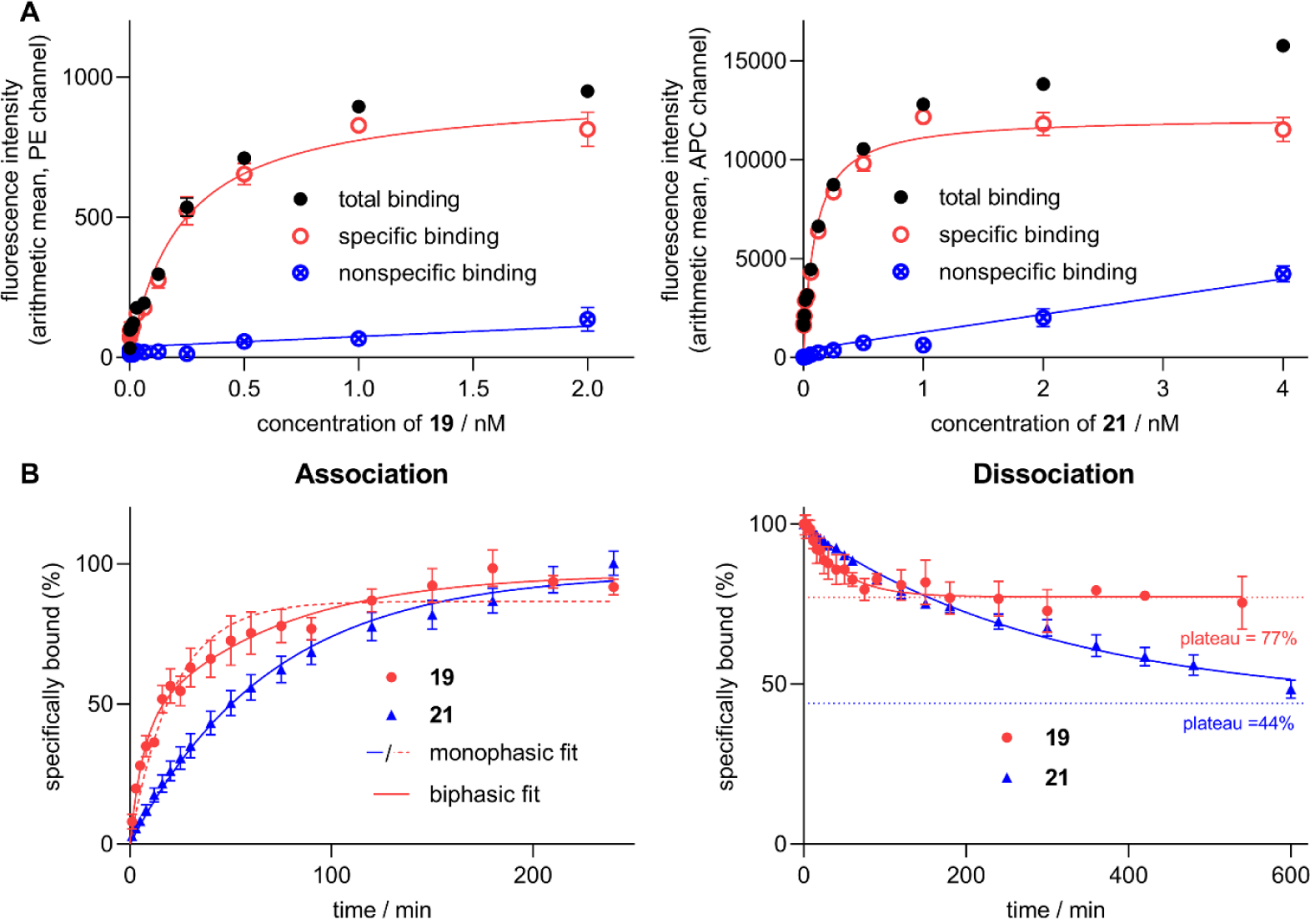
NTS_1_R equilibrium binding and kinetic studies of **19** and **21** determined by flow cytometry at intact HT-29 cells. (A) Representative binding isotherms (specific binding, open symbols) of **19** and **21** obtained from saturation binding experiments (incubation: 120 min at 23 °C). Nonspecific binding was determined in the presence of 1 μM NT(8–13). *K*_d_ values are presented in Table 4. Data represent mean values ± SEM (total and nonspecific binding) or calculated values ± propagated error (specific binding) from a representative experiment performed in triplicate. (B) Association and dissociation of **19** and **21** studied at 23 °C. Concentrations of **19** and **21** used for the association: 1 nM and 0.1 nM, respectively; concentrations of **19** and **21** used for the preincubation period (**19**: 120 min, **21**: 180 min) of dissociation experiments: 1 nM and 0.25 nM, respectively. Dissociation and association rate constants are presented in Table 4. Data represent mean values ± SEM from at least three independent experiments performed in triplicate.

**Table 4:** NTS_1_R Binding Data of **19** and **21** Determined by Flow Cytometry at HT-29 and CHO-hNTS_1_R Cells

	saturation binding		binding kinetics (HT-29 cells)			
	apparent *K*_d_ [nM]					
compd.	HT-29 cells	CHO-hNTS_1_R cells	*k*_obs_ [min^–1^]	*k*_on_ [nM^–1^min^–1^]^a^	*k*_off_ [min^–1^]	*K*_d_(kin) [nM]^b^
**19**	0.11 ± 0.04	1.2 ± 0.3^c^	*k*_obs,mono_ = 0.039 ± 0.005	*k*_on,mono_ = 0.017 ± 0.01	0.021 ± 0.007	1.2 ± 1.3
		0.034 ± 0.009^d^	*k*_obs(bi,fast)_ = 0.19 ± 0.03	*k*_on(bi,fast)_ = 0.16 ± 0.04		0.13 ± 0.07
		0.88 ± 0.1^c^	*k*_obs(bi,slow)_ = 0.016 ± 0.003	*k*_on(bi,slow)_ = −0.0055 ± 0.01		n.a.
**21**	0.046 ± 0.02	0.022 ± 0.005^d^	0.014 ± 0.002	0.11 ± 0.02	0.0033 ± 0.0002	0.030 ± 0.007

a Association rate constant ± propagated error calculated from *k*_off_, *k*_obs_, and the ligand concentration used for the association experiments.

b Kinetically derived dissociation constant ± propagated error calculated from *k*_off_ and *k*_on_ values. *K*_d_, *k*_obs_, and *k*_off_ values represent mean values ± SEM from at least three independent experiments; *k*_on_ and *K*_d_(kin) values represent calculated values ± propagated error.

c Experiments performed with a cell density of 150,000 cells/mL.

d Experiments performed with a cell density of 15,000 cells/mL. n.a. not applicable.

Noteworthy, the excitation laser line (640 nm) used for the excitation of the sulfo-Cy5 dye in **21** is close to its absorption maximum (647/650 nm, see Table 3). In contrast, the excitation of the 5-TAMRA label in **19** by the 488 nm argon laser is not ideal with respect to its absorption maximum (558/555 nm, Table 3). This is also reflected by the markedly lower *B*_max_ values obtained from saturation binding experiments with **19** compared to **21** (Figure 3A).

Kinetic binding studies, performed at HT-29 cells, revealed notable differences between **19** and **21**. While **21** showed a monophasic association, the 5-TAMRA-labeled ligand **19** displayed a clear biphasic association (two-phase association favored over one-phase association according to the *F*-test, *P* < 0.005, GraphPad Prism 5) (Figure 3B). The proportion of the fast and slow association phase was 42:58 (based on the fit to the data shown in Figure 3B). The observed association rate constants *k*_obs_ and the calculated association rate constants *k*_on_ are shown in Table 4. The *k*_on(bi,slow)_ value, calculated from *k*_obs(bi,slow)_, *k*_off_, and the concentration of **19** used for the association studies, was negative. This could be due to internalization of ligand–receptor complex during the association process (cf. Figure 6), but could also be attributed to the low ratios of total over nonspecific binding in the case of **19** particularly during the initial phase of the association (discussed in more detail below). As the *k*_on(bi,slow)_ was negative, precluding a calculation of the kinetically derived dissociation constant *K*_d_(kin), the association data of **19** were additionally fitted with a monophasic exponential fit covering the whole association process. This enabled the calculation of a *K*_d_(kin) value which comprises the entire association (see below).

The fact that one ligand shows a monophasic and the other ligand a biphasic association for the same cellular system suggests that the biphasic association cannot be explained by the existence of two subpopulations of NTS_1_R (e.g., one coupled to and the other uncoupled from G-protein). It should be mentioned that for the association studies of **19**, a fluorescent ligand concentration corresponding to 9-fold the *K*_d_ value of **19** had to be used since with lower ligand concentrations, correlating with lower receptor occupancies, the difference between total and nonspecific binding was too low for a reliable data analysis. This was particularly due to the aforementioned mismatch of the excitation wavelength and the absorption maximum of the 5-TAMRA dye in **19**. In contrast, for association studies with **21**, a ligand concentration corresponding to 3-fold the *K*_d_ value could be used because of the optimal excitation of the sulfo-Cy5 dye in **21** with the red laser (640 nm) resulting in the detection of high fluorescence intensities. It is noteworthy that, in the case of experiments with **19**, the excitation with 488 nm light gave a high autofluorescence of the cells which also affected data analysis. The excitation of the cells with 640 nm (used for **21**) resulted in very low autofluorescence. The different ligand concentrations used for the association experiments might be the reason for the observed differences in the association kinetics of **19** and **21** (biphasic vs monophasic). Moreover, it could be explained by the internalization of ligand–receptor complex (as confirmed by confocal microscopy, see below), occurring already during the association process. Upon internalization, ligand-dependent differences could occur with respect to changes of fluorescent properties in the cell interior and retrafficking of ligand–receptor complex or free receptor to the plasma membrane.

Dissociation studies at HT-29 cells, initiated by adding an excess of NT(8–13), gave monophasic dissociation curves revealing an incomplete dissociation (plateau significantly higher than zero, *P* < 0.05, *t*-test) as also previously reported for fluorescently labeled NT(8–13) derivatives.^13,16^ The plateau values amounted to 77% (**19**) and 44% (**21**). This can be explained by the NTS_1_R agonism of **19** and **21** (cf. Figure S6) meaning that the ligand–receptor complex is internalized, potentially followed by intracellular dissociation of the fluorescent ligand from the receptor. The *k*_off_ values of **19** and **21** differed by a factor of 7 (*k*_off_ = 0.021 min^–1^ vs 0.0033 min^–1^, see Table 4). Compared to previously described fluorescently labeled NT(8–13) derivatives, **19** and **21** show a slower dissociation from NTS_1_R which is favorable for imaging of NTS_1_R expression in cells or tissues. The kinetically derived dissociation constants *K*_d_(kin) (Table 4) were calculated according to *K*_d_(kin) = *k*_off_/*k*_on_. In the case of **19**, the *K*_d_(kin) was calculated from *k*_on,mono_ and *k*_on(bi,fast)_ yielding *K*_d_(kin) values of 1.2 and 0.13 nM, respectively. The value of 0.13 nM is in excellent agreement with the *K*_d_ obtained from saturation binding (Table 4). Likewise, the *K*_d_(kin) value obtained for **21** is in good agreement with the *K*_d_ from saturation binding studies (0.030 nM vs 0.046 nM), indicating a binding process largely following the law of mass action. Yet, the determined kinetic parameters of **19** and **21** must be interpreted with care since both ligands induce receptor endocytosis (cf. Figures 6 and 7) and show an incomplete dissociation. Moreover, as discussed before, flow cytometric kinetic studies with the 5-TAMRA-labeled ligand **19** were compromised by the nonoptimal excitation and higher autofluorescence compared to **21**.

To investigate the suitability of **19** and **21** to serve as probes for the determination of NTS_1_R binding affinities of unlabeled NTS_1_R ligands, the *K*_i_ values of reported NTS_1_R ligands (NT­(8-13), SR142948, SR48692, UR-MK300) were determined in flow cytometric competition binding studies at HT-29 cells using **19** or **21** as labeled probe (displacement curves shown in Figure 4).

**Figure 4:**
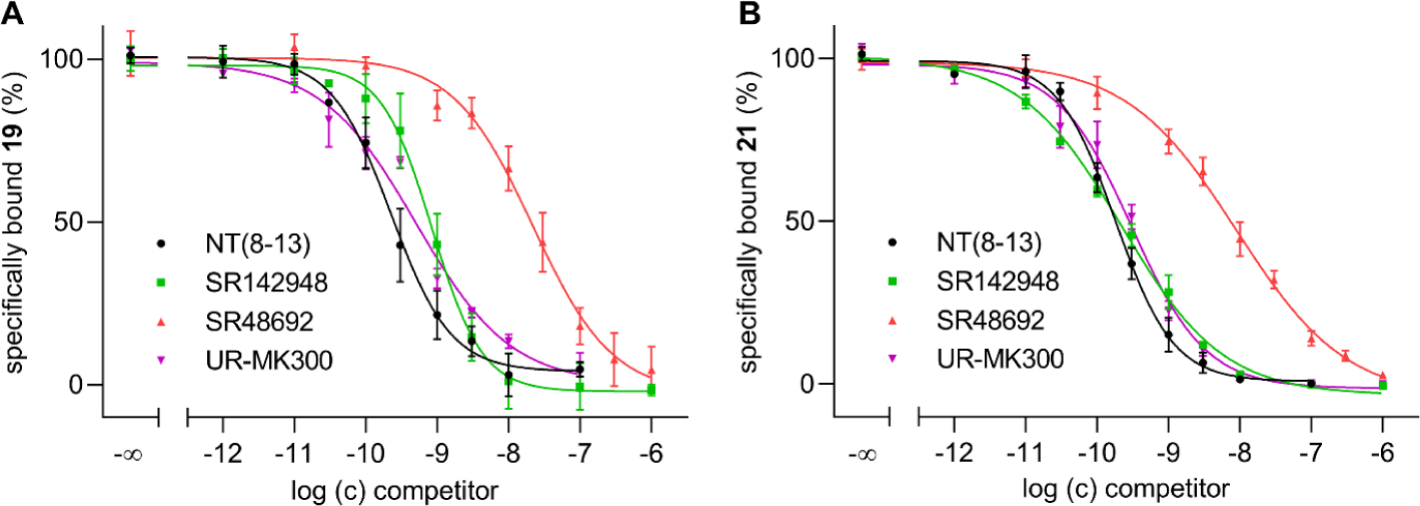
Displacement curves from flow cytometric competition binding studies using **19** (A) or **21** (B) as a labeled probe and NT(8–13), SR142948, SR48692, or UR-MK300 as competitors. Experiments were performed at intact HT-29 cells at 23 °C. The concentrations of **19** and **21** were 0.3 nM and 0.1 nM, respectively. Incubation times: 120 min (A) and 180 min (B). Data represent mean values ± SEM from at least three individual experiments performed in triplicate.

Although the dissociation kinetics of **19** and **21**, indicating long-lasting binding, is not ideal in terms of competition binding experiments, the p*K*_i_ values obtained for the reference ligands were generally in good agreement with reported data (Table 5).

**Table 5:** NTS_1_R Binding Affinities of Reported NTS_1_R Ligands Determined by Flow Cytometry at HT-29 Cells Using **19** and **21** as Labeled Probes^a^

	p*K*_i_		
compound	**19**	**21**	literature
NT(8–13)	10.20 ± 0.2	10.25 ± 0.09	9.85,^12^ 9.62,^28^ 9.54,^35^ 9.00,^18^ 9.55,^16^ 10.09 ^16^
SR142948	9.70 ± 0.1	10.14 ± 0.08	8.96,^12^ 9.24,^16^ 9.74,^16^ 9.00 ^36^
SR48692	8.25 ± 0.1	8.49 ± 0.2	8.07 ^37^
UR-MK300	9.85 ± 0.1	10.05 ± 0.1	9.33 ^12^

a Reported *K*_i_ values were converted to p*K*_i_ values. Data represent mean values ± SEM from at least three individual experiments performed in triplicate.

The Hill coefficients of the displacement curves (4-parameter logistic fit) were not significantly different from −1 (two-tailed *t* test, *P* > 0.05) except for SR142948 and SR48692 studied with **21** (Hill coefficients: −0.63 ± 0.02 and −0.55 ± 0.06, respectively; mean values ± SEM). The low slope factors might be attributed to the (pseudo)­irreversible binding of **21** (cf. Figure 3B) or to the internalization and putative externalization of NTS_1_R induced by the fluorescent agonists, potentially resulting in a retarded displacement of fluorescent ligand. However, for the other competitors (NT(8–13), MK300) and for all competitors studied with **19**, also showing (pseudo)­irreversible binding, the Hill coefficients were not significantly lower than unity, which contradicts the aforementioned hypothesis. Since slope factors different from unity could also arise from the existence of ligand-dependent multiple conformational receptor states,^38,39^ the low slopes observed for SR142948 and SR48692 could be caused by the preference of different receptor conformations of the used NTS_1_R ligands: whereas the agonist **21** prefers the active conformation, the antagonists do not discriminate between receptor states or even prefer the inactive conformation.^40,41^

### Cooperativity between **21** and the Allosteric NTS_1_R Modulator SBI-553

In recent years, several allosteric NTS_1_R modulators, targeting an intracellular binding site, have been reported.^42–45^ *In vivo* studies suggested that allosteric potentiators of NTS_1_R binding of neurotensin can potentially be used as therapeutics for the treatment of pain.^45^ A prominent example is the positive allosteric modulator SBI-553 which was demonstrated to potentiate binding of radiolabeled neurotensin or NT(8–13).^42,44^ To explore if SBI-553 also enhances binding of fluorescently labeled NT(8–13) derivatives such as **21**, the effect of SBI-553 on NTS_1_R binding of **21** was studied by flow cytometry using HT-29 tumor cells. For these experiments, **21** was used at a concentration of 0.01 nM, corresponding to approximately one-fifth of its *K*_d_, and these samples were titrated with SBI-553. As previously observed in the radiochemical assays,^42,44^ SBI-553 clearly enhanced NTS_1_R binding of **21** (Figure 5). However, in contrast to the reported studies, a biphasic CEC of SBI-553 was obtained, yielding a pEC_50_(high) of 10.37 and a pEC_50_(low) of 7.28. The latter is in excellent agreement with the reported pEC_50_ value of SBI-553 of 7.30 ^44^ and with the described EC_50_ value of 140 nM.^42^ To note, in contrast to the pEC_50_(low), the pEC_50_(high) was not well reproducible in the individual experiments which is reflected by the high SEM of 0.40 (cf. Figure 5). The *E*_max_ observed for SBI-553 (*E*_max_ = 88%) was lower than the reported *E*_max_ of 140%^42^ and 242%.^44^

**Figure 5:**
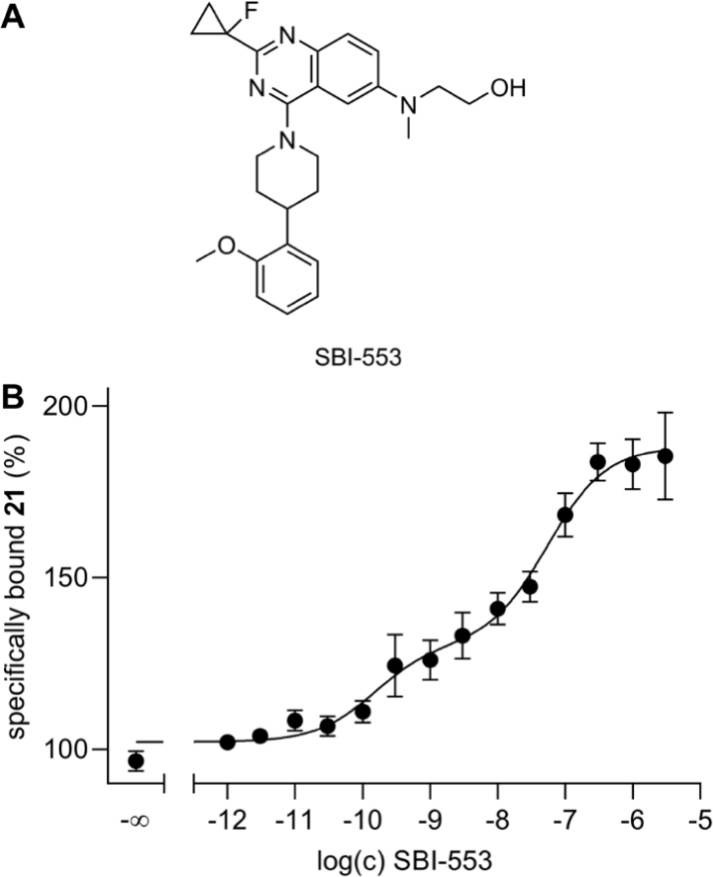
(A) Structure of the allosteric NTS_1_R modulator SBI-553. (B) Effect of SBI-553 on NTS_1_R binding of the fluorescent NT(8–13) derivative **21** (*c* = 0.01 nM) determined in a flow cytometric binding assay performed with intact HT-29 cells at 23 °C. Cells were preincubated with SBI-553 for 30 min prior to the addition of **21** and continued incubation for 180 min. SBI-553 enhanced binding of **21** in a biphasic manner (fraction high/low: 0.33/0.67). 100% specifically bound **21** corresponds to specific binding of **21** in the absence of SBI-553. Data represent mean values ± SEM from six individual experiments performed in triplicate. pEC_50_(high) = 10.37 ± 0.40. pEC_50_(low) = 7.28 ± 0.16. *E*_max_ = 88 ± 8% (difference between lower and upper plateau).

It is a matter of speculation why SBI-553 shows a biphasic CEC when studied with the fluorescent ligand **21**. The major differences compared to the reported radiochemical assays^42,44^ are the type of labeled ligand (radiolabeled vs fluorescently labeled), the use of intact cells (in contrast to membrane preparations in the reported studies), and a measurement under equilibrium conditions in the present study (flow cytometry). Supposedly, the biphasic course of the CEC of SBI-553 can be attributed to one or more of these differences in the technical setup of the binding assays. The elucidation of the underlying mechanisms requires further studies. Likewise, the lower *E*_max_ compared to the reported *E*_max_ values^42,44^ could also arise from the different assay parameters.

### Confocal Microscopy

Binding of **19** and **21** to both HT-29 cells, natively expressing NTS_1_R, and stably transfected CHO-hNTS_1_R cells, overexpressing the NTS_1_R, was studied by confocal microscopy at 22 °C using a confocal laser scanning microscope. In all cases, a marked difference between total and nonspecific binding was found (Figures 6 and 7; Figures S9 and S10), showing that the NTS_1_R expression can be visualized with both fluorescent ligands, even at low receptor expression levels (HT-29 cells, Figures 6 and 7).

**Figure 6:**
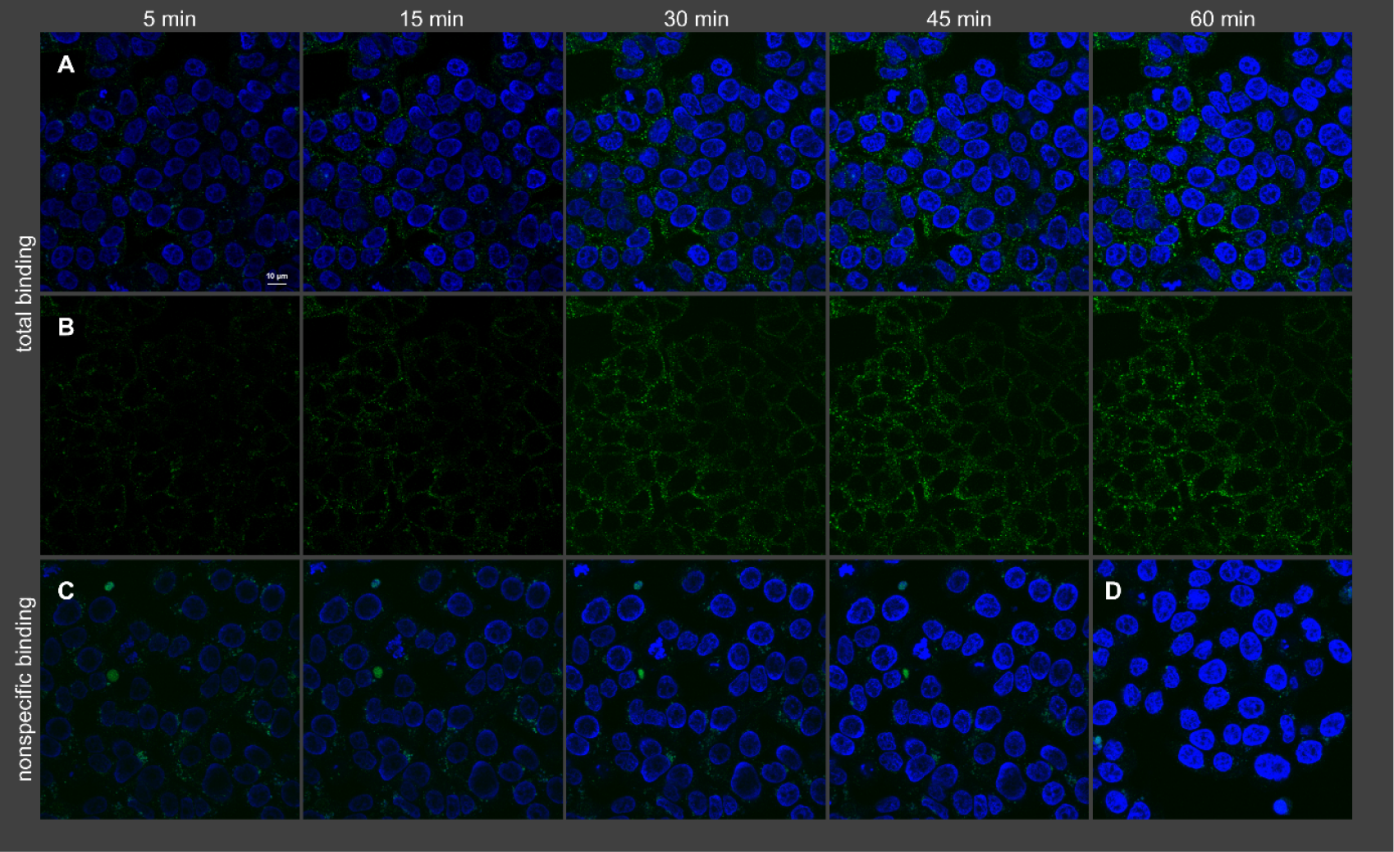
Visualization of binding of **19** (2 nM) to intact HT-29 cells (temperature: 22 °C) by confocal microscopy. Shown is total binding (A, B), nonspecific binding (C), and autofluorescence (D). Nuclei were stained with H33342 (2 μg/mL). (A) Merged fluorescence of **19** (green) and nuclei (blue). (B) Fluorescence of **19**, without nuclei. (C) Merged fluorescence of **19** and nuclei acquired in the presence of 1 μM NT(8–13).

**Figure 7:**
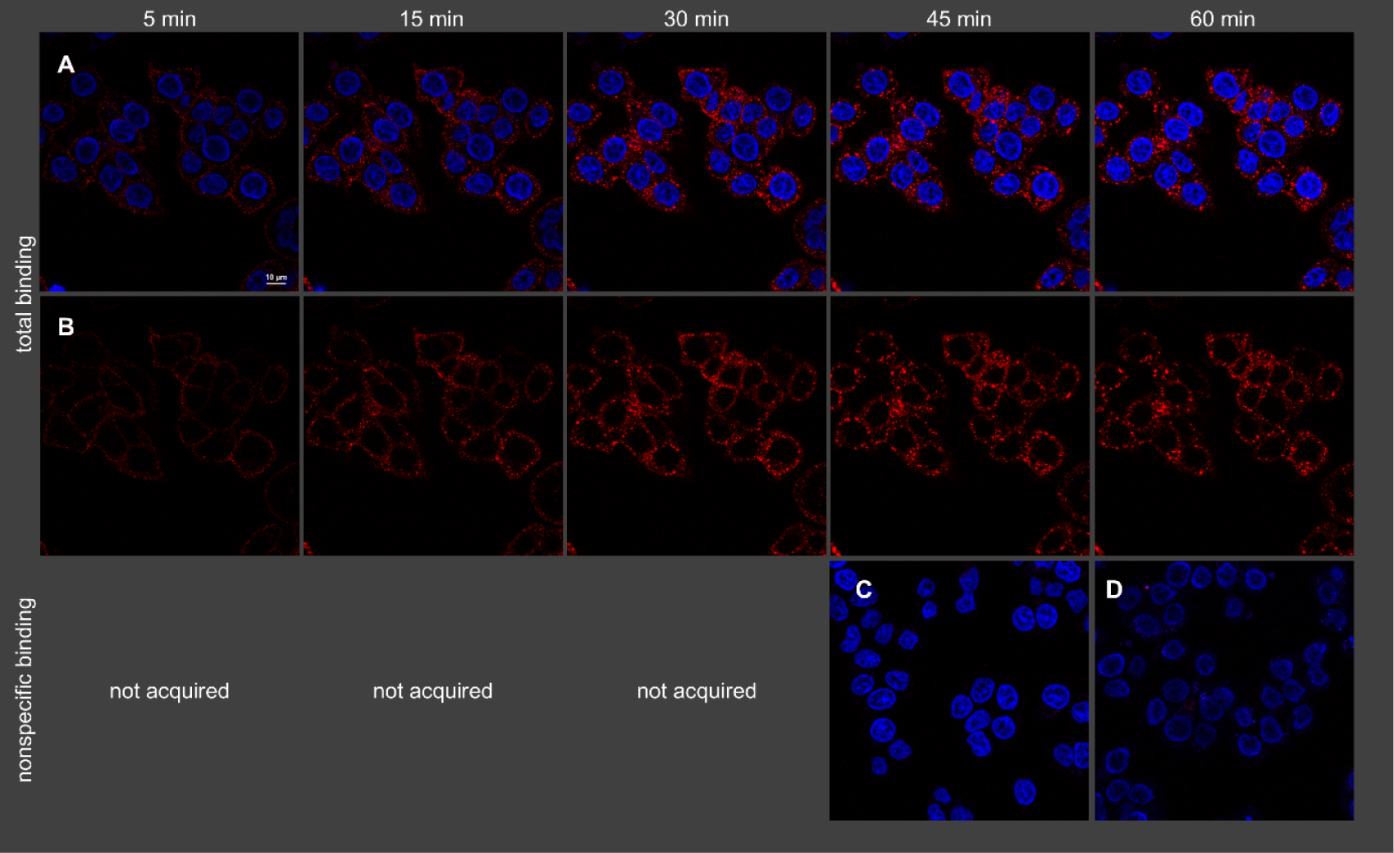
Visualization of binding of **21** (2 nM) to intact HT-29 cells (temperature: 22 °C) by confocal microscopy. Shown is total binding (A, B), nonspecific binding (C), and autofluorescence (D). Nuclei were stained with H33342 (2 μg/mL). (A) Merged fluorescence of **21** (red) and nuclei (blue). (B) Fluorescence of **21**, without nuclei. (C) Merged fluorescence of **21** and nuclei acquired in the presence of 1 μM NT(8–13). Note: as no nonspecific binding of **21** was observed, images of nonspecific binding were only acquired after 45 min.

The excitation with 561 nm (**19**) resulted in higher autofluorescence compared to an excitation with 633 nm (**21**). This was only observable when using HT-29 cells (cf. Figures 6D and 7D) because the lower receptor expression in these cells compared to CHO-hNTS_1_R cells (cf. Figures S9D and S10D) necessitates higher laser power and higher detector gain (see Experimental Section). As becomes obvious from Figures 6C and 7C, showing nonspecific binding of **19** and **21**, respectively, the 5-TAMRA-labeled ligand **19** exhibited considerably higher nonspecific binding compared to the sulfo-Cy5-labeled probe **21**.

The microscopic studies with **19** and **21** revealed that the NTS_1_R is internalized by endocytosis upon binding of the fluorescent ligands, occurring already during the association process (Figures 6 and 7; Figures S9 and S10). The intracellular fluorescent ligand appeared to be located in vesicles. The internalization rates were estimated by defining an outer ROI, representing the total cellular fluorescence, and an inner ROI, representing the intracellular fluorescence, and quantification of fluorescence (Figure S11). This analysis suggests that the fraction of internalized ligand only slightly increases over the studied time (5–60 min). This could be explained by recycling of NTS_1_R to the plasma membrane after internalization as also suggested by previous studies of fluorescently labeled NT(8–13) analogs.^13^ It should be noted that the determined internalization rate represents only a rough estimation due to the visual definition of the ROIs and potentially biased fluorescence intensities caused by changes in the photophysical properties of the fluorescent ligands in the intracellular environment.

### Biomolecular Imaging of NTS_1_R Expression in Cells and Tumor Tissue

In another approach, the suitability of **19** and **21** to serve as probes for the investigation of NTS_1_R expression in living cells and tumor tissue was studied using an Azure Sapphire Biomolecular Imager. For these experiments, HT-29 cells, showing a low NTS_1_R expression,^12^ and HT-29 tumors subcutaneously grown in nude mice were used. Saturation binding experiments with the sulfo-Cy5-labeled ligand **21** at adherent HT-29 cells gave satisfactory fluorescence images revealing low nonspecific binding of **21** (Figure 8A). Based on the fluorescence intensities, saturation binding curves could be generated (Figure 8B) yielding a *K*_d_ value of 1.7 ± 1 nM (mean value ± SEM from four individual experiments performed in triplicate), which was higher than the *K*_d_ value obtained from flow cytometric saturation binding experiments at suspended HT-29 cells (cf. Table 4).

**Figure 8:**
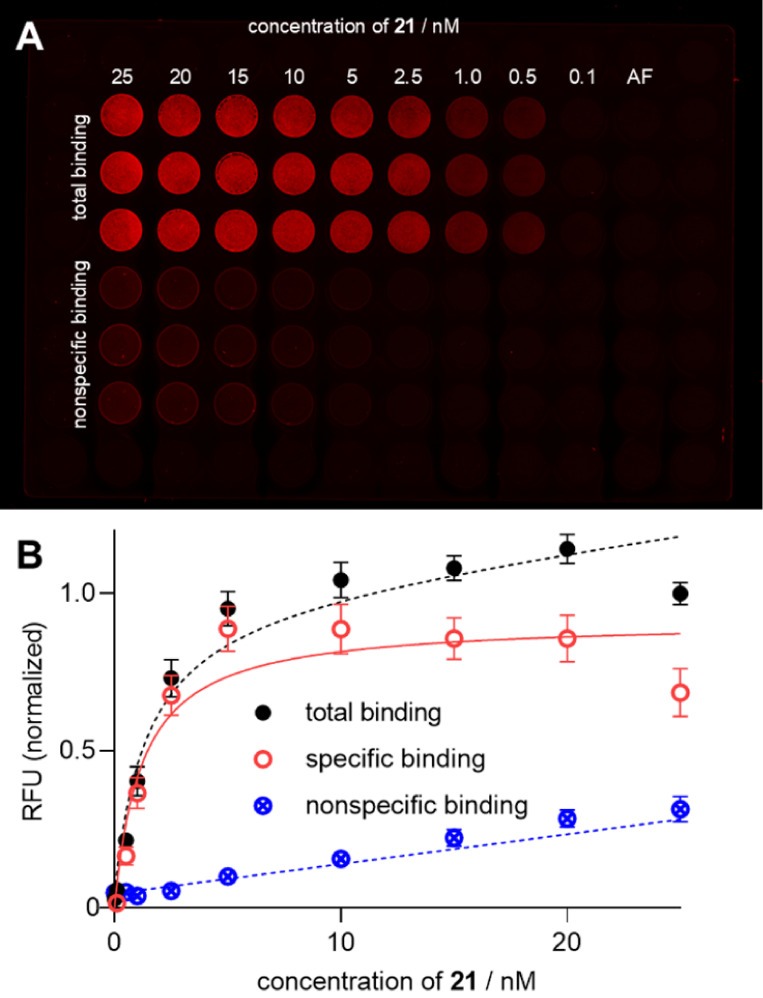
(A) Fluorescence image of a 96-well plate with adherent HT-29 cells acquired with an Azure Sapphire Biomolecular Imager (laser: 658 nm; pixel size: 20 μm) 2 h after incubation with **21** at rt. Nonspecific binding was determined in the presence of SR142948 (1 μM). Shortly before image acquisition, cells were washed three times with cold buffer. (B) Fluorescence intensities from (A) plotted against the concentration of **21**. Specific binding was calculated from total and nonspecific binding. Data represent means ± SEM (total and nonspecific binding) or calculated values ± propagated error (specific binding) from four independent experiments performed in triplicate.

This discrepancy could be explained by a lower sensitivity of the biomolecular imaging technique compared to the flow cytometric analysis, meaning that cell-bound fluorescence is underestimated for low ligand concentrations in the case of biomolecular imaging. Moreover, a higher adsorption of **21** to the plate material (polystyrene) and the required washing step in the case of the biomolecular imaging could account for the higher *K*_d_ value compared to the flow cytometric experiment which uses polypropylene plates and allows a measurement under equilibrium conditions (no washing required).

In contrast to **21**, the fluorescence images obtained from the same experiment performed with the 5-TAMRA-labeled ligand **19** were not suited for the determination of a *K*_d_ value due to high autofluorescence and high nonspecific binding (Figure S12).

Using the same biomolecular imager, visualization of NTS_1_R expression in HT-29 tumors was explored by imaging 10 μm cryosections of tumor tissue after incubation with **19** and **21**. As observed for the saturation binding experiment with HT-29 cells, a detection of NTS_1_R expression in the tumor was possible with the red-emitting probe **21** (Figure 9), but failed when using the 5-TAMRA-labeled ligand **19** as probe mainly due to high autofluorescence relative to specific binding (Figure S13). The regions of the tumor sections showing high nonspecific binding of **21** (Figure 9) likely represent necrotic tumor tissue. It should be noted that the HT-29 tumors originated from a former research project and had been stored for more than eight years at −80 °C.^12^ This long-term storage might have led to partial unfolding or degradation of the receptor protein.

**Figure 9:**
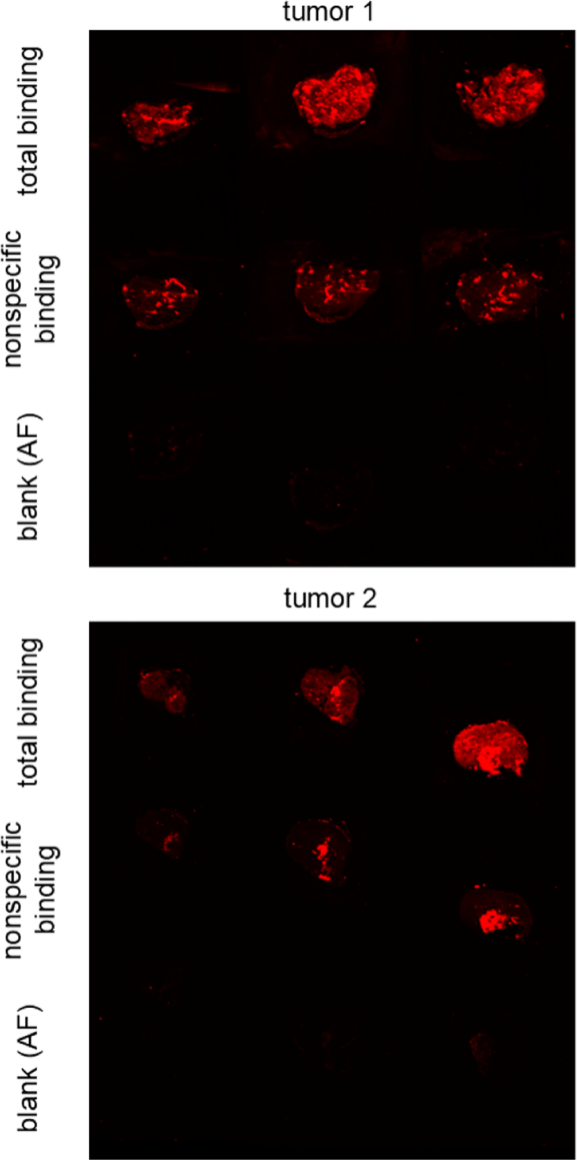
Fluorescence images of cryosections (size: 11–28 mm^2^, thickness: 10 μm) of a HT-29 tumor acquired after incubation with **21** (5 nM) at rt for 60 min with an Azure Sapphire FL Biomolecular Imager (laser: 658 nm; pixel size: 20 μm). Nonspecific binding was determined in the presence of SR142948 (5 μM). Blank samples were incubated with neat buffer to determine background signals. Shortly before image acquisition, tumor sections were washed three times with cold buffer.

## Conclusion

N-terminal methylation and the replacement of Arg^8^, Tyr^11^, and Leu^13^ in NT(8–13) by an amino-functionalized carbamoylated arginine, β,β-dimethyl-l-Tyr, and TMSAla, respectively, resulted in a NT(8–13) analog (**17a**, UR-FE083-I) that was used as a precursor for the preparation of a 5-TAMRA-labeled (**19**, UR-FE093) and a sulfo-Cy5-labeled (**21**, UR-FE094) NTS_1_R ligand. Both fluorescent ligands show subnanomolar NTS_1_R binding affinities with *K*_i_ values of 0.14 nM and 0.094 nM. The high binding affinity allows an application of these probes for flow cytometric binding assays using cells with low NTS_1_R expression levels, which was demonstrated for nontransfected HT-29 cells natively expressing the NTS_1_R.

The fluorescent peptides **19** and **21** display high *in vitro* proteolytic stability (*t*_1/2_ ≫ 48 h, human plasma), which is favorable with respect to binding studies in living cells potentially featuring proteolytic activities. **19** and **21** are anticipated to exhibit also high stability *in vivo* since recently described NT(8–13)-derived PET ligands, containing an N-terminal methyl group and β,β-dimethyl-l-Tyr in position 11, showed high *in vivo* stability in mice.^21^

Fluorescence imaging of receptor expression in tissues represents an attractive alternative to autoradiography that requires radiolabeled receptor ligands. The present study reveals that the 5-TAMRA-labeled ligand **19** is not suited for fluorescence-based imaging of the NTS_1_R expression in tumor tissue. This shows that the 5-TAMRA label, although characteristic of high photostability,^25^ does not meet the demands for this kind of imaging, in particular due to the excitation wavelength of <600 nm, resulting in interfering autofluorescence. Contrarily, the near-infrared sulfo-Cy5-labeled probe **21**, differing from **19** only with respect to the fluorescent dye, turned out to be an excellent molecular tool for NTS_1_R imaging in tumor tissue sections with high signal-to-noise ratio (used excitation: 658 nm), suggesting its application in tumor tissue screening procedures. As **19** and **21** display also high NTS_2_R binding affinity, they can potentially serve as probes for NTS_2_R binding studies using cellular systems solely expressing NTS_2_R.

One of the major challenges with respect to the *in vivo* use of labeled peptidic NTS_1_R ligands has been the development of tracers which exhibit both high proteolytic stability and high NTS_1_R binding affinity (*K*_i_ < 1 nM). With the development of the amine-functionalized precursor **17a**, which can be conjugated to various molecular labels, the present study paved the way to the future synthesis and use of high-affinity and proteolytically stable probes for the NTS_1_R with a functionality of choice.

## Experimental Section

### Materials

The protected amino acids Fmoc-Tyr­(*t*Bu)–OH, Fmoc-Arg­(Pbf)–OH, and glycine *tert*-butyl ester hydrochloride (**22**) were purchased from Carbolution Chemicals (St. Ingbert, Germany). Fmoc-N-Me-Arg­(Pbf)–OH (**11**), Fmoc-Lys­(Boc)–OH, Fmoc-Pro-OH, Fmoc-Ile-OH, and HBTU were from Merck (Darmstadt, Germany). Racemic Fmoc-β,β-dimethyl-Tyr­(*t*Bu)–OH (**12**) and 2-ClTrt resin were obtained from Iris Biotech (Marktredwitz, Germany). HOBt, 7-methyl-1,5,7-triazabicyclo[4.4.0]­dec-5-ene (MTBD), methyl-4-nitrobenzenesulfonate, 2-mercaptoethanol, and 1-methyl-d-Trp were from Sigma-Aldrich (Taufkirchen, Germany). Collidine, 2-nitrobenzenesulfonyl chloride, 1,8-diazabicyclo[5.4.0]­undec-7-ene (DBU), NMP and DMF for peptide synthesis, anhydrous DMF and NMP, dichloromethane, piperidine, and TFA were purchased from Fisher Scientific (Schwerte, Germany). (1*R*,2*R*,5*R*)-2-Hydroxy-3-pinanone (**23**) and (iodomethyl)­trimethylsilane (**25**) were from TCI (Eschborn, Germany). DIPEA was obtained from ABCR (Karslruhe, Germany). Acetonitrile (HPLC gradient grade) was from VWR (Ismaning, Germany). NT(8–13) was synthesized *via* SPPS in-house. SR142948 and SR48692 were purchased from Tocris Bioscience (Bristol, UK). SBI-553 was from Biocat (Heidelberg, Germany). Bacitracin, HEPES, and bovine serum albumin (BSA) were obtained from Serva (Heidelberg, Germany). Fetal bovine serum (FBS) was purchased from Pan-Biotech (Aidenbach, Germany). Fura-2 AM and Pluronic F-127 were obtained from Calbiochem/Merck Biosciences (Beeston, UK). The radioligand [^3^H]­UR-MK300 (molar activity: 2.41 TBq/mmol) and its unlabeled analog UR-MK300 were prepared according to a described procedure.^12^ Compounds **10** ^23^ and **13** ^12^ were prepared according to the reported procedures. 5-TAMRA succinimidyl ester (**18**) was purchased from Carl Roth (Karlsruhe, Germany). Sulfo-Cyanine5 NHS ester (S0586, **20**) was obtained from FEW Chemicals (Bitterfeld, Germany). Millipore water was consistently used for the preparation of stock solutions, buffers, and aqueous eluents for HPLC. Polypropylene reaction vessels with screw cap (1.5 or 2 mL) from Sarstedt (Nümbrecht, Germany) were used for small-scale reactions (e.g., activation of Fmoc-protected amino acids) and to keep stock solutions.

### NMR Spectroscopy

NMR spectra were recorded on an AVANCE 600 instrument with cryogenic probe (^1^H: 600 MHz; ^13^C: 150 MHz) and an AVANCE 400 instrument (^1^H: 400 MHz; ^13^C: 100 MHz) (Bruker, Karlsruhe, Germany). NMR spectra were calibrated based on the solvent residual peaks (^1^H NMR, DMSO-*d*_6_: δ = 2.50 ppm, CDCl_3_: δ = 7.26 ppm; ^13^C NMR, DMSO-*d*_6_: δ = 39.50 ppm, CDCl_3_: δ = 77.16 ppm) and data are reported as follows: ^1^H NMR: chemical shift δ in ppm (multiplicity [s = singlet, d = doublet, *t* = triplet, *m* = multiplet, br s = broad singlet], integral, coupling constant *J* in Hz); ^13^C NMR: chemical shift δ in ppm.

### Mass Spectrometry

High-resolution mass spectrometry (HRMS) was performed with an Agilent 6540 UHD accurate-mass Q-TOF LC/MS system coupled to an Agilent 1290 analytical HPLC system (Agilent Technologies. Santa Clara, CA) using an ESI source and the following LC method: column: Agilent Zorbax Eclipse Plus C18, 1.8 μm, 50 × 2.1 mm, column temperature: 40 °C, solvent/linear gradient: 0–4 min: 0.1% aqueous HCOOH/acetonitrile supplemented with 0.1% HCOOH 95:5–2:98, 4–5 min: 2:98, flow: 0.6 mL/min.

### Preparative HPLC

Preparative HPLC was performed with a system from Knauer (Berlin, Germany) consisting of two K-1800 pumps and a K-2001 detector (in the following referred to as “system 1”) or with a Prep 150 LC system from Waters (Eschborn, Germany) comprising a Waters 2545 binary gradient module, a Waters 2489 UV/vis detector, and a Waters fraction collector III (in the following referred to as “system 2”). A Gemini NX-C18, 5 μm, 250 mm × 21 mm (Phenomenex, Aschaffenburg, Germany) was used as stationary phase at a flow rate of 20 mL/min using mixtures of 0.1% aqueous TFA and acetonitrile as the mobile phase. A detection wavelength of 220 nm was used throughout. Collected fractions were lyophilized using a Scanvac CoolSafe 100–9 freeze-dryer (Labogene, Allerød, Denmark) equipped with a RZ 6 rotary vane vacuum pump (Vacuubrand, Wertheim, Germany).

### Analytical HPLC

Analytical HPLC analysis was performed with a system from Agilent Technologies composed of a 1290 Infinity binary pump equipped with a degasser, a 1290 Infinity autosampler, a 1290 Infinity thermostated column compartment, a 1260 Infinity diode array detector, and a 1260 Infinity fluorescence detector. A Kinetex-XB C18, 2.6 μm, 100 × 3 mm (Phenomenex) served as stationary phase at a flow rate of 0.6 mL/min. Detection was performed at 220 nm and the temperature of the column compartment was set to 25 °C. Mixtures of acetonitrile (A) and 0.04% aqueous TFA (B) were used as mobile phase. The following linear gradients were applied: compounds **6** and **14–17b**: 0–14 min: A/B 10:90–30:70, 14–15 min: 30:70–95:5, 15–18 min: 95:5 (isocratic); compounds **10**, **24**, **26**, and **27**: 0–20 min: A/B 10:90–90:10, 20–21 min: 90:10–95:5, 21–25 min: 95:5 (isocratic); compounds **19** and **21**: 0–14 min: A/B 20:80–40:60, 14–15 min: 40:60–95:5, 15–18 min: 95:5 (isocratic). The injection volume was 20 μL. Retention (capacity) factors *k* were calculated from the retention times *t*_R_ according to *k* = (*t*_R_ – *t*_0_)/*t*_0_ (*t*_0_ = dead time, 0.76 min for the used system and column).

### General Procedure for Solid Phase Peptide Synthesis

Peptides were synthesized manually by SPPS on a 2-ClTrt resin according to the Fmoc strategy. 5 mL NORM-JECT syringes (B. Braun-Melsungen, Melsungen, Germany), equipped with a 35-μm polypropylene frit (Roland Vetter Laborbedarf, Ammerbuch, Germany), were used as reaction vessels. DMF/NMP 4:1 v/v was used as solvent for all amino acid coupling and Fmoc deprotection steps. Prior to the coupling of the first amino acid (**10**), the 2-ClTrt resin (loading: 1.60 mmol/g) was allowed to swell in CH_2_Cl_2_ for 30 min at rt followed by washing of the resin with CH_2_Cl_2_ (2×). Amino acid **10** was attached to the resin according to a reported procedure.^46^ The loading of the resulting Fmoc-TMSAla-2-ClTrt resin, which was used for the synthesis of peptides **6** and **14–17b**, amounted to 1.00 mmol/g (determined photometrically *via* absorbance at 304 nm in DMF according to a reported protocol^47^). Fmoc deprotection of the Fmoc-TMSAla-2-ClTrt resin was carried out using 20% piperidine in DMF/NMP 4:1 v/v (2× 10 min at rt) followed by washing with solvent (6× ca. 1 mL). The following Fmoc-amino acids (except for **11**-**13**), used in 5-fold excess, were preactivated with HOBt/HBTU/DIPEA (5/4.9/10 equiv) in solvent (about 2.2 mL/mmol amino acid) for at least 5 min before addition to the resin. The Fmoc-protected unnatural amino acids **11**-**13** were used in 3-fold excess and were preactivated with HOBt/HBTU/DIPEA (3/3/6 equiv) in anhydrous solvent (about 1.6 mL/mmol amino acid) for 5–10 min prior to addition to the resin. Amino acid coupling was carried out on a shaker (Heidolph Multi Reax; Heidolph Instruments, Schwabach, Germany) covered with a thermostat controlled (35 °C) box. In the case of standard amino acids, “double” coupling (2× 45 min) was performed. **11**–**13** were attached by a single coupling procedure (35 °C, 16 h). After coupling of an Fmoc-amino acid, the resin was washed with solvent (4×) followed by Fmoc deprotection using 20% piperidine in solvent (2× 10 min at rt) and subsequent washing of the resin with solvent (6× ca. 1 mL). After coupling of the last amino acid and final Fmoc deprotection, the resin was washed with solvent (6×) and CH_2_Cl_2_ (3×) (treated with potassium carbonate). Peptides were cleaved off the resin using CH_2_Cl_2_/TFA 3:1 v/v (2× 20 min at rt). The liquids (2× ca. 2 mL) were collected in a 100 mL round-bottom flask and the resin was washed once with CH_2_Cl_2_/TFA 3:1 v/v (2 mL). The volatiles of the combined liquids were removed by evaporation, TFA/H_2_O 95:5 v/v (2 mL per 100 mg resin) was added to the residue and the mixture was stirred at rt for 5 h. The volatiles were removed by evaporation followed by the addition of H_2_O (ca. 50 mL) and lyophilization to obtain the crude peptide, which was subjected to purification by preparative HPLC.

### Compound Characterization

Amino acid **7**, peptides **14**–**17b**, and compounds **24** and **26** were characterized by HRMS, ^1^H-, ^13^C-, and 2D-NMR spectroscopy (2D: ^1^H-COSY, HSQC, HMBC), and RP-HPLC. Compounds **19** and **21** were characterized by HRMS, ^1^H NMR spectroscopy, and RP-HPLC. **6** and **27** were characterized by HRMS and RP-HPLC. HPLC purities of all target compounds were ≥97% (UV detection, 220 nm).

### Experimental Protocols and Analytical Data

#### Lys-Lys-Pro-Tyr-Ile-β-trimethylsilyl-Ala Tris­(hydrotrifluoroacetate) (**6**)^19^

Peptide **6** was synthesized on a Fmoc-TMSAla-2-ClTrt resin (50 mg, 1.00 mmol/g) according to the general procedure. Purification by preparative RP-HPLC (system 2, gradient: 0–30 min: acetonitrile/0.1% aqueous TFA 22:78–27:73, *t*_R_ = 7 min) yielded **6** as a white fluffy solid (29.4 mg, 52%). HRMS (ESI): *m*/*z* [M+3H]^3+^ calcd. for [C_38_H_69_N_8_O_8_Si]^3+^ 264.4997, found: 264.5001. RP-HPLC (220 nm): >99% (*t*_R_ = 8.6 min, *k* = 10.3). C_38_H_66_N_8_O_8_Si·C_6_H_3_F_9_O_6_ (794.10 + 342.07).

#### Arg-Arg-Pro-Tyr-Ile-β-trimethylsilyl-Ala Tris­(Hydrotrifluoroacetate) (**14**)

Peptide **14** was synthesized on a Fmoc-TMSAla-2-ClTrt resin (75 mg, 1.00 mmol/g) according to the general procedure. Purification by preparative RP-HPLC (system 1, gradient: 0–25 min: acetonitrile/0.1% aqueous TFA 15:85–45:55, *t*_R_ = 13 min) yielded **14** as a white fluffy solid (34.1 mg, 38%). ^1^H NMR (600 MHz, DMSO-*d*_6_): δ (ppm) 0.00 (s, 9H), 0.76–0.85 (m, 6H), 0.91–0.98 (m, 1H), 0.98–1.08 (m, 2H), 1.35–1.44 (m, 1H), 1.44–1.62 (m, 5H), 1.64–1.76 (m, 4H), 1.77–1.90 (m, 3H), 1.93–2.03 (m, 1H), 2.62–2.70 (m, 1H), 2.80–2.93 (m, 1H), 3.03–3.12 (m, 4H), 3.54–3.61 (m, 2H, interfering with the water signal, quantified in the spectrum acquired after the addition of D_2_O), 3.81–3.84 (m, 1H, interfering with the water signal, quantified in the spectrum acquired after the addition of D_2_O), 4.18–4.26 (m, 2H), 4.31–4.36 (m, 1H), 4.37–4.43 (m, 1H), 4.44–4.53 (m, 1H), 6.59–6.64 (m, 2H), 6.84–7.28 (br s, 4H, interfering with next listed signal), 6.99–7.03 (m, 2H), 7.28–7.65 (br s, 4H), 7.73 (d, 1H, *J* 9.2 Hz), 7.81 (t, 1H, 5.5 Hz), 7.86 (t, 1H, *J* 6.2 Hz), 7.94 (d, 1H, *J* 8.1 Hz), 8.13–8.29 (m, 4H), 8.67 (d, 1H, *J* 7.3 Hz), 9.23 (s, 1H), 12.45 (s, 1H). ^13^C NMR (150 MHz, DMSO-*d*_6_): δ (ppm) 0.00 (3 carbon atoms), 10.89, 15.13, 19.28, 24.03, 24.09, 24.17, 24.61, 28.21, 28.42, 29.10, 36.43, 37.27, 40.16, 40.53, 46.85, 48.63, 50.46, 51.69, 54.28, 56.32, 59.15, 113.96 (TFA), 114.85 (2 carbon atoms), 115.93 (TFA), 117.90 (TFA), 119.88 (TFA), 127.80, 130.11 (2 carbon atoms), 155.79, 156.92, 156.94, 158.88 (q, *J* 32 Hz) (TFA), 168.31, 169.24, 170.54, 170.75, 171.37, 174.61. HRMS (ESI): *m*/*z* [M + 3H]^3+^ calcd for [C_38_H_69_N_12_O_8_Si]^3+^ 283.1705, found: 283.1716. RP-HPLC (220 nm): >99% (*t*_R_ = 9.0 min, *k* = 10.8). C_38_H_66_N_12_O_8_Si·C_6_H_3_F_9_O_6_ (847.11 + 342.07).

#### *N* ^α^-Methyl-Arg-Arg-Pro-Tyr-Ile-β-trimethylsilyl-Ala Tris­(hydrotrifluoroacetate) (**15**)

Peptide **15** was synthesized on a Fmoc-TMSAla-2-ClTrt resin (75 mg, 1.00 mmol/g) according to the general procedure. Purification by preparative HPLC (system 1, gradient: 0–25 min: acetonitrile/0.1% aqueous TFA 15:85–45:55, *t*_R_ = 14 min) yielded **15** as a white fluffy solid (40.3 mg, 44%).^1^H NMR (600 MHz, DMSO-*d*_6_): δ (ppm) 0.00 (s, 9H), 0.76–0.86 (m, 6H), 0.91–0.98 (m, 1H), 0.98–1.07 (m, 2H), 1.38–1.48 (m, 3H), 1.51–1.59 (m, 3H), 1.66–1.76 (m, 4H), 1.79–1.87 (m, 3H), 1.94–2.04 (m, 1H), 2.46–2.48 (m, 3H), 2.62–2.69 (m, 1H), 2.82–2.88 (m, 1H), 3.06–3.15 (m, 4H), 3.55–3.60 (m, 2H, interfering with the water signal, quantified in the spectrum acquired after the addition of D_2_O), 3.78–3.81 (m, 1H, interfering with the water signal, quantified in the spectrum acquired after the addition of D_2_O), 4.17–4.28 (m, 2H), 4.32–4.36 (m, 1H), 4.38–4.43 (m, 1H), 4.52–4.57 (m, 1H), 6.60–6.63 (m, 2H), 6.82–7.29 (br s, 4H, interfering with next listed signal), 7.00–7.02 (m, 2H), 7.29–7.65 (br s, 4H), 7.71–7.75 (m, 1H), 7.84–7.90 (m, 2H), 7.93 (d, 1H, *J* 8.1 Hz), 8.20 (d, 1H, *J* 7.9 Hz), 8.87 (d, 1H, *J* 7.3 Hz), 8.95 (s, 1H), 9.04–9.70 (m, 2H), 12.46 (s, 1H). ^13^C NMR (150 MHz, DMSO-*d*_6_): δ (ppm) 0.00 (3 carbon atoms), 10.89, 15.13, 19.28, 23.92, 24.03, 24.18, 24.62, 26.99, 28.12, 29.09, 31.16, 36.42, 37.27, 40.08, 40.44, 46.86, 48.63, 50.58, 54.27, 56.32, 59.16, 59.92, 113.94 (TFA), 114.85 (2 carbon atoms), 115.91 (TFA), 117.88 (TFA), 119.86 (TFA), 127.80, 130.11 (2 carbon atoms), 155.78, 156.93 (2 carbon atoms), 158.91 (q, *J* 33 Hz) (TFA), 167.00, 169.06, 170.54, 170.74, 171.33, 174.60. HRMS (ESI): *m*/*z* [M + 3H]^3+^ calcd for [C_39_H_71_N_12_O_8_Si]^3+^ 287.8424, found: 287.8434. RP-HPLC (220 nm): >99% (*t*_R_ = 9.2 min, *k* = 11.1). C_39_H_68_N_12_O_8_Si·C_6_H_3_F_9_O_6_ (861.13 + 342.07).

#### *N* ^α^-Methyl-Arg-Arg-Pro-β,β-dimethyl-l-Tyr-Ile-β-trimethylsilyl-Ala Tris­(hydrotrifluoroacetate) (**16a**) and *N* ^α^-Methyl-Arg-Arg-Pro-β,β-dimethyl-d-Tyr-Ile-β-trimethylsilyl-Ala Tris­(hydrotrifluoroacetate) (**16b**)

Peptides **16a** and **16b**, representing diastereomers, were synthesized on a Fmoc-TMSAla-2-ClTrt resin (50 mg, 1.00 mmol/g) according to the general procedure. The unavailability of enantiomerically pure Fmoc-β,β-dimethyl-l-Tyr­(*t*Bu)–OH (this Fmoc amino acid was only available as racemic mixture) necessitated the synthesis of both diastereomers, which were separated by preparative HPLC (system 1, gradient: 0–25 min: acetonitrile/0.1% aqueous TFA 15:85–45:55, *t*_R_ (**16a**) = 14 min, *t*_R_ (**16b**) = 16 min). Lyophilization of the eluates yielded **16a** and **16b** as white fluffy solids (**16a**: 23.0 mg, 37%; **16b**: 21.1 mg, 34%). ^1^H NMR of **16a** (600 MHz, DMSO-*d*_6_): δ (ppm) 0.00 (s, 9H), 0.73–0.85 (m, 6H), 0.90–1.04 (m, 3H), 1.19–1.30 (m, 6H), 1.33–1.42 (m, 1H), 1.42–1.50 (m, 2H), 1.50–1.61 (m, 3H), 1.61–1.84 (m, 7H), 1.84–1.95 (m, 1H), 2.48 (s, 3H), 3.04–3.17 (m, 4H), 3.48–3.53 (m, 1H, interfering with the water signal, quantified in the spectrum acquired after the addition of D_2_O), 3.55–3.59 (m, 1H, interfering with the water signal, quantified in the spectrum acquired after the addition of D_2_O), 3.82 (s, 1H, interfering with the water signal, quantified in the spectrum acquired after the addition of D_2_O), 4.14 (t, 1H, *J* 8.4 Hz), 4.14–4.24 (m, 1H), 4.36–4.43 (m, 1H), 4.52–4.58 (m, 1H), 4.60–4.69 (m, 1H), 6.59–6.65 (m, 2H), 6.93–7.30 (br s, 4H, interfering with next listed signal), 7.11–7.14 (m, 2H), 7.30–7.81 (m, 6H), 7.84–7.94 (m, 2H), 8.08 (d, 1H, *J* 7.8 Hz), 8.92 (d, 1H, *J* 8.0 Hz), 8.95 (s, 1H), 9.04–9.41 (m, 2H), 12.42 (s, 1H). ^13^C NMR of **16a** (150 MHz, DMSO-*d*_6_): δ (ppm) 0.00 (3 carbon atoms), 10.94, 15.13, 19.22, 23.92, 24.11, 24.14, 24.45, 24.55, 26.99, 27.09, 28.13, 28.63, 31.16, 37.08, 40.06, 40.16, 40.43, 46.86, 48.68, 50.51, 56.43, 59.25, 59.88, 59.91, 114.07 (TFA), 114.34 (2 carbon atoms), 116.05 (TFA), 118.02 (TFA), 120.00 (TFA), 127.42 (2 carbon atoms), 136.42, 155.26, 156.90, 156.95, 158.83 (q, *J* 32 Hz) (TFA), 167.05, 169.35, 169.42, 170.43, 170.56, 174.66. ^1^H NMR of **16b** (600 MHz, DMSO-*d*_6_): δ (ppm) 0.00 (s, 9H), 0.71–0.82 (m, 6H), 0.88–1.03 (m, 3H), 1.17–1.34 (m, 8H), 1.40–1.49 (m, 2H), 1.49–1.64 (m, 5H), 1.64–1.85 (m, 5H), 2.47 (s, 3H), 3.01–3.17 (m, 4H), 3.41–3.50 (m, 1H, interfering with the water signal, quantified in the spectrum acquired after the addition of D_2_O), 3.51–3.58 (m, 1H, interfering with the water signal, quantified in the spectrum acquired after the addition of D_2_O), 3.80 (s, 1H, interfering with the water signal, quantified in the spectrum acquired after the addition of D_2_O), 4.10 (q, 1H, *J* 7.3 Hz), 4.13–4.19 (m, 1H), 4.35–4.42 (m, 1H), 4.47–4.54 (m, 1H), 4.87 (d, 1H, *J* 9.9 Hz), 6.57–6.67 (m, 2H), 6.74–7.26 (br s, 4H, interfering with next listed signal), 7.14–7.17 (m, 2H), 7.26–7.59 (br s, 4H), 7.62 (d, 1H, *J* 8.5 Hz), 7.69 (d, 1H, *J* 9.8 Hz), 7.79–7.90 (m, 2H), 8.19 (d, 1H, *J* 7.0 Hz), 8.85 (d, 1H, *J* 7.1 Hz), 8.94 (s, 1H), 9.00–9.56 (m, 2H), 12.43 (s, 1H). ^13^C NMR of **16b** (150 MHz, DMSO-*d*_6_): δ (ppm) 0.00 (3 carbon atoms), 11.09, 15.26, 19.25, 23.84, 23.99, 24.01, 24.15, 24.55, 26.53, 26.97, 28.02, 29.40, 31.14, 37.09, 40.09, 40.43, 41.00, 46.71, 49.31, 50.60, 56.41, 59.15, 59.49, 59.86, 114.06 (TFA), 114.35 (2 carbon atoms), 116.03 (TFA), 118.02 (TFA), 120.00 (TFA), 127.29 (2 carbon atoms), 136.86, 155.38, 156.92 (2 carbon atoms), 158.74 (q, *J* 32 Hz) (TFA), 166.95, 168.97, 169.40, 170.17, 170.84, 174.48. HRMS (ESI): *m*/*z* [M + 3H]^3+^ calcd for [C_41_H_75_N_12_O_8_Si]^3+^ 297.1861, found: 297.1866 (**16a**), 297.1866 (**16b**). RP-HPLC (220 nm): **16a**: 99% (*t*_R_ = 10.7 min, *k* = 13.1), **16b**: 97% (*t*_R_ = 12.2 min, *k* = 15.1). C_41_H_72_N_12_O_8_Si·C_6_H_3_F_9_O_6_ (889.19 + 342.07).

#### *N* ^α^-Methyl-*N* ^ω^-[(4-aminobutyl)­aminocarbonyl]­Arg-Arg-Pro-β,β-dimethyl-l-Tyr-Ile-β-trimethylsilyl-Ala Tetrakis­(hydrotrifluoroacetate) (**17a**) and *N* ^α^-Methyl-*N* ^ω^-[(4-aminobutyl)­aminocarbonyl]­Arg-Arg-Pro-β,β-dimethyl-d-Tyr-Ile-β-trimethylsilyl-Ala Tetrakis­(hydrotrifluoroacetate) (**17b**)

Peptides **17a** and **17b**, representing diastereomers, were synthesized on a Fmoc-TMSAla-2-ClTrt resin (150 mg, 1.00 mmol/g) according to the general procedure with the following modification: after coupling of the last amino acid and Fmoc-deprotection, the resin was washed with CH_2_Cl_2_ (5×), a solution of 2-nitrobenzenesulfonyl chloride (100 mg, 0.45 mmol) and collidine (100 μL, 0.75 mmol) in CH_2_Cl_2_ (3.5 mL) was added and the mixture was shaken at rt for 2 h. The resin was washed with DMF (5×) and a solution of MTBD (87 μL, 0.6 mmol) and methyl-4-nitrobenzenesulfonate (163 mg, 0.75 mmol) in DMF (4.2 mL) was added followed by shaking at rt for 30 min. After washing the resin with DMF (3×), a solution of DBU (112 μL, 0.75 mmol) and 2-mercaptoethanol (104 μL, 1.5 mmol) in DMF (3.5 mL) was added and the mixture was shaken at rt for 30 min. The resin was washed with DMF (5×) followed by cleavage from the resin as described in the general procedure for SPPS. The unavailability of enantiomerically pure Fmoc-β,β-dimethyl-l-Tyr­(*t*Bu)–OH (only available as racemic mixture) necessitated the synthesis of both diastereomers, which were separated by preparative RP-HPLC (system 1, gradient: 0–25 min: acetonitrile/0.1% aqueous TFA 15:85–45:55, *t*_R_ (**17a**) = 12 min, *t*_R_ (**17b**) = 14 min). Lyophilization of the eluates yielded **17a** and **17b** as white fluffy solids (**17a**: 30.1 mg, 14%; **17b**: 27.8 mg, 13%). ^1^H NMR of **17a** (600 MHz, DMSO-*d*_6_): δ (ppm) 0.00 (s, 9H), 0.72–0.89 (m, 6H), 0.90–1.04 (m, 3H), 1.18–1.31 (m, 6H), 1.33–1.42 (m, 1H), 1.43–1.61 (m, 9H), 1.62–1.94 (m, 8H), 2.47 (s, 3H), 2.73–2.83 (m, 2H), 3.03–3.18 (m, 4H), 3.21–3.30 (m, 2H), 3.46–3.54 (m, 1H), 3.54–3.63 (m, 1H), 3.76–3.91 (m, 1H), 4.12 (t, 1H, *J* 7.1 Hz), 4.16–4.25 (m, 1H), 4.36–4.43 (m, 1H), 4.48–4.59 (m, 1H), 4.66 (d, 1H, *J* 9.6 Hz), 6.57–6.65 (m, 2H), 6.71–7.28 (br s, 2H, interfering with next listed signal), 7.11–7.14 (m, 2H), 7.28–7.70 (m, 5H), 7.71–7.99 (m, 4H), 8.06 (d, 1H, *J* 7.9 Hz), 8.52 (s, 2H), 8.68–9.15 (m, 3H), 9.17 (s, 1H), 10.78 (s, 1H), 12.41 (s, 1H). One exchangeable proton (NH) of the presumably 4-fold protonated molecule could not be identified. ^13^C NMR of **17a** (150 MHz, DMSO-*d*_6_): δ (ppm) 0.00 (3 carbon atoms), 10.95, 15.14, 19.25, 23.43, 24.11, 24.16, 24.38, 24.54 (2 carbon atoms), 25.99, 27.00 (2 carbon atoms), 28.16, 28.64, 32.16, 37.08, 38.49, 38.64, 40.06, 40.14, 40.43, 46.86, 48.71, 50.50, 56.45, 59.25, 59.88 (2 carbon atoms), 114.12 (TFA), 114.35 (2 carbon atoms), 116.10 (TFA), 118.08 (TFA), 120.06 (TFA), 127.42 (2 carbon atoms), 136.40, 153.93 (2 carbon atoms), 155.27, 156.94, 158.90 (q, *J* 32 Hz) (TFA), 167.14, 169.32, 169.39, 170.42, 170.58, 174.69. ^1^H NMR of **17b** (600 MHz, DMSO-*d*_6_): δ (ppm) 0.00 (s, 9H), 0.70–0.82 (m, 6H), 0.87–1.02 (m, 3H), 1.19–1.35 (m, 8H), 1.44–1.65 (m, 11H), 1.65–1.83 (m, 5H), 2.47 (s, 3H), 2.75–2.83 (m, 2H), 3.04–3.16 (m, 4H), 3.21–3.30 (m, 2H), 3.43–3.50 (m, 1H), 3.50–3.58 (m, 1H), 3.81 (s, 1H), 4.11 (q, 1H, *J* 7.6 Hz), 4.15 (t, 1H, *J* 7.4 Hz), 4.36–4.41 (m, 1H), 4.49–4.55 (m, 1H), 4.86 (d, 1H, *J* 9.8 Hz), 6.57–6.65 (m, 2H), 6.73–7.32 (br s, 2H, interfering with next listed signal), 7.14–7.17 (m, 2H), 7.32–7.72 (m, 5H), 7.72–8.04 (m, 4H), 8.17 (d, 1H, *J* 7.0 Hz), 8.53 (s, 2H), 8.87 (d, 1H, *J* 7.3 Hz), 8.96 (s, 1H), 9.05–9.23 (m, 2H), 10.88 (s, 1H), 12.43 (s, 1H). One exchangeable proton (NH) of the presumably 4-fold protonated molecule could not be identified. ^13^C NMR of **17b** (150 MHz, DMSO-*d*_6_): δ (ppm) 0.00 (3 carbon atoms), 11.09, 15.26, 19.26, 23.37, 23.99, 24.02, 24.20, 24.39, 24.55, 25.99, 26.48, 26.91, 28.10, 29.32, 31.14, 37.10, 38.49, 38.66, 40.11, 40.44, 40.98, 46.71, 49.26, 50.59, 56.39, 59.17, 59.55, 59.81, 114.07 (TFA), 114.37 (2 carbon atoms), 116.04 (TFA), 118.01 (TFA), 119.98 (TFA), 127.29 (2 carbon atoms), 136.86, 153.94 (2 carbon atoms), 155.40, 156.97, 158.97 (q, *J* 32 Hz) (TFA), 166.92, 168.99, 169.34, 170.19, 170.81, 174.50. HRMS (ESI): *m*/*z* [M + 3H]^3+^ calcd. for [C_46_H_85_N_14_O_9_Si]^3+^ 335.2126, found: 335.2135 (**17a**), 335.2132 (**17b**). RP-HPLC (220 nm): **17a**: 97% (*t*_R_ = 9.5 min, *k* = 11.5), **17b**: 99% (*t*_R_ = 10.9 min, *k* = 13.3). C_46_H_82_N_14_O_9_Si·C_8_H_4_F_12_O_8_ (1003.34 + 456.09).

#### *N* ^α^-Methyl-*N* ^ω^-{[4-(*N*-{1-carboxylato­[2-(6-(dimethylamino)-3-(dimethyliminio))-3*H*-xanthen-9-yl]­phen-5-yl}­carbonyl)-aminobutyl]­aminocarbonyl}­Arg-Arg-Pro-β,β-dimethyl-l-Tyr-Ile-β-trimethylsilyl-Ala Tris­(hydro-trifluoroacetate) (**19**)

Peptide **17a** (3.4 mg, 2.3 μmol) and DIPEA (4.7 μL, 27 μmol) were dissolved in DMF/NMP 8:2 v/v (100 μL) followed by the addition of a solution of 5-TAMRA succinimidyl ester **18** (1.1 mg, 2.1 μmol) in DMF/NMP 8:2 v/v (30 μL). After stirring at room temperature in the dark for 1 h, the mixture was diluted with 10% aqueous TFA (14 μL) and acetonitrile/0.1% aqueous TFA 20:80 v/v (1 mL). Isolation by preparative RP-HPLC (system 1, gradient: 0–35 min: acetonitrile/0.1% aqueous TFA 20:80–60:40, *t*_R_ = 16 min) gave **19** as a purple fluffy solid (1.6 mg, 40%) ^1^H NMR (600 MHz, DMSO-*d*_6_): δ (ppm) 0.00 (s, 9H), 0.73–0.83 (m, 6H), 0.92–1.03 (m, 3H), 1.22–1.29 (m, 6H), 1.34–1.41 (m, 1H), 1.49–1.61 (m, 9H), 1.62–1.68 (m, 1H), 1.68–1.86 (m, 6H), 1.86–1.96 (m, 1H), 2.45–2.48 (m, 3H), 3.07–3.18 (m, 6H), 3.27 (s, 12H), 3.33–3.36 (m, 2H, interfering with the water signal, quantified in the spectrum acquired after the addition of D_2_O), 3.51–3.52 (m, 1H, interfering with the water signal, quantified in the spectrum acquired after the addition of D_2_O), 3.58–3.59 (m, 1H, interfering with the water signal, quantified in the spectrum acquired after the addition of D_2_O), 3.81–3.84 (m, 1H, interfering with the water signal, quantified in the spectrum acquired after the addition of D_2_O), 4.13 (t, 1H, *J* 7.8 Hz), 4.18–4.23 (m, 1H), 4.39–4.44 (m, 1H), 4.54–4.60 (m, 1H), 4.66 (d, 1H, *J* 9.1 Hz), 6.31–6.72 (br s, 1H, interfering with next listed signal), 6.59–6.64 (m, 2H), 6.82–7.17 (m, 9H), 7.18–7.48 (br s, 2H, interfering with next listed signal), 7.39 (d, 1H, *J* 9.3 Hz), 7.48–7.62 (m, 3H), 7.68 (t, 1H, *J* 5.1 Hz), 8.08 (d, 1H, *J* 7.6 Hz), 8.28 (d, 1H, *J* 7.6 Hz), 8.47 (s, 2H), 8.61–8.74 (m, 1H), 8.81–9.24 (m, 6H), 10.30 (s, 1H), 12.40 (s, 1H), 13.40 (s, 1H). HRMS (ESI): *m*/*z* [M + 3H]^3+^ calcd. for [C_71_H_105_N_16_O_13_Si]^3+^ 472.5933, found: 472.5942. RP-HPLC (220 nm): 99% (*t*_R_ = 8.8 min, *k* = 10.6). C_71_H_102_N_16_O_13_Si·C_6_H_3_F_9_O_6_ (1415.78 + 342.07).

#### *N* ^α^-Methyl-*N* ^ω^-{[4-(*N*-{6-3*H*-[2-(5–3*H*-(1-(4-sulfonatobutyl)-3,3-dimethyl)­indol-2-ylidenepent-1,3-dienyl)-3,3-dimethyl-5-sulfo]­indol-1-ium-1-yl}­hexanoyl)-aminobutyl]­aminocarbonyl}­Arg-Arg-Pro-β,β-dimethyl-l-Tyr-Ile-β-trimethylsilyl-Ala Tris­(hydrotrifluoroacetate) (**21**)

Peptide **17a** (3.3 mg, 2.3 μmol) and DIPEA (4.6 μL, 26 μmol) were dissolved in DMF/NMP 8:2 v/v (100 μL) followed by the addition of the sulfo-Cy5 succinimidyl ester **20** (1.1 mg, 2.1 μmol) in DMF/NMP 8:2 v/v (30 μL). After stirring at room temperature in the dark for 1 h, the mixture was diluted with 10% aqueous TFA (14 μL) and 0.1% acetonitrile/aqueous TFA 20:80 v/v (1 mL). Isolation by preparative RP-HPLC (system 1, gradient: 0–35 min: acetonitrile/0.1% aqueous TFA 20:80–60:40, *t*_R_ = 17 min) gave **21** as a purple fluffy solid (1.4 mg, 30%). ^1^H NMR (600 MHz, DMSO-*d*_6_): δ (ppm) 0.00 (s, 9H), 0.73–0.83 (m, 6H), 0.91–1.01 (m, 3H), 1.21–1.29 (m, 6H), 1.28–1.44 (m, 8H), 1.48–1.59 (m, 7H), 1.63–1.71 (m, 15H), 1.71–1.84 (m, 9H), 1.85–1.95 (m, 1H), 2.04 (t, 2H, *J* 6.4 Hz), 2.46–2.48 (m, 3H), 2.64 (t, 2H, *J* 6.8 Hz), 2.98–3.03 (m, 2H), 3.06–3.14 (m, 4H, interfering with the water signal, quantified in the spectrum acquired after the addition of D_2_O), 3.22–3.26 (m, 2H, interfering with the water signal, quantified in the spectrum acquired after the addition of D_2_O), 3.55–3.57 (m, 2H, interfering with the water signal, quantified in the spectrum acquired after the addition of D_2_O), 3.80–3.84 (m, 1H, interfering with the water signal, quantified in the spectrum acquired after the addition of D_2_O), 4.03–4.09 (m, 2H), 4.10–4.16 (m, 3H), 4.17–4.23 (m, 1H), 4.39–4.44 (m, 1H), 4.52–4.58 (m, 1H), 4.64 (d, 1H, *J* 9.3 Hz), 6.24 (d, 1H, *J* 13.7 Hz), 6.39 (d, 1H, *J* 14.0 Hz), 6.53–6.65 (m, 3H), 6.68–7.06 (br s, 2H), 7.09–7.16 (m, 2H), 7.20–7.32 (m, 3H), 7.32–7.58 (m, 7H), 7.61–7.67 (m, 2H), 7.78 (t, 1H, *J* 5.5 Hz), 7.81–7.84 (m, 1H), 8.08 (d, 1H, *J* 7.7 Hz), 8.25–8.48 (m, 4H), 8.74–9.05 (m, 4H), 9.06–9.18 (m, 1H), 9.99 (s, 1H), 12.40 (s, 1H). HRMS (ESI): *m*/*z* [M + 3H]^3+^ calcd. for [C_81_H_126_N_16_O_16_S_2_Si]^3+^ 557.2937, found: 557.2943. RP-HPLC (220 nm): 99% (*t*_R_ = 10.1 min, *k* = 12.3). C_81_H_123_N_16_O_16_S_2_Si·C_6_H_3_F_9_O_6_ (1669.18 + 342.07).

### Capillary Electrophoresis

Capillary electrophoresis was performed with an Agilent 7100 CE system using a bare fused silica capillary with an inner diameter of 0.05 mm and a length of 72 cm (G1600–62211, Agilent). 50 mM α-cyclodextrin (Honeywell, Charlotte, United States) in a 125 mM sodium phosphate buffer (pH = 7.0) served as electrolyte solution. Injections were performed hydrodynamically applying a pressure of 100 mbar for 10 s. The temperature of the capillary housing was set to 30 °C. A voltage of 20 kV was applied for 35 min and the detection was performed at 220 nm.

### Chemical Stability

The chemical stability of peptides **19** and **21** was investigated in PBS (adjusted to pH = 7.4) at 22 °C in the dark. The incubation was started by the addition of 3 μL of a 5 mM stock solution (solvent: DMSO) to 147 μL of PBS to yield a concentration of 100 μM. After periods of 0, 6, 24, and 48 h, an aliquot (25 μL) was removed and added to 25 μL of acetonitrile/0.04% aq. TFA 2:8 v/v to obtain a peptide solution with a concentration of 50 μM. 20 μL of this solution were subjected to analytical RP-HPLC analysis using the same system and conditions as described under *Analytical HPLC*. A 1:1 mixture of PBS and acetonitrile/0.04% aq. TFA 2:8 v/v (20 μL) was analyzed to obtain the blank chromatogram.

### Stability in Human Plasma

The proteolytic stabilities of NT(8–13), **15**, **17a**, **19** and **21** were investigated in human blood plasma/PBS 1:2 v/v according to a described procedure.^16^ Samples were analyzed using the RP-HPLC system and conditions as described under *Analytical HPLC* with the following gradient: 0–6 min: acetonitrile/0.04% aq. TFA 10:90–21:79, 6–12 min: 21:79–40:60, 12–13 min: 40:60–95:5, 13–16 min: 95:5 (isocratic). Data analysis was based on UV detection at 220 nm. Reference samples, representing 100% recovery, were prepared in quadruplicate. Recovery ratios were obtained by dividing the recovery of the peptide by the recovery of IS for each individual sample (*n* = 3–5). The obtained recoveries and the recovery ratios are summarized in Table S1.

### Excitation Spectra, Emission Spectra, and Fluorescence Quantum Yields

Absorption spectra were recorded with a referenced single beam spectrometer (Cary 60, Agilent) and the emission and excitation spectra were recorded in an orthogonal configuration in an emission spectrometer (Fluorolog, Horiba) setting the resolution to 1 nm for excitation and emission detection. The fluorescence decays were recorded with a self-constructed Time Correlated Single Photon Counting (TCSPC) setup^48^ with single detection wavelength at room temperature. The sample was excited along a 10 mm path length at λ_ex_ = 280 nm and the emission was recorded orthogonally to this along a 2 mm path length at λ_obs_ as indicated in Figure S7. The optical density of the sample was set to ca. 0.1 at the excitation wavelength over 10 mm path length. The fluorescence quantum yields of compounds **19** and **21** were determined in PBS and PBS supplemented with 1% BSA *via* an absolute method using an Ulbricht sphere with an inaccuracy of ca. 3% (Hamamatsu C9920–02 system equipped with a Spectralon integrating sphere) at room temperature using a 10 mm × 10 mm quartz cuvette. The optical density at the excitation wavelength of the sample was <0.1 along an optical path length of 1 cm.

### Cell Culture

Mammalian cells were cultured in T75 or T175 culture flasks (Sarstedt). Chinese hamster ovary (CHO) cells stably expressing hNTS_1_R^28^ were cultured in DMEM/HAM’s F12 (Sigma, Taufkirchen, Germany) medium (1:1) supplemented with 7.5% FBS, l-glutamine (Sigma) (630 μg/mL), and hygromycin B (Carl Roth, Karlsruhe, Germany) (250 μg/mL). HT-29 colon carcinoma cells (DSMZ-no. ACC 299) were grown in antibiotic-free RPMI-164 medium (Sigma) supplemented with 7.5% FBS or in MEM Earles medium supplemented with 10% FBS, 1% l-glutamine, 1% NEAA, and 1% pyruvate. HEK293T-hNTS_2_R cells were cultured in Dulbecco’s modified Eagle’s medium (Sigma-Aldrich) supplemented with 10% FBS, l-glutamine (2 mM) and penicillin–streptomycin (100 IU/mL and 0.1 mg/mL, respectively) (Sigma-Aldrich). All cell lines were cultured in a humidified atmosphere (95% air and 5% CO_2_) at 37 °C.

### Buffers Used for Binding and Functional Assays

*DPBS*: Dulbecco’s phosphate-buffered saline with calcium and magnesium (1.8 mM CaCl_2_, 2.68 mM KCl, 1.47 mM KH_2_PO_4_, 3.98 mM MgSO_4_, 137 mM NaCl, 8.06 mM Na_2_HPO_4_, pH = 7.4) supplemented with 0.1% BSA (flow cytometry) or 1% BSA (radiochemical assays) and 0.1 mg/mL bacitracin. For flow cytometric binding studies, *DPBS* was filtrated using 0.2 μm nylon syringe filters (Phenomenex).

*Fura-2 assay buffer*: HEPES buffer (120 mM NaCl, 5 mM KCl, 2 mM MgCl_2_, 1.5 mM CaCl_2_, 25 mM HEPES, and 10 mM glucose at pH = 7.4) supplemented with 2% BSA and 2.5 mM Probenecid (Sigma).

### Radiochemical Competition Binding Assays with [^3^H]­UR-MK300

NTS_1_R radioligand competition binding experiments with [^3^H]­UR-MK300 (molar activity: 2.41 TBq/mmol) and the peptides under study were performed with intact hNTS_1_R-expressing human HT-29 colon carcinoma cells at 23 ± 1 °C using a previously reported protocol.^12^ The *K*_d_ value of the radioligand for the used batch of [^3^H]­UR-MK300 was determined recently (*K*_d_ = 0.41 nM).^21^ Cells were seeded 1 day before the assay, yielding a confluency of at least 90% on the day of the experiment. Total binding data (including total binding in the absence of competitor) were plotted as dpm values against log­(concentration competitor) and analyzed by a four-parameter logistic equation (log­(inhibitor) vs response – variable slope, GraphPad Prism 5, GraphPad Software, San Diego, CA) to obtain pIC_50_ values. Individual pIC_50_ values were converted to p*K*_i_ values according to the Cheng-Prusoff equation^49^ (logarithmic form). To plot average data from individual binding experiments, data were normalized (100% = “top” of the four-parameter logistic fit, 0% = nonspecifically bound radioligand).

NTS_2_R radioligand competition binding experiments with [^3^H]­UR-MK300 were performed with membranes of HEK293T-hNTS_2_R.^21^ Membrane preparations were prepared using a recently reported protocol for membrane preparations of CHO-hNTS_1_R cells.^16^ The protein concentration amounted to 1.8 ± 0.2 mg/mL (mean value ± SEM from three different sample dilutions). NTS_2_R saturation and competition binding experiments with [^3^H]­UR-MK300 were carried out as described for saturation and competition binding studies with [^3^H]­UR-FE051 at membrane preparations of CHO-hNTS_1_R cells.^16^ The soluble protein concentration in the assays was 135 μg/mL. The *K*_d_ value of [^3^H]­UR-MK300 amounted to 3.1 ± 0.2 nM (mean value ± SEM from three independent experiments, each performed in triplicate). The concentration of [^3^H]­UR-MK300 in the competition binding assays was 5 nM. Data from competition binding assays were processed as the data from the NTS_1_R binding assays.

### Fura-2 Ca^2+^ Assay

The Fura-2 Ca^2+^ assay was conducted as previously described^16^ with the following modifications: HT-29 cells were used in place of CHO-hNTS_1_R cells and 1 μM NT(8–13) was used to determine the maximal response. Measurements were performed in black 96-well plates (Greiner 655076, Greiner Bio-One, Frickenhausen, Germany) and a measurement for one well comprised 40 cycles (instead of 44 cycles) with a cycle duration of 1.4 s (total time: 56 s).

### Flow Cytometric Binding Experiments

Flow cytometry-based NTS_1_R binding studies were performed with intact HT-29 (all kind of experiments) and CHO-hNTS_1_R cells (only saturation binding) using a BD FACSCanto II flow cytometer (Becton Dickinson, Heidelberg, Germany), equipped with an argon laser (488 nm), a red diode laser (640 nm), and a BD High Throughput Sampler (HTS unit) for microtiter plates. Saturation and competition binding experiments were performed in triplicate in 96-well polypropylene plates (Brand 701330, Wertheim, Germany) using the HTS unit for sample injection. Association and dissociation experiments were performed in triplicate in 5 mL polypropylene tubes (VWR, Radnor, USA), from which samples were directly injected into the flow cytometer. The following gain settings for forward and sideward scatter were applied: FSC, 0 V; SSC, 252 V. Fluorescence was recorded using the PE channel (excitation: 488 nm, emission: 585 ± 21 nm) with a PMT gain of 400–500 V (**19**) or the APC channel (excitation: 640 nm, emission: 660 ± 10 nm) with a PMT gain of 380–580 V (**21**). For measurements using the HTS unit, 45 μL of the sample were injected with a speed of 1.5 μL/s. For measurements, using an injection from 5 mL sample tubes, the medium flow rate (60 μL/min) was used. Measurements were stopped after 30 s (HTS unit) or after counting of at least 3000 gated events (injection from sample tubes).

Cells were seeded in T75 culture flasks 3–4 days (CHO-hNTS_1_R cells) or 5–6 days (HT-29 cells) prior to the experiment. On the day of the experiment, cells were detached by trypsinization, suspended in *DPBS* and centrifuged at 200 g at rt for 5 min. The cell pellet was resuspended in binding buffer and the cell density was adjusted to 1.5 × 10^4^ or 1.5 × 10^5^ cells/mL (CHO-hNTS1R cells) or 5.0 × 10^5^ cells/mL (HT-29 cells). Nonspecific binding was determined in the presence of 1 μM NT(8–13).

#### Saturation Binding Experiments

A 96-well polypropylene plate was prefilled with 200 μL of cell suspension. For total binding, H_2_O (2 μL) and DMSO/H_2_O 2:8 v/v (2 μL) containing the fluorescent ligand (100-fold concentrated compared to the final concentration) were added. To determine nonspecific binding, a 100 μM solution of NT(8–13) in H_2_O (2 μL) and DMSO/H_2_O 2:8 v/v (2 μL) containing the fluorescent ligand (100-fold concentrated) were added. Samples were incubated at 23 ± 1 °C in the dark under gentle shaking for 2 h followed by measurement *via* the HTS unit. Specific binding data, obtained by subtracting triplicate mean values of nonspecific binding from triplicate mean values of total binding, were plotted against the fluorescent ligand concentration and analyzed by a two-parameter equation describing hyperbolic single-site binding (one site, specific binding, GraphPad Prism 5) to obtain *K*_d_ values.

#### Association Experiments

5 mL polypropylene tubes were prefilled with 2000 μL of cell suspension and H_2_O (20 μL) was added (determination of total binding). To start the association, a 100-fold concentrated solution (compared to the final concentration) of the fluorescent ligand in DMSO/H_2_O 2:8 v/v (20 μL) was added (final concentrations: **19**: 1 nM, **21**: 0.1 nM). The sample tubes were gently shaken in the dark at 23 ± 1 °C. Measurements were conducted within different periods of time (1–240 min) by placing the tubes in the injection port of the cytometer. To determine nonspecific binding, samples were set up as in the case of total binding, but instead of 20 μL of H_2_O, 20 μL of a 100 μM solution of NT(8–13) in H_2_O were added. It should be noted that this experimental setup corresponds to a pseudo first-order measurement as the concentration of the free ligand, being markedly higher than the receptor concentration, can be considered constant by approximation during the association reaction. Specific binding data of **21**, obtained by subtracting triplicate mean values of nonspecific binding from triplicate mean values of total binding, were plotted against the time and analyzed by a three-parameter equation describing an exponential rise to a maximum (one-phase association, Y_0_ constrained to zero, GraphPad Prism 5) to yield the observed association rate constant *k*_obs_. To calculate mean values in %, specific binding data were normalized based on the corresponding *B*_eq_ value. Since specific binding data of **19** indicated a biphasic association, data of **19** were analyzed using a five-parameter two-phase association fit (two-phase association, Y_0_ constrained to zero, GraphPad Prism 5) to obtain the observed association rate constants *k*_obs(bi,fast)_ and *k*_obs(bi,slow)_ for the fast and slow phase of the association, respectively. To calculate mean values in % (cf. Figure 3B), specific binding data were normalized based on the corresponding *B*_eq_ values from the two-phase association fit (set to 100%). In addition to this analysis, the whole specific binding data of **19** were analyzed by a three-parameter equation describing an exponential rise to a maximum (one-phase association, Y_0_ constrained to zero, GraphPad Prism 5) to yield the observed association rate constant *k*_obs(mono)_.

#### Dissociation Experiments

5 mL polypropylene tubes were prefilled with 2000 μL of cell suspension. For the determination of total binding, the preincubation was started by the addition of H_2_O (20 μL) and a 100 nM solution of **19** or a 25 nM solution of **21** in DMSO/H_2_O 2:8 v/v (20 μL) (final fluorescent ligand concentration: 1 nM (**19**) or 0.25 nM (**21**)). To determine nonspecific binding, a 100 μM solution of NT(8–13) in H_2_O (20 μL) and a 100 nM solution of **19** or a 25 nM solution of **21** in DMSO/H_2_O 2:8 v/v were added. The samples were gently shaken in the dark at 23 ± 1 °C for 120 min (**19**) or for 180 min (**21**). The dissociation process was initiated by the addition of a 2.5 mM solution of NT(8–13) in H_2_O (20 μL) (final concentration: approximately 25 μM). After different periods of time (1–300 min (**19**) or 5–600 min (**21**)), sample aliquots were measured by placing the tube in the injection port of the flow cytometer. Specific binding data, obtained by subtracting triplicate mean values of nonspecific binding from triplicate mean values of total binding, were plotted against the time and analyzed by a three-parameter equation describing an incomplete monophasic exponential decline (one phase decay, GraphPad Prism 5) to obtain *k*_off_ values. For both fluorescent ligands, the mean ± SEM of the plateau values from individual experiments proved to be significantly different from zero (unpaired one-tailed *t* test, *P* > 0.05). To calculate mean values in %, binding data were normalized based on the specifically bound ligand measured immediately before the start of the dissociation.

#### Calculation of Association Rate Constants (*k*_on_) and Kinetically Derived Dissociation Constants *K*_d_(kin)

The association rate constants were calculated from *k*_obs_ mean values, *k*_off_ mean values, and the fluorescent ligand concentration used for the association experiments ([FL]) according to the equation *k*_on_ = (*k*_obs_ – *k*_off_)/[FL]. The kinetically derived dissociation constants *K*_d_(kin) were calculated from the respective *k*_on_ value and the *k*_off_ mean value according to *K*_d_(kin) = *k*_off_/*k*_on_.

#### Competition Binding Experiments

A 96-well polypropylene plate was prefilled with 200 μL of cell suspension. 2 μL of DMSO/H_2_O 2:8 v/v (for the determination of total binding in the absence of competitor), 2 μL of a 100 μM solution of NT(8–13) in H_2_O (determination of nonspecific binding) or 2 μL of a 100-fold concentrated solution (compared to the final concentration) of the compound of interest (NT(8–13), SR142948, SR48692 or UR-MK300; used at varying concentrations) in DMSO/H_2_O 2:8 v/v were added and the plate was shortly shaken. Subsequently, 2 μL of a 30 nM solution of **19** or a 10 nM solution of **21** in DMSO/H_2_O 2:8 v/v were added to each well and the plate was gently shaken in the dark at 23 ± 1 °C for 120 min (**19**) or 180 min (**21**) followed by measurement *via* the HTS unit. The final concentrations of **19** and **21** corresponded to their 3-fold or 2-fold *K*_d_ values determined by equilibrium saturation binding (*K*_d_ = 0.11 nM (**19**) or 0.046 nM (**21**)). Total binding fluorescence intensities (including total binding in the absence of competitor) were plotted against log (concentration inhibitor) and analyzed by a four-parameter logistic equation (log­(inhibitor) vs response-variable slope, GraphPad Prism 5) to obtain pIC_50_ values. Individual pIC_50_ values were converted to p*K*_i_ values according to the Cheng-Prusoff equation^49^ (logarithmic form). To plot average data from individual binding experiments, data were normalized (100% = “top” of the four-parameter logistic fit, 0% = nonspecifically bound fluorescent ligand).

#### Binding Experiments with SBI-553

A 96-well polypropylene plate was prefilled with 200 μL of cell suspension. 2 μL of DMSO/H_2_O 2:8 v/v (determination of total binding in the absence of SBI-553) or 2 μL of a 100-fold concentrated solution (compared to the final concentration) of SBI-553 (used at varying concentrations) in DMSO/H_2_O 2:8 v/v (determination of total binding in the presence of SBI-553) were added and the plate was incubated at 23 ± 1 °C for 30 min. Subsequently, 2 μL of a 1 nM solution of **21** in DMSO/H_2_O 2:8 v/v (final concentration: 0.01 nM) were added. To determine nonspecific binding, 2 μL of a 100 μM solution of NT(8–13) in H_2_O and 2 μL of a 1 nM solution of **21** in DMSO/H_2_O 2:8 v/v were added. The plate was then gently shaken in the dark at 23 ± 1 °C for 180 min followed by measurement *via* the HTS unit. Total binding fluorescence intensities (including total binding in the absence of the allosteric modulator) were plotted against log (concentration SBI-553). As the data clearly indicated a biphasic course, data were analyzed by a five-parameter two sites—fit logIC_50_ (GraphPad Prism 5) to obtain pIC_50_ (corresponding to pEC_50_) values for the high- and low-affinity state (the biphasic fit was favored over the four-parameter logistic fit according to the *F*-test, *P* < 0.05, GraphPad Prism 5). To plot average data from individual binding experiments, data were normalized (100% = lower plateau of the initial biphasic fit, 0% = nonspecifically bound fluorescent ligand). The differences between the lower and upper plateaus of the individual experiments gave the *E*_max_ values which were averaged to give the *E*_max_ mean ± SEM.

### Confocal Microscopy

Confocal microscopy was performed with a Zeiss LSM 710 confocal laser scanning microscope (Zeiss, Jena, Germany). The objective was 63× magnification with oil (1.4 NA). One day prior to the experiment, CHO-hNTS_1_R (30,000 to 35,000 cells per chamber) and HT-29 cells (45,000 to 50,000 cells per chamber) were seeded in Nunc LabTekTM II cover glasses with 8 chambers (Thermo Fisher Scientific). On the day of the experiment, the confluency of the cells was 60–80%. After removal of the culture medium, cells were washed with Leibovitz’s L15 medium (200 μL) and covered with L15 medium (150 μL) containing H33342 (Sigma-Aldrich) (2 μg/mL). To study total binding, L15 medium (150 μL) containing H33342 (2 μg/mL) and **19** or **21** (final concentration: 2 nM) was added. To determine nonspecific binding, L15 medium (150 μL) containing H33342 (2 μg/mL), NT(8–13) (final concentration: 1 μM) and **19** or **21** was added. The first image was acquired 5 min after addition of the fluorescent ligand. The pinhole was set to 1.0 airy unit. Laser powers and gains for the investigation of **19**: 405 nm, 2% (gain: 700 V); 561 nm, 8% (gain: 800 V) (CHO-hNTS_1_R cells) or 10% (gain: 850 V) (HT-29 cells). Laser powers and gains for the study of **21**: 405 nm, 2% (gain: 650 V (HT-29 cells) or 750 V (CHO-hNTS_1_R cells)); 633 nm, 20% (gain: 1050 V) (CHO-hNTS_1_R cells) or 40% (gain: 1100 V) (HT-29 cells). The following filter settings for fluorescence detection were applied: 410–549 nm (H33342, experiments with **19**), 410–585 nm (H33342, experiments with **21**), 562–649 nm (**19**), and 638–759 nm (**21**).

### Biomolecular Imaging

#### Saturation Binding Studies at HT-29 Cells

HT-29 cells were seeded 1 day prior to the experiment (1.0 × 10^5^ cells/well) in black clear bottom 96-well plates (Greiner 655090, Greiner Bio-One). After washing the cells with Leibovitz’s L-15 Medium (Thermo Fisher Scientific, Waltham, MA, USA), 100 μL of L-15 containing **19** or **21** in various concentrations (0.1–100 nM (**19**) or 0.1–25 nM (**21**), each in triplicates) followed by incubation at rt in the dark for 2 h. To determine nonspecific binding, SR142948 (1 μM; Sigma-Aldrich) was added to the samples. For the determination of the autofluorescence only L-15 was added to the cells. In addition, CellTag 520 or 700 Stain (LI-COR Biotechnology, Bad Homburg, Germany) was added to each well (final concentrations: 1 μM and 0.2 μM, respectively). After incubation, the plate was set on ice, the cells were washed three times with ice-cold DPBS (Sigma-Aldrich), the buffer was removed, and fluorescence images were acquired using the Sapphire FL Biomolecular Imager (520 and 658 nm laser; pixel size: 20 μm; Azure Biosystems, Dublin, CA, USA). The following bandpass filters were used for fluorescence detection: 565/24 nm (**19**) or 710/40 nm (**21**). Fluorescence intensities for each well were normalized to the respective CellTag fluorescence. Specific binding data, obtained by subtracting triplicate mean values of nonspecific binding from triplicate mean values of total binding, were plotted against the ligand concentration and *K*_d_ values were obtained by fitting of the data with a two-parameter equation describing hyperbolic single-site binding (one site, specific binding, GraphPad Prism 9). Four independent experiments were performed in triplicate.

#### Fluorescent Imaging of Tumor Slices

NMRI (nu/nu) mice were bred in the animal facility of the University of Regensburg. All animal experiments were performed following the protocols evaluated and approved by the local veterinary medicine authority—Regierung der Oberpfalz, Bavaria, Germany (approval number 2532.4-11/11). HT-29 tumor slices (10 μm) were prepared from HT-29 tumors (subcutaneously grown in 3–6 months old male NMRI nude (nu/nu) mice) using a cryostat microtome HM 500 O (Microm, Walldorf, Germany) and thaw-mounted on HistoBond adhesive glass slides (Marienfeld, Lauda-Königshofen, Germany). Tumor slices used for the determination of total binding were covered with buffer (DPBS supplemented with 1.8 mM CaCl_2_ and 3.98 mM MgCl_2_) at rt for 15 min. Tumor slices used for the determination of nonspecific binding were preincubated with buffer containing SR142948 (5 μM in buffer). The tumor slices were then incubated with **19** (10 nM) or **21** (5 nM) in buffer to determine total binding. For the determination of nonspecific binding, the samples contained additionally SR142948 (5 μM). Tumor slices were incubated with neat buffer to determine background signals (autofluorescence). After 1 h of incubation, the tumor slices were washed three times with ice-cold buffer, once with cold water, and were allowed to dry prior to acquisition of fluorescence images as described under *Saturation binding studies at HT-29 cells*. Data analysis was performed using the Aida Image Analyzer (v.5.1, Elysia-raytest GmbH, Straubenhardt, Germany).

### Calculation of Propagated Errors

Propagated errors (applying to specifically bound fluorescent ligand (saturation binding)), association rate constants *k*_on_, and kinetically derived dissociation constants *K*_d_(kin) were calculated as described elsewhere.^50^

## Supplementary Material





## Notes

Synthesis of compounds **10**, **24**, **26**, and **27** (Scheme S1) and experimental protocols and analytical data of **10**, **24**, **26**, and **27**; structures and NTS_1_R binding affinities of reported NT(8–13) derivatives containing β,β-dimethyl-l-Tyr or β,β-dimethyl-d-Tyr in position 11 (Figure S1); chemical stability of **19** and **21**: chromatograms of the HPLC analyses (Figures S2 and S3); structure of [^3^H]­UR-MK300 (Figure S4); radioligand displacement curves from competition binding experiments with [^3^H]­UR-MK300 (Figure S5); concentration response curves of NT(8–13), **19**, and **21** obtained from a Fura-2 Ca^2+^ assay (Figure S6); absorption, emission, and excitation spectra, and graphical presentation of the fluorescence decay of **19** and **21** (Figure S7); representative binding isotherms of **19** and **21** obtained from flow cytometric saturation binding experiments performed at intact CHO-hNTS_1_R cells (Figure S8); visualization of binding of **19** to intact CHO-hNTS_1_R cells by confocal microscopy (Figure S9); visualization of binding of **21** to intact CHO-hNTS_1_R cells by confocal microscopy (Figure S10); confocal microscopy: estimation of total cellular fluorescence and intracellular fluorescence (Figure S11); fluorescence image of a 96-well plate with adherent HT-29 cells acquired with a Biomolecular Imager after incubation with **19** (Figure S12); fluorescence images of cryosections of a HT-29 tumor acquired after incubation with **19** (Figure S13); recoveries of **19** and **21** from human plasma/PBS 1:2 v/v and ratios of compound-recovery over recovery of internal standard (Table S1); electropherograms of the CE analysis of (*R*,*S*)-**10** and (*R*)-**10**; RP-HPLC chromatograms of **6**, **10**, **14**–**17b**, **19**, and **21** (purity controls); ^1^H NMR spectrum and^13^C NMR spectrum of compound **10**; ^1^H NMR spectra of compounds **14**–**17b**, **19**, and **21** and ^13^C NMR spectra of compounds **14**–**17b** (PDF)
Molecular formula strings and NTS_1_R receptor affinities (CSV)

## ∇

Roche Diagnostics GmbH, Nonnenwald 2, 82377 Penzberg, Germany

## Abbreviations

AF: autofluorescence
Arg­(carb): *N* ^ω^-carbamoylated arginine
CE: capillary electrophoresis
CEC: concentration-effect curve
CH_2_Cl_2_: dichloromethane
CHO cells: Chinese hamster ovary cells
2-ClTrt: 2-chlorotrityl
DPBS: Dulbecco’s phosphate buffered saline
DIPEA: diisopropylethylamine
FBS: fetal bovine serum
FC: flow cytometry
HBTU: [bis­(dimethylamino)­methyliumyl]-3*H*-benzotriazol-1-oxide hexafluorophosphate
HOBt: hydroxybenzotriazole
*K*_d_: dissociation constant from a saturation binding experiment
*k*_obs_: observed association rate constant
*k*_off_: dissociation rate constant
*k*_on_: association rate constant
MTBD: methyl-1,5,7-triazabicyclo­[4.4.0]­dec-5-ene
NEAA: non essential amino acid solution
NTS_1_R: neurotensin receptor type 1
pEC_50_: negative decadic logarithm of the half maximal effective concentration (functional assays)
p*K*_i_: negative decadic logarithm of the dissociation constant *K*_i_ (in nM) obtained from a competition binding experiment
ROI: region of interest
SEM: standard error of the mean
SPPS: solid phase peptide synthesis
TMSAla: (trimethylsilyl)­alanine
