# Supplementary material for: High-Affinity and Proteolytically Stable Peptidic Fluorescent NTS1R Ligands
Source: J Med Chem. 2025 Sep 3;68(18):19482–502. doi: 10.1021/acs.jmedchem.5c01701 (PMC12481580; doi:10.1021/acs.jmedchem.5c01701)
Supplement: Supplementary file 1 [file jm5c01701_si_001.pdf]

Supporting Information

High-Affinity and Proteolytically Stable Peptidic Fluorescent NTS_1R Ligands

Fabian J. Ertl,[a] *Anna Friedel,*[b] *Elena J. Schmid,*[a] *Carina Höring,*[a,#] *Nataliya Archipowa,*[c] *Pierre Koch,*[a] *Simone Maschauer,*[b,e] *Roger J. Kutta,* *[*,d] *Olaf Prante,* *[*,b,e,f] *and Max Keller*[*,a]

[a]Institute of Pharmacy, Faculty of Chemistry and Pharmacy, University of Regensburg, Universitätsstraße 31, D-93053 Regensburg, Germany

[b]Department of Nuclear Medicine, Molecular Imaging and Radiochemistry, Friedrich-Alexander-Universität Erlangen-Nürnberg (FAU), Kussmaulallee 12, D-91054 Erlangen, Germany.

[c]Institute of Biophysics and Physical Biochemistry, Faculty of Biology and Preclinical Medicine, University of Regensburg, Universitätsstraße 31, D-93053 Regensburg, Germany

[d]Institute of Physical and Theoretical Chemistry, Faculty of Chemistry and Pharmacy, University of Regensburg, Universitätsstraße 31, D-93053 Regensburg, Germany

[e]Bavarian Cancer Research Center (BZKF), Translational Research Group TRAFO, D-91054 Erlangen and D-93053 Regensburg, Germany

[f]FAU NeW - Research Center New Bioactive Compounds, Friedrich-Alexander-Universität Erlangen-Nürnberg (FAU), D-91058 Erlangen, Germany

*E-mail addresses: max.keller@chemie.uni-regensburg.de
olaf.prante@uk-erlangen.de
roger-jan.kutta@ur.de

Content Page

1. Synthesis of Fmoc-L-(trimethylsilyl)alanine ((*R)*-**10**) S3

2. Figures S1-S13 S5

3. Table S1 S13

4. Electropherograms of the analysis of (*R*,*S*)-**10** and (*R*)-**10** by CE S14

5. RP-HPLC chromatograms of **6**, **10**, **14-17b**, **19**, and **21** (purity controls) S15

6. ^{1}H-NMR spectrum and ^{13}C-NMR spectrum of compound **10** in $CDCl_3$ S19

7. ^{1}H-NMR spectra of compounds **14-17b**, **19**, and **21** and ^{13}C-NMR spectra of compounds **14-17b** in DMSO-d_6 and DMSO-d_6/D_2O 4:1 v/v S20

8. References S31

1. Synthesis of Fmoc-L-(trimethylsilyl)alanine ((*R*)-10)

Scheme S1. Asymmetric synthesis of (*R*)-**10** according to a reported procedure.[1]

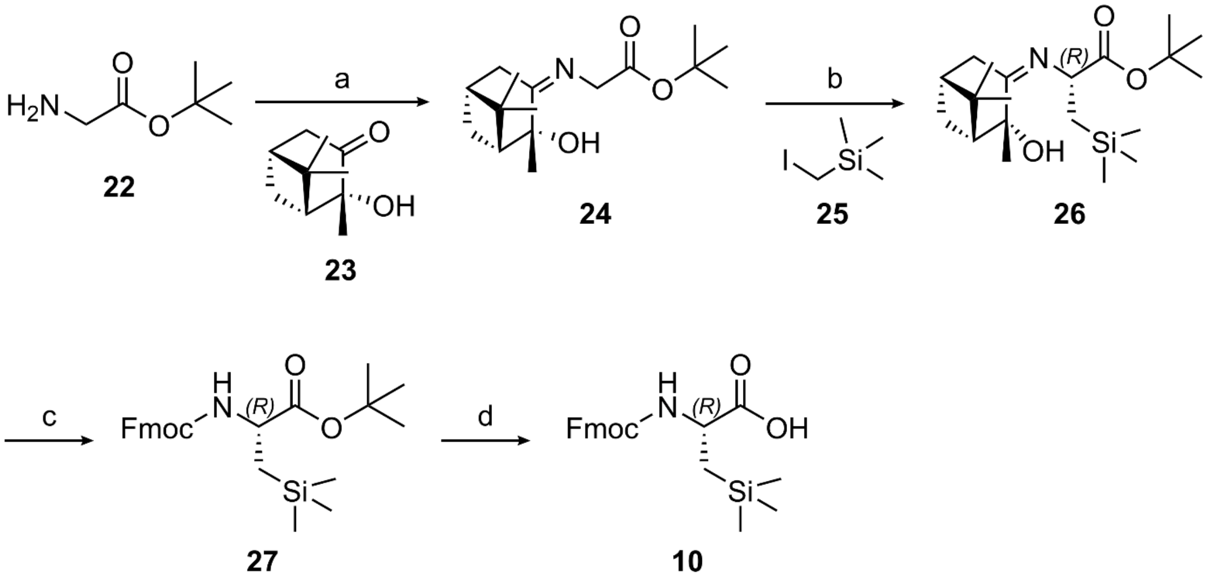

Reagents and conditions: (a) (1) Et_3N, toluene, rt, 1h; (2) **23**, $Et_2O \cdot BF_3$, toluene, reflux, 4 h; (b) (1) LDA, THF, -78 °C, 40 min; (2) **25**, -78 °C, 8 h; (3) -10 °C, overnight; (c) (1) 15% aqueous citric acid solution/THF 75:25 (v/v), rt, 3 days; (2) FmocOSu, Et_3N, ethyl acetate, rt, 6 h; (c) TFA, rt, 1 h.

Experimental protocols and analytical data

***Tert*-butyl 2-(((1*R*,2*R*,5*R*,*Z*)-2-hydroxy-2,6,6-trimethylbicyclo[3.1.1]heptan-3-ylidene)amino)acetate (24).**[1] Compound **24** was synthesized according to the reported procedure[1] using 3.0 g of glycine *tert*-butyl ester hydrochloride (**22**) (17.9 mmol) and 2.0 g of (1*R*,2*R*,5*R*)-2-hydroxy-3-pinanone (**23**) (11.9 mmol). Purification was slightly adapted using light petroleum/diethyl ether with 1% Et_3N (3:1 (v/v) to 1:1 (v/v)) as eluent mixture. **24** was obtained as yellow oil (0.97 g, 29%). ^{1}H-NMR (400 MHz, $CDCl_3$): δ (ppm) 0.86 (s, 3H), 1.32 (s, 3H), 1.48 (s, 9H), 1.51 (s, 3H), 1.56 (d, 1H, *J* 10.7 Hz), 1.99-2.10 (m, 2H), 2.29-2.37 (m, 1H), 2.41-2.54 (m, 2H), 2.55-2.70 (br s, 1H), 4.02-4.13 (m, 2H). ^{13}C-NMR (100 MHz, $CDCl_3$): δ (ppm) 22.90, 27.32, 28.12 (4 carbon atoms), 28.29, 33.62, 38.28, 38.55, 50.26, 53.45, 76.50, 81.30, 169.31, 179.63. HRMS (ESI): *m/z* $[M+H]^+$ calcd. for $[C_{16}H_{28}NO_3]^+$ 282.2064, found: 282.2067. RP-HPLC (220 nm): > 97% (t_R = 5.5 min, k = 6.2). $C_{16}H_{27}NO_3$ (281.40).

***Tert*-butyl (*R*)-2-(((1*R*,2*R*,5*R*,*Z*)-2-hydroxy-2,6,6-trimethylbicyclo[3.1.1]heptan-3-ylidene)amino)-3-(trimethylsilyl)propanoate (26).**[1] Compound **26** was synthesized according to the reported procedure[1] using 1.5 g of **24** (5.2 mmol) and 1.4 mL of (iodomethyl)trimethylsilane (**25**) (9.4 mmol). Purification was slightly adapted using light petroleum/diethyl ether with 1% Et_3N (6:1 (v/v) to 2:1 (v/v)) as eluent mixture. **26** was obtained as yellow oil (0.64 g, 34%). ^{1}H-NMR (400 MHz, $CDCl_3$): δ (ppm) 0.02 (s, 9H), 0.86 (s, 3H), 1.21-1.25 (m, 2H), 1.33 (s, 3H), 1.44 (s, 9H), 1.48 (s, 3H), 1.56 (d, 1H, *J* 10.7 Hz), 1.98-2-05 (m, 1H), 2.05-2.10 (m, 1H), 2.28-2.37 (m, 1H), 2.45-2.59 (m, 2H), 2.62 (s, 1H), 4.15-4.21 (m, 1H). ^{13}C-NMR (100 MHz, $CDCl_3$): δ (ppm) 0.00 (3 carbon atoms), 21.35, 22.97, 27.50, 28.14 (3 carbon atoms), 28.32, 28.39, 33.15, 38.37, 38.50, 50.26, 60.56, 76.63, 81.21, 172.28, 177.71. HRMS (ESI): *m/z* $[M+H]^+$ calcd. for $[C_{20}H_{38}NO_3Si]^+$ 368.2616, found: 268.2622. RP-HPLC (220 nm): > 86% (t_R = 10.7 min, *k* = 13.1). $C_{20}H_{37}NO_3Si$ (367.61).

***Tert*-butyl (*R*)-2-((((9*H*-fluoren-9-yl)methoxy)carbonyl)amino)-3-(trimethylsilyl)propanoate (27).**[1] Compound **27** was synthesized according to the reported procedure[1] using 0.64 g of **26** (1.7 mmol) and 0.76 g of FmocOSu (2.3 mmol). **27** was obtained as yellow oil (0.56 g, 76%). HRMS (ESI): *m/z* $[M+Na]^+$ calcd. for $[C_{25}H_{33}NNaO_4Si]^+$ 462.2072, found: 462.2083. RP-HPLC (220 nm): > 94% (t_R = 18.6 min, *k* = 23.5). $C_{25}H_{33}NO_4Si$ (439.63).

(*R*)-2-((((9*H*-Fluoren-9-yl)methoxy)carbonyl)amino)-3-(trimethylsilyl)propanoic acid (10).[1] Compound **10** was synthesized according to the reported procedure[1] using 0.56 g of **27** (1.3 mmol). **10** was obtained as crystalline foam (0.41g, 85%). ^{1}H-NMR (400 MHz, $CDCl_3$): δ (ppm) 0.07 (s, 9H), 0.95-1.04 (m, 1H), 1.17-1.25 (m, 1H), 4.22 (t, 1H, *J* 7.1 Hz), 4.38-4.44 (m, 2H), 5.13 (d, 1H, *J* 8.7 Hz), 7.30 (t, 2H, *J* 7.8 Hz), 7.39 (t, 2H, *J* 7.1 Hz), 7.59 (t, 2H, *J* 7.7 Hz), 7.76 (t, 2H, *J* 7.4 Hz), 7.92-8.98 (br s, 1H). The proton signal of the carboxylic acid was not apparent. ^{13}C-NMR (100 MHz, $CDCl_3$): δ (ppm) 0.00 (3 carbon atoms), 21.04, 47.28, 51.24, 67.20, 120.13 (2 carbon atoms), 125.16, 125.23, 127.22 (2 carbon atoms), 127.87 (2 carbon atoms), 141.45 (2 carbon atoms), 143.80 (2 carbon atoms), 155.95, 178.95. HRMS (ESI): *m/z* $[M+H]^+$ calcd. for $[C_{21}H_{26}NO_4Si]^+$ 384.1626, found: 384.1630. RP-HPLC (220 nm): > 93% (t_R = 13.9 min, *k* = 17.3). $C_{21}H_{25}NO_4Si$ (383.52).

2. Figures S1-S13

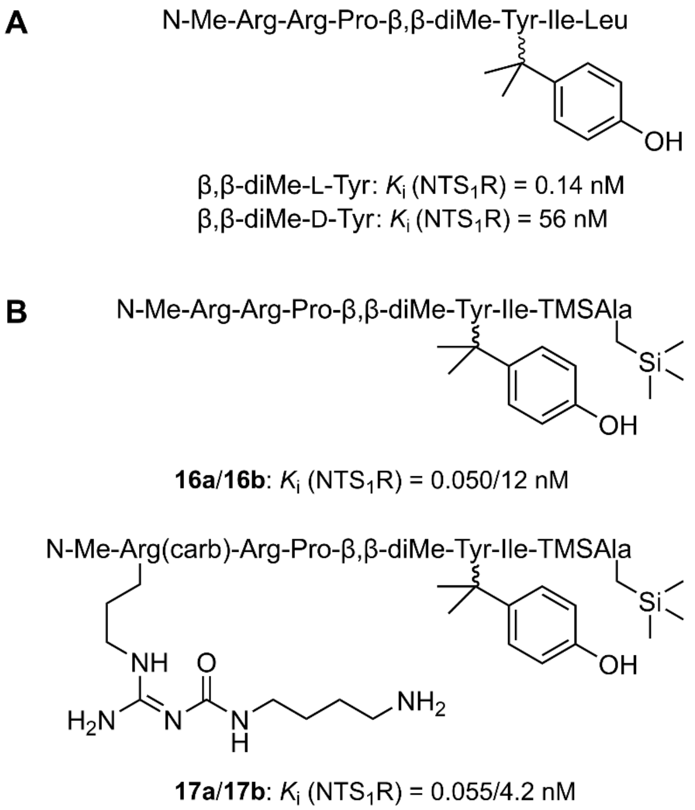

Figure S1. (A) Structures and NTS_1R binding affinities of reported NT(8-13) derivatives containing either *β,β*-dimethly-L-Tyr or *β,β*-dimethly-D-Tyr in position 11 (compound **48** and **49**, respectively, in Schindler et al.[2]). (B) Structures and NTS_1R binding affinities of the diastereomeric pairs **16a**/**16b** and **17a**/**17b**.

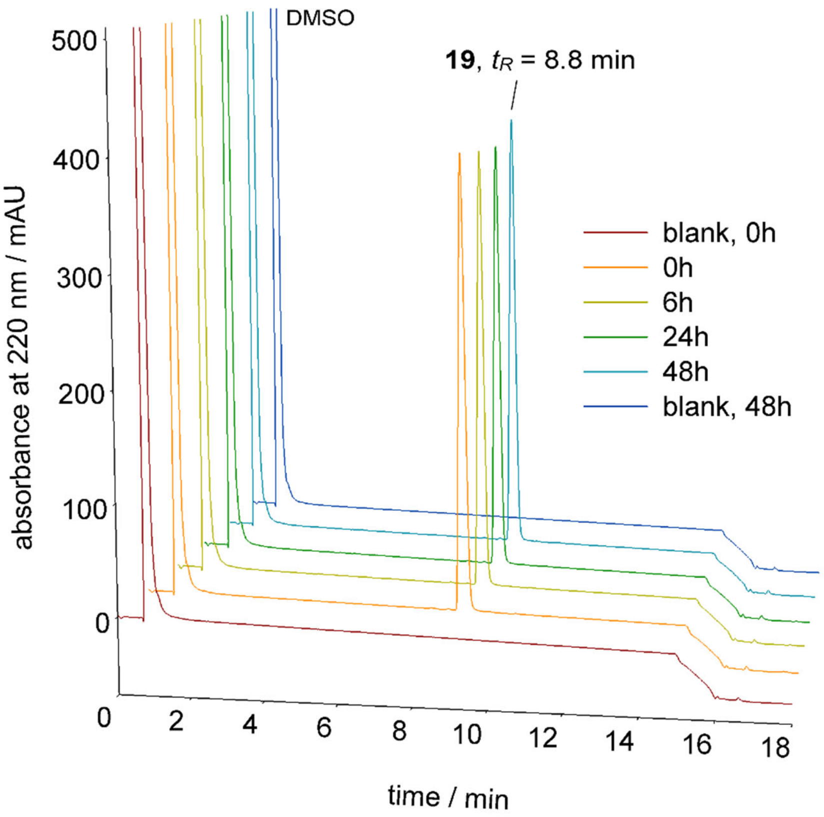

Figure S2. Investigation of the chemical stability of **19** in PBS (pH 7.4). Shown are the chromatograms of the RP-HPLC after incubation for up to 48 hours. **19** showed no decomposition.

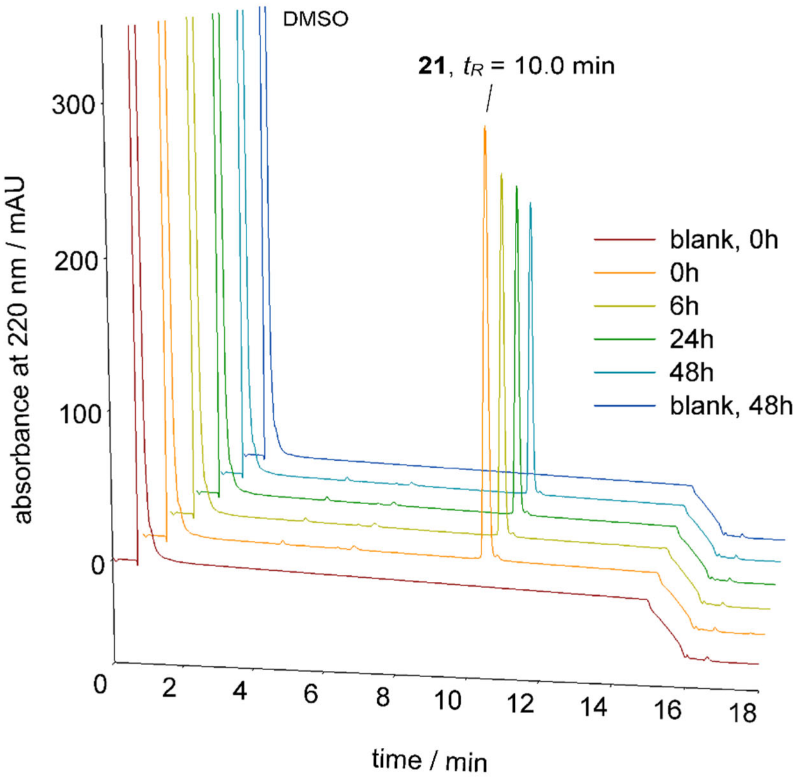

Figure S3. Investigation of the chemical stability of **21** in PBS (pH 7.4). Shown are the chromatograms of the RP-HPLC after incubation for up to 48 hours. **21** showed no decomposition. The slight decrease in peak height over time is caused by adsorption of **21** to the vessel.

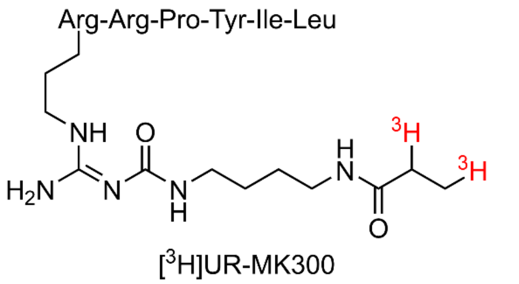

Figure S4. Structure of [^{3}H]UR-MK300 used for the NTS_1R competition binding studies.[3] Note that the tritium atoms in the [^{3}H]propionyl residue do not represent the quantity of tritium isotopes. They only indicate that tritium is present in the respective position.

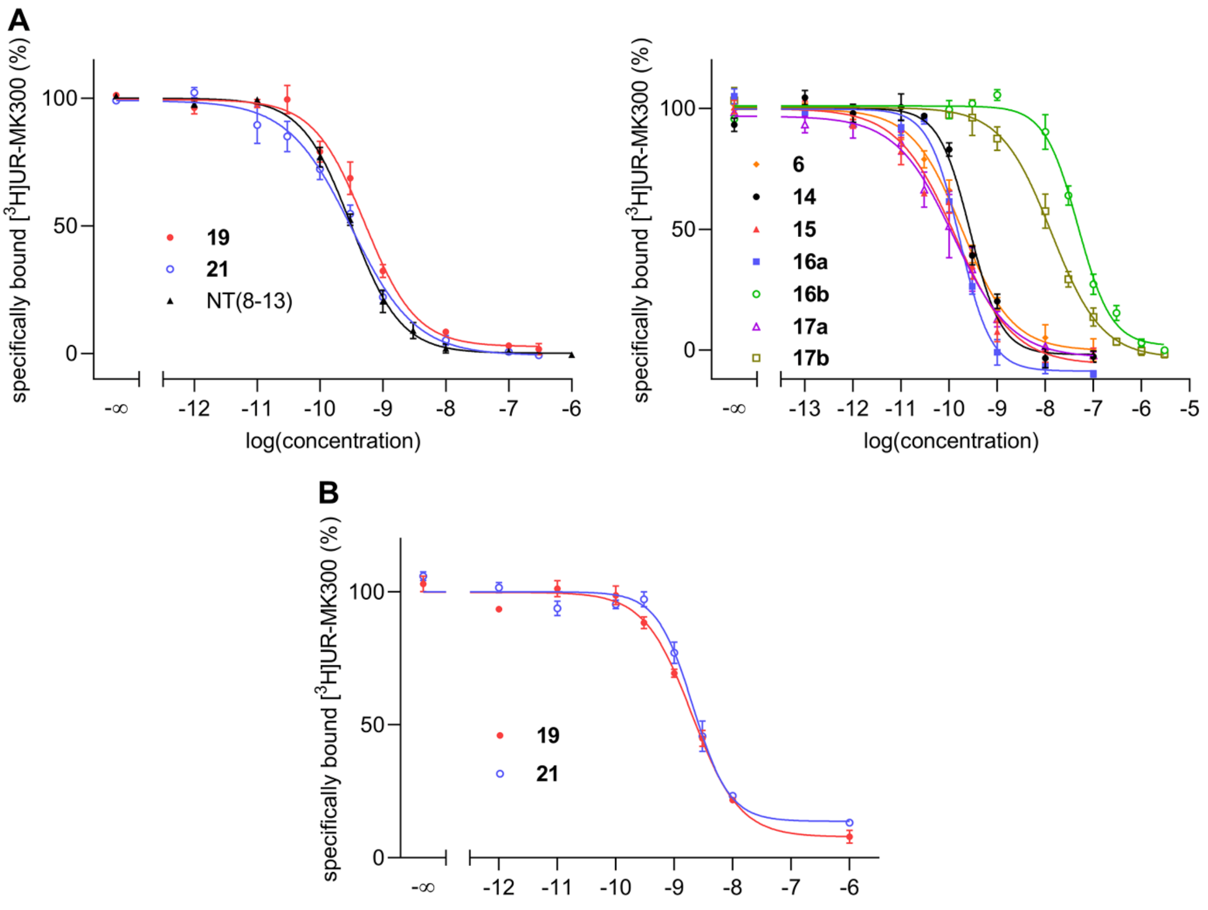

Figure S5. (A) Radioligand displacement curves from competition binding experiments with [^{3}H]UR-MK300 (K_d = 0.41 nM,[2] c = 1 nM) and NT(8-13), **6**, **14-17b**, **19** or **21** at intact HT-29 cells. (B) Radioligand displacement curves from competition binding experiments with [^{3}H]UR-MK300 (K_d = 3.1 nM, c = 5 nM) and **19** or **21** at membranes of HEK293T-hNTS_2R cells. Data represent mean values ± SEM from at least three independent experiments performed in triplicate. All slope factors (data not shown) were not significantly different from -1 (two-tailed t test based on the SEM, $P > 0.05$). For pK_i and K_i values see Table 2 (main article).

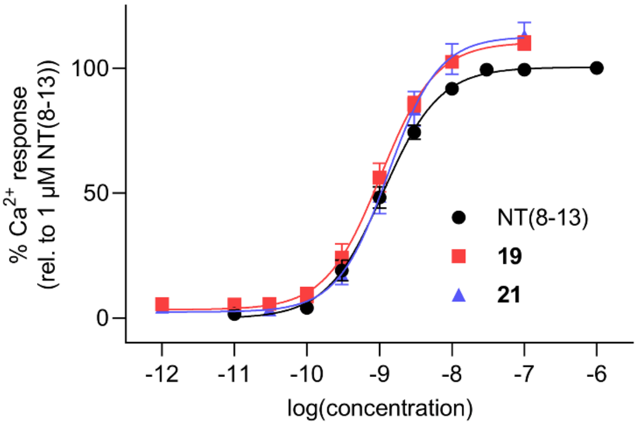

Figure S6. Concentration response curves of NT(8-13), **19**, and **21** obtained from a Fura-2 Ca^{2+} assay performed with HT-29 cells. Mean values ± SEM from at least three independent experiments (performed in triplicate). For pEC_{50} and EC_{50} values see Table 2 (main article).

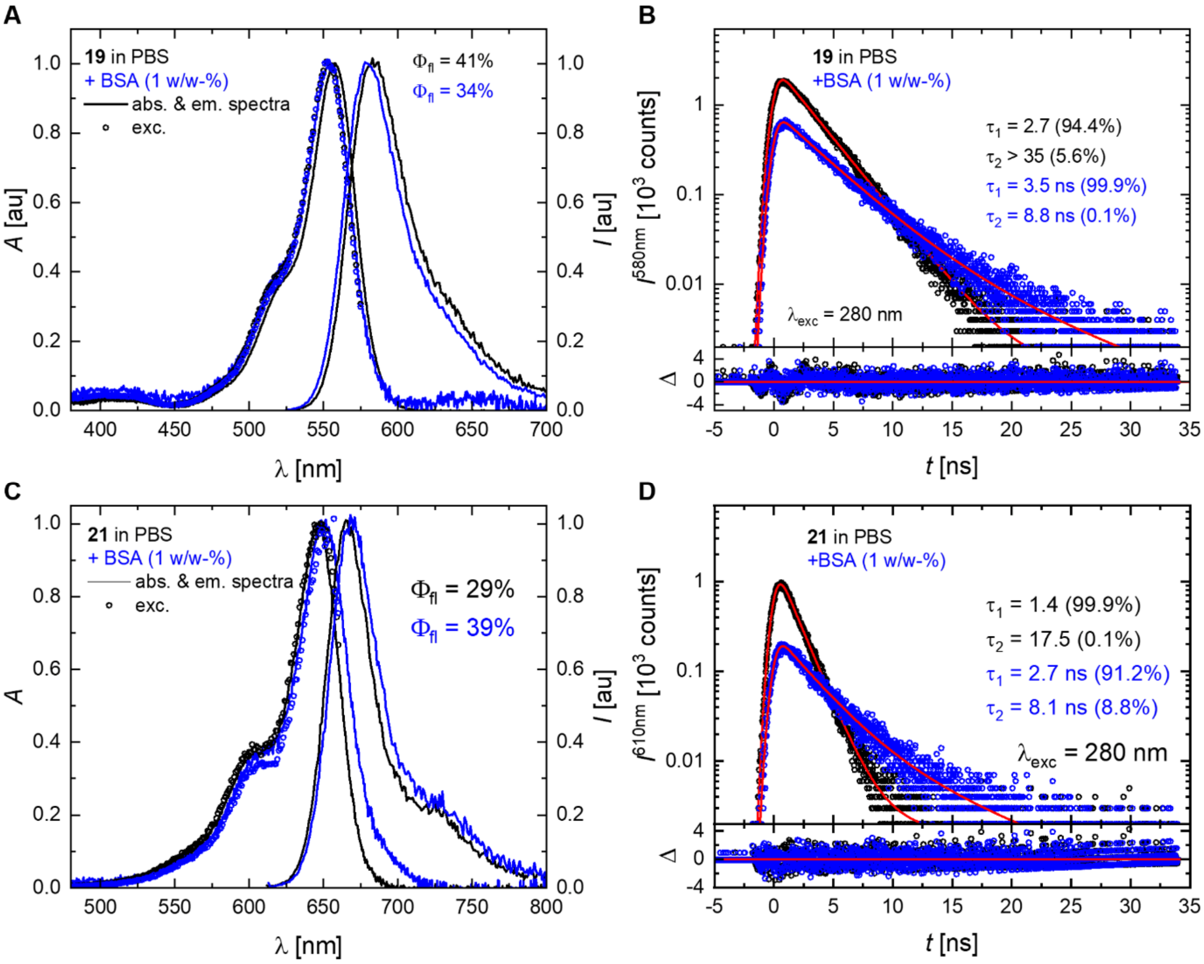

Figure S7. Absorption, emission, and excitation spectra (A, C) as well as emission lifetimes (B, D) of **19** and **21** in PBS in the absence (black) and presence of BSA (1% w/w, blue).

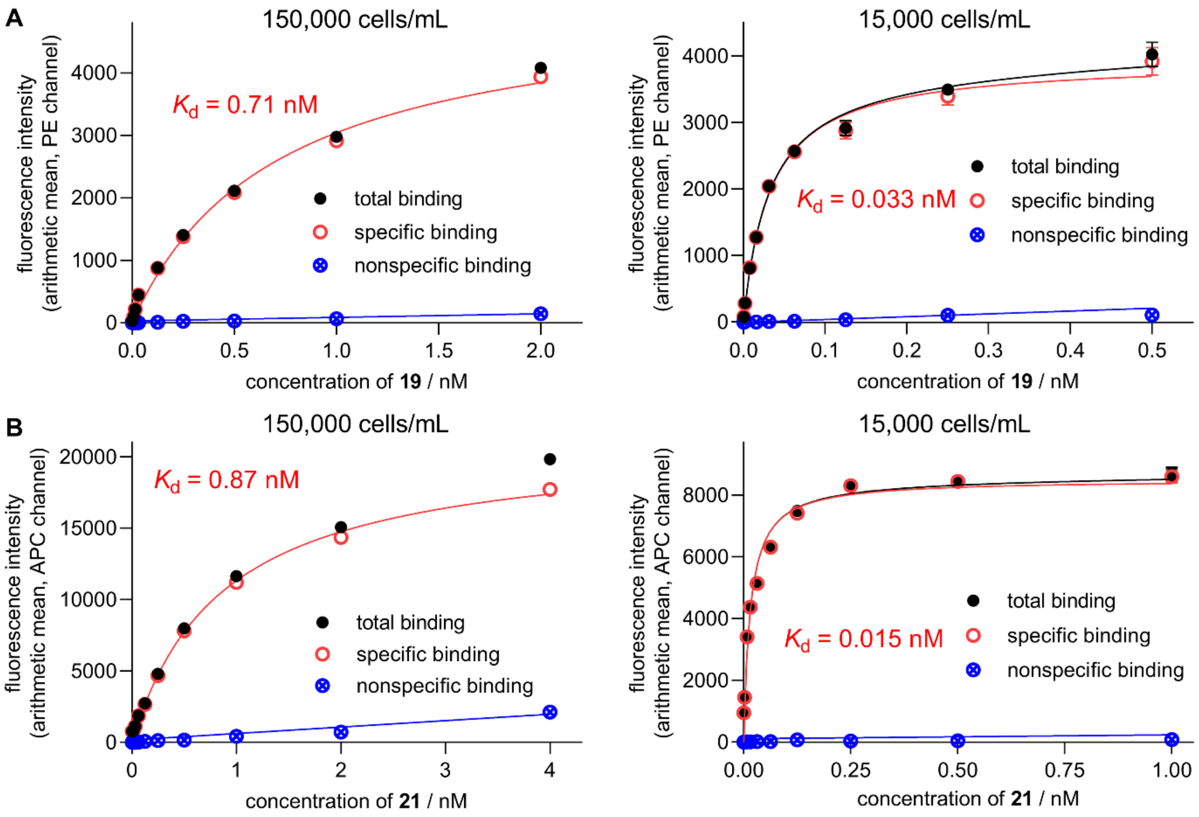

Figure S8. Representative binding isotherms (specific binding, open symbols) of **19** (A) and **21** (B) obtained from flow cytometric saturation binding experiments performed at intact CHO-hNTS$_1$R cells using different cell densities (150,000 or 15,000 cells/mL) (incubation: 120 min at 23 °C). Nonspecific binding was determined in the presence of 1 µM NT(8-13). K_d values are presented in Table 4 (main article). Data represent mean values ± SEM (total and nonspecific binding) or calculated values ± propagated error (specific binding) from representative experiments performed in triplicate.

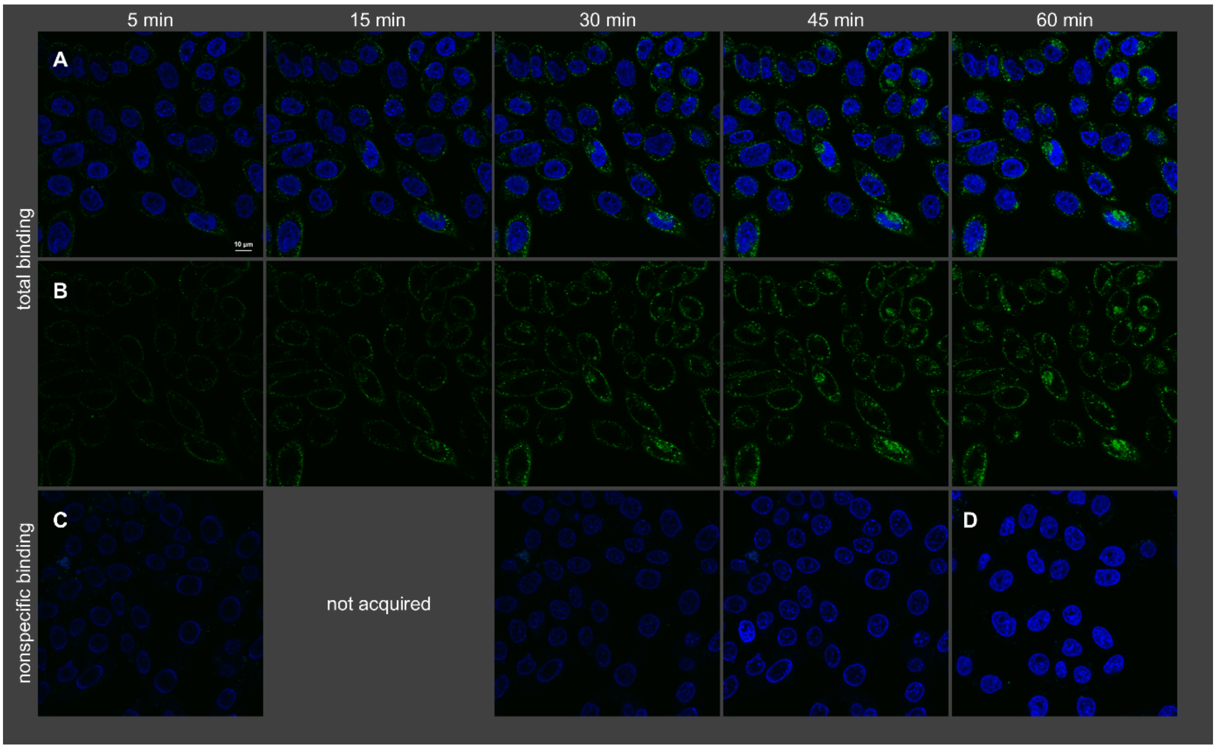

Figure S9. Visualization of binding of **19** (2 nM) to intact CHO-hNTS$_1$R cells (temperature: 22 °C) by confocal microscopy. Shown is total binding (A, B), nonspecific binding (C), and autofluorescence (D). Nuclei were stained with H33342 (2 µg/mL). (A) Merged fluorescence of **19** (green) and nuclei (blue). (B) Fluorescence of **19**, without nuclei. (C) Merged fluorescence of **19** and nuclei acquired in the presence of 1 µM NT(8-13).

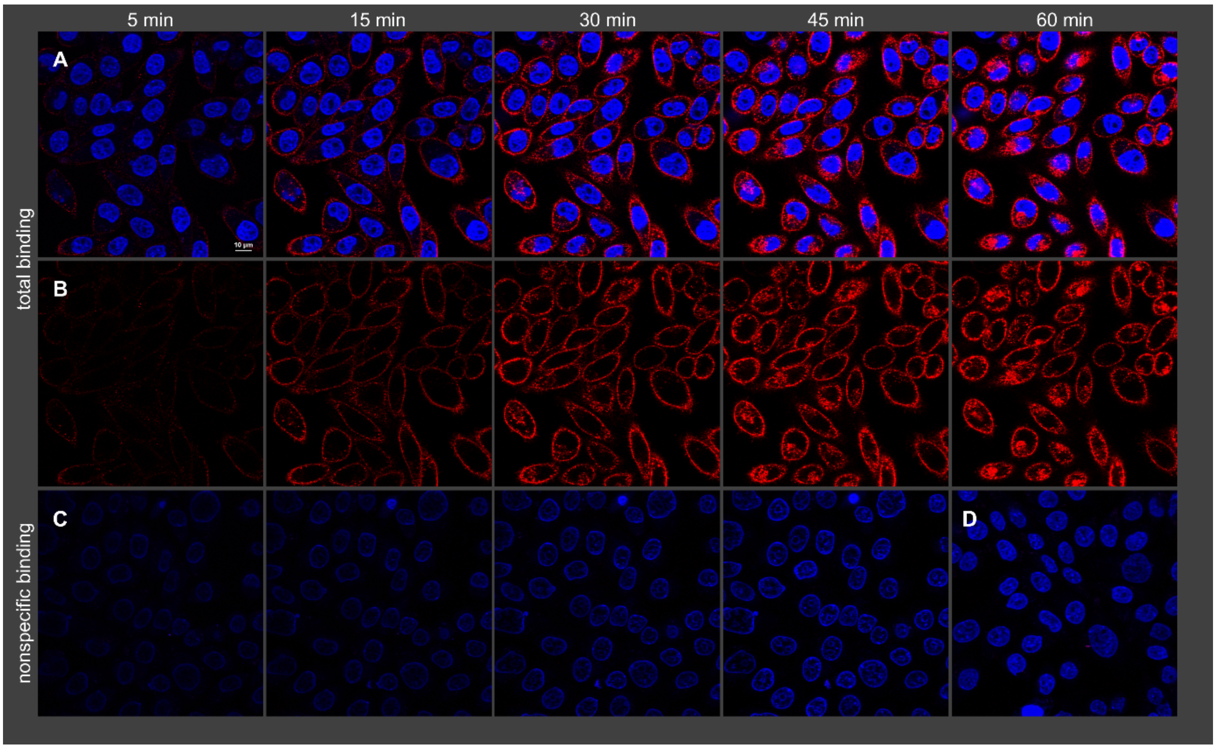

Figure S10. Visualization of binding of **21** (2 nM) to intact CHO-hNTS$_1$R cells (temperature: 22 °C) by confocal microscopy. Shown is total binding (A, B), nonspecific binding (C), and autofluorescence (D). Nuclei were stained with H33342 (2 µg/mL). (A) Merged fluorescence of **21** (red) and nuclei (blue). (B) Fluorescence of **21**, without nuclei. (C) Merged fluorescence of **21** and nuclei acquired in the presence of 1 µM NT(8-13).

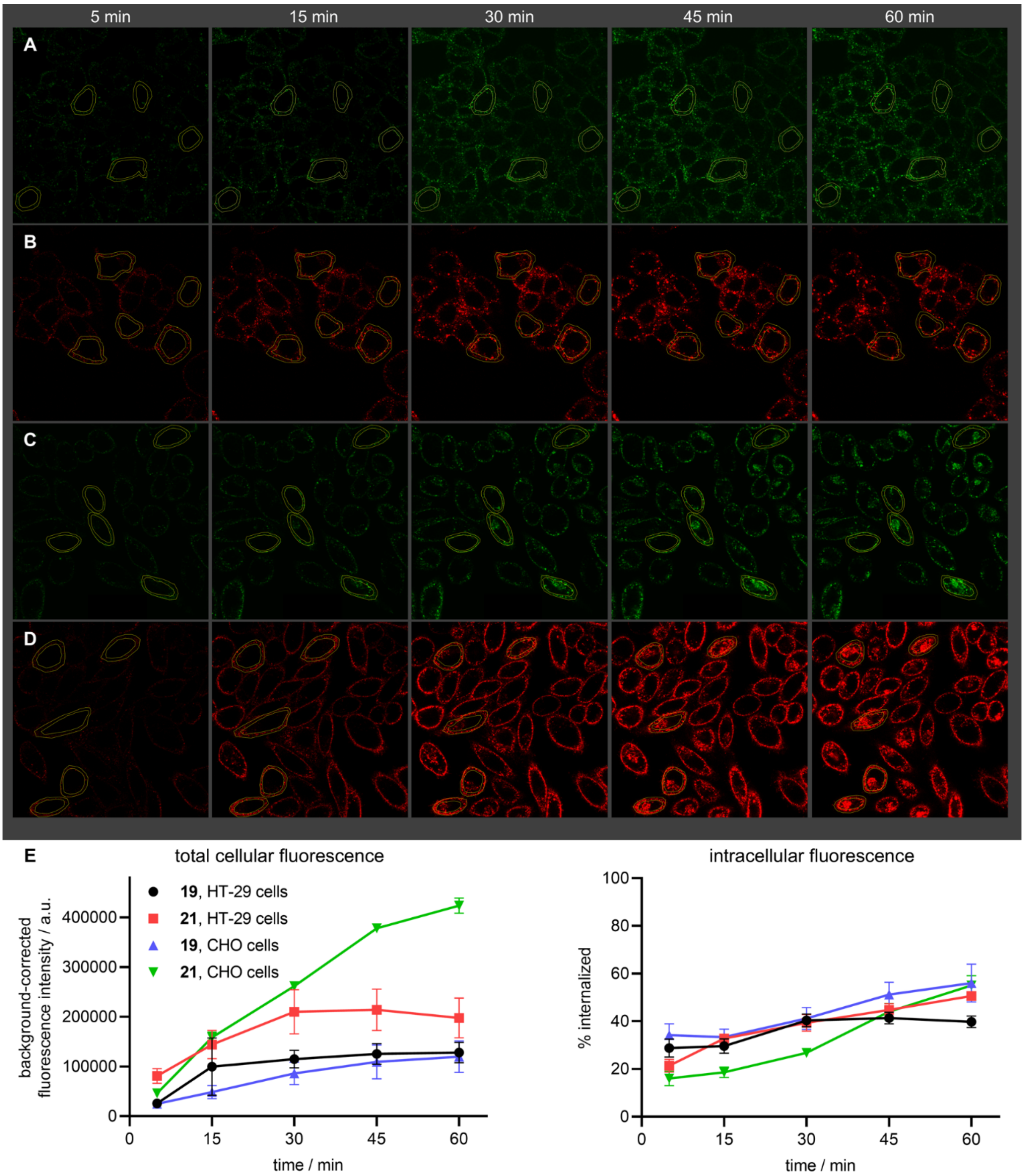

Figure S11. Estimation of total cellular fluorescence and intracellular fluorescence based on the confocal microscopy images shown in Figure 6 (**19**, HT-29 cells; main article) (A), Figure 7 (**21**, HT-29 cells; main article) (B), Figure S9 (**19**, CHO cells) (C), and Figure S10 (**21**, CHO cells) (D). For each image series, two regions of interest (ROI) were defined for five individual cells (yellow line). The outer ROI includes the entire cell and the inner ROI represents the inside of the cell. The ROIs were visually defined based on the 5 min time point. (E) Graphical presentation of the total cellular fluorescence (outer ROIs, mean values ± SEM) and the intracellular fluorescence (inner ROIs, mean values ± SEM), representing internalized fluorescent ligand, calculated as % of total cellular fluorescence. Fluorescence densities were determined with ImageJ.[4] Fluorescence densities were background-corrected according to: *corrected fluorescence density (inner or outer ROI) = fluorescence density of the ROI defining total or intracellular fluorescence* – (*background mean fluorescence of the background ROI* × *area of the ROI defining total or intracellular fluorescence*). Background ROIs are not depicted. Note that the intensities of the total fluorescence can not be directly compared for the two cell lines since different settings (laser power, detector gain) were used.

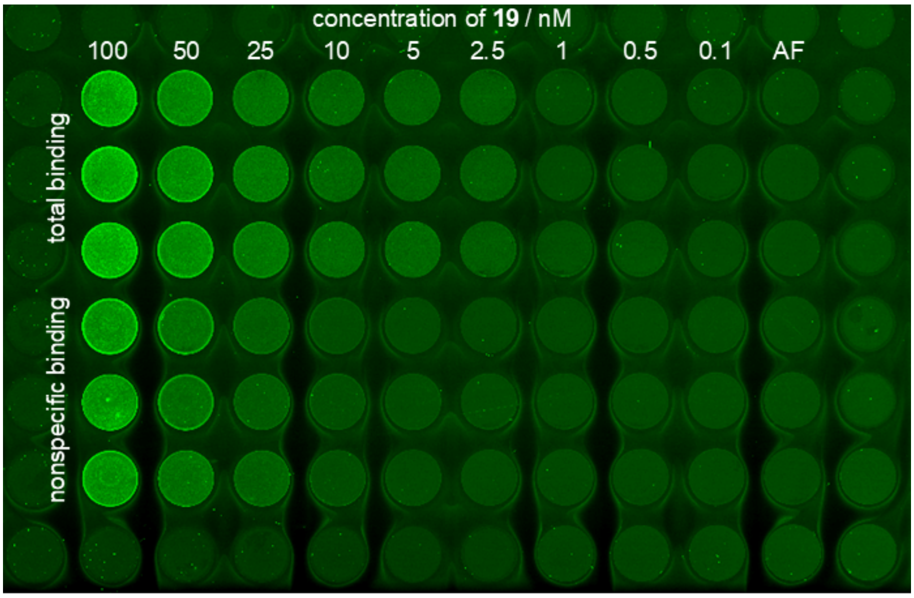

Figure S12. Fluorescence image of a 96-well plate with adherent HT-29 cells acquired with an Azure Sapphire Biomolecular Imager (laser: 520 nm; pixel size: 20 µm) 2 h after incubation with **19** at rt. Nonspecific binding was determined in the presence of SR142948 (1 µM). Shortly before image acquisition, cells were washed three times with cold buffer.

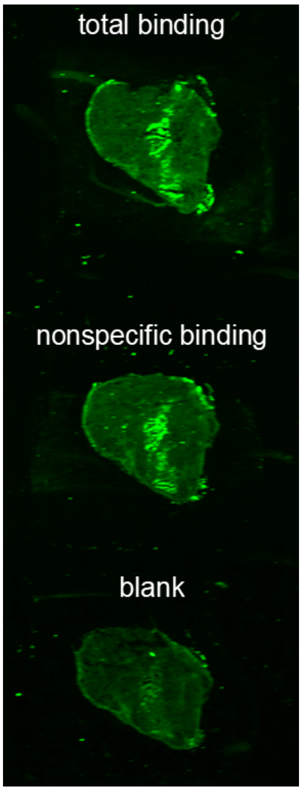

Figure S13. Fluorescence images of cryosections (size: 25-28 mm^2, thickness: 10 µm) of a HT-29 tumor (the same tumor was used for experiments with **21**; see tumor 2 in Figure 8, main article) acquired after incubation with **19** (10 nM) at rt for 60 min with an Azure Sapphire FL Biomolecular Imager (laser: 520 nm; pixel size: 20 µm). Nonspecific binding was determined in the presence of SR142948 (5 µM). Blank samples were incubated with neat buffer to determine background signals. Shortly before image acquisition, tumor sections were washed three times with cold buffer.

3. Table S1

Table S1. Recoveries of NT(8-13), **15**, **17a**, **19**, and **21** from human plasma/PBS 1:2 v/v and ratios of compound-recovery over recovery of internal standard (1-methyl-D-tryptophan).

compd.	recovery compd. (%)[a]	recovery 1-methyl-D-tryptophan (%)[a]	ratio[b]
NT(8-13)	100	101	1.00
	91	97	0.93
	92	90	1.03
	96	94	1.01
	92	89	1.03
			(1.00 ± 0.02)
**15**	95	94	1.00
	91	93	0.99
	87	86	1.01
	96	97	0.99
	90	91	0.99
			(1.00 ± 0.004)
**17a**	50	50	1.00
	84	81	1.04
	76	84	0.91
	76	78	0.97
	94	94	1.01
			(0.99 ± 0.02)
**19**	97	93	1.05
	110	109	1.02
	104	106	0.99
	117	110	1.06
			(1.03 ± 0.02)
**21**	101	102	0.99
	77	69	1.12
	93	87	1.07
			(1.06 ± 0.04)

[a]Recoveries of the peptides and of the internal standard from human plasma/PBS 1:2 v/v using a peptide concentration of 40 μM and an internal standard concentration of 10 μM (four independent experiments). [b]Ratios of peptide recovery over recovery of internal standard calculated for individual experiments, as well as mean recovery ratios ± SEM (given in parenthesis).

4. Electropherograms of the CE analysis of (*R*,*S*)-10 and (*R*)-10

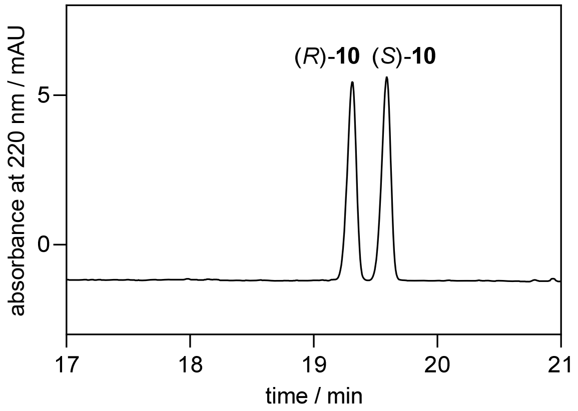

Section of the electropherogram of the CE analysis of (*R*,*S*)-**10** showing the separated signals of the enantiomers

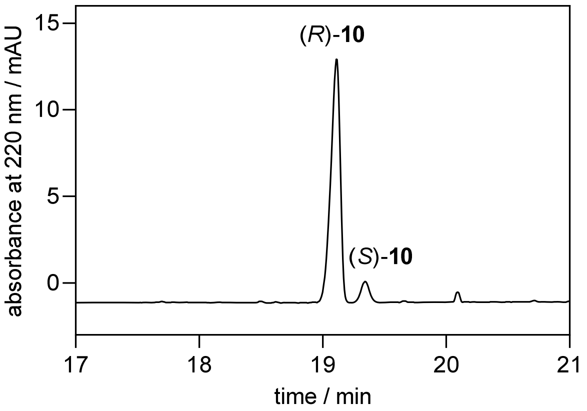

Section of the electropherogram of the CE analysis of (*R*)-**10** showing the signal of (*R*)-**10** and the minor enantiomer (*S*)-**10** (7%)

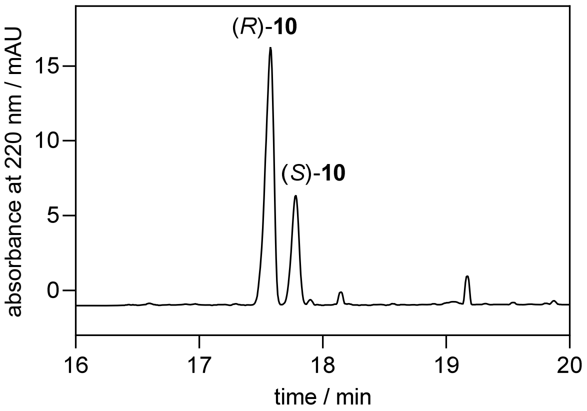

Section of the electropherogram of the CE analysis of (*R*)-**10** spiked with (*R*,*S*)-**10** showing the signals of (*R*)-**10** and (*S*)-**10**

5. RP-HPLC chromatograms of 6, 10, 14-17b, 19, and 21 (purity controls)

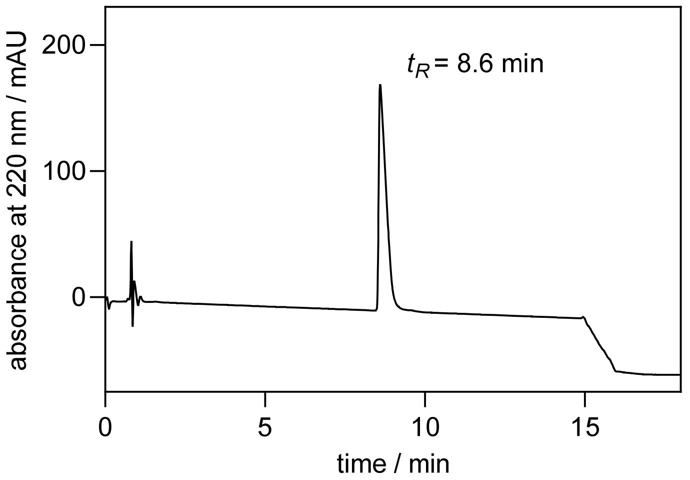

RP-HPLC analysis of compound **6**

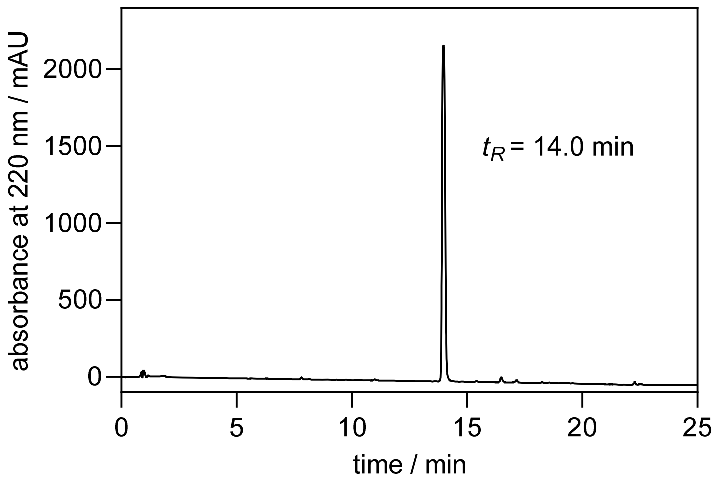

RP-HPLC analysis of compound **10**

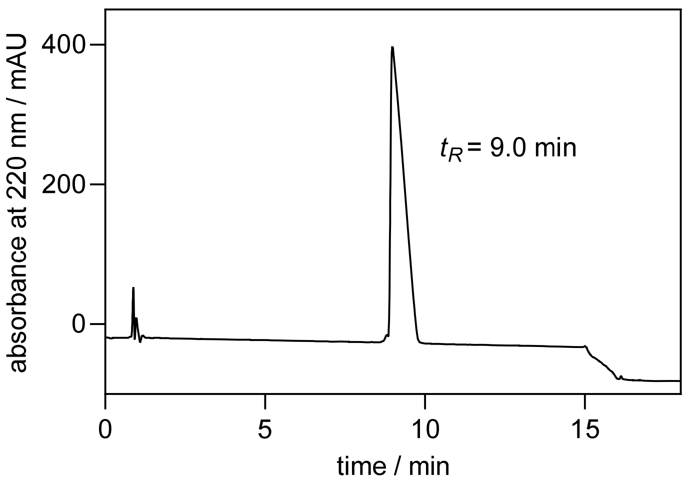

RP-HPLC analysis of compound **14**

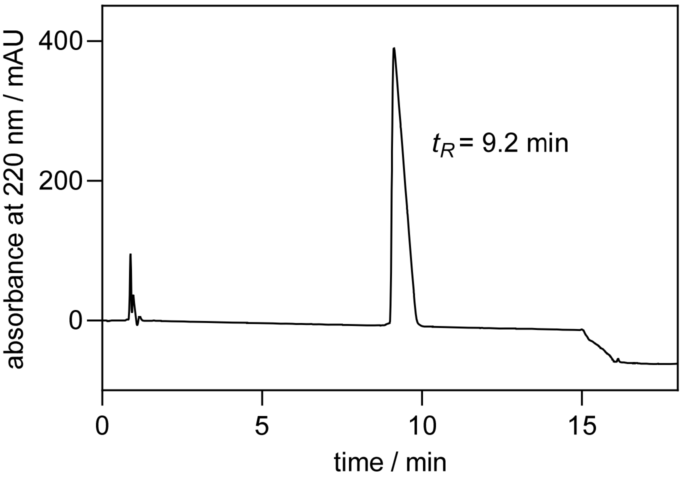

RP-HPLC analysis of compound **15**

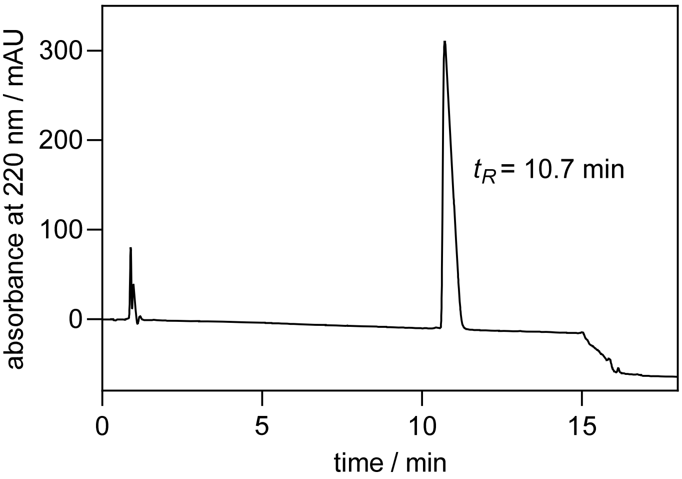

RP-HPLC analysis of compound **16a**

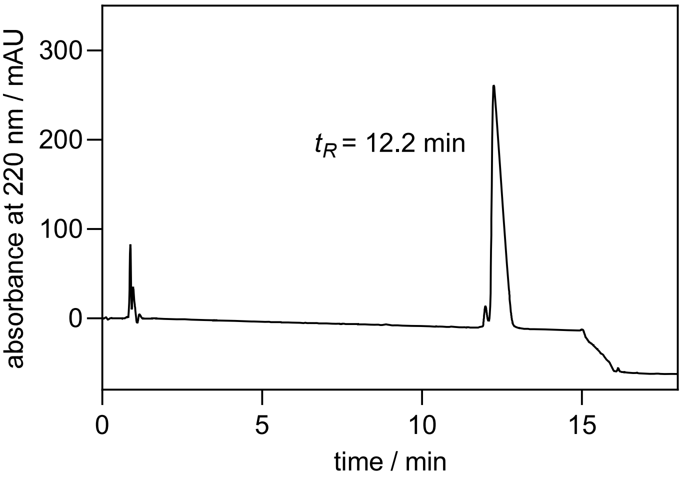

RP-HPLC analysis of compound **16b**

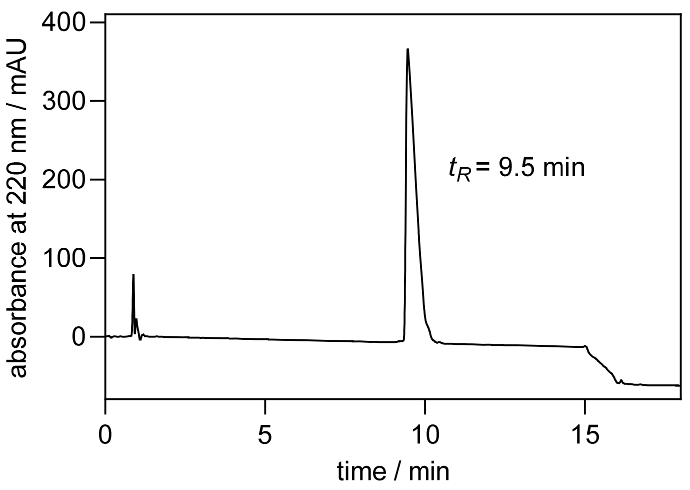

RP-HPLC analysis of compound **17a**

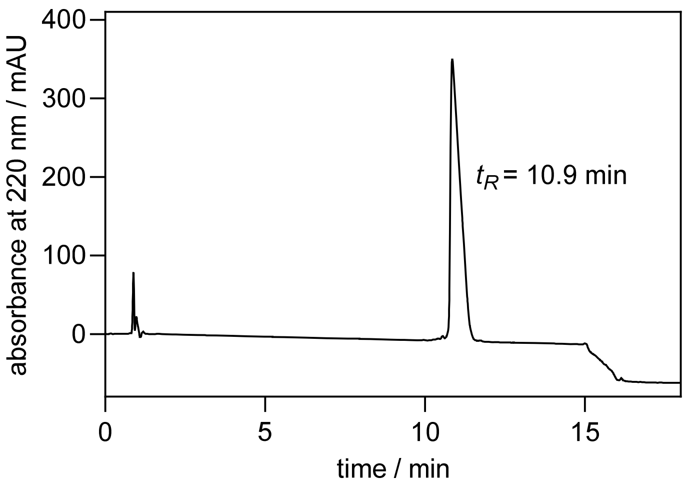

RP-HPLC analysis of compound **17b**

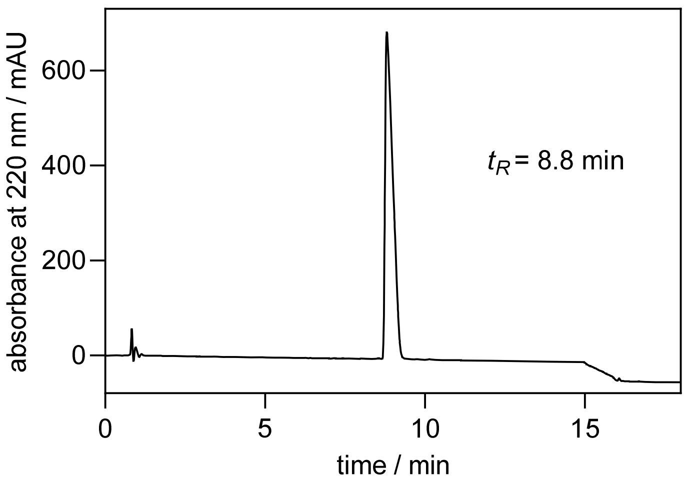

RP-HPLC analysis of compound **19**

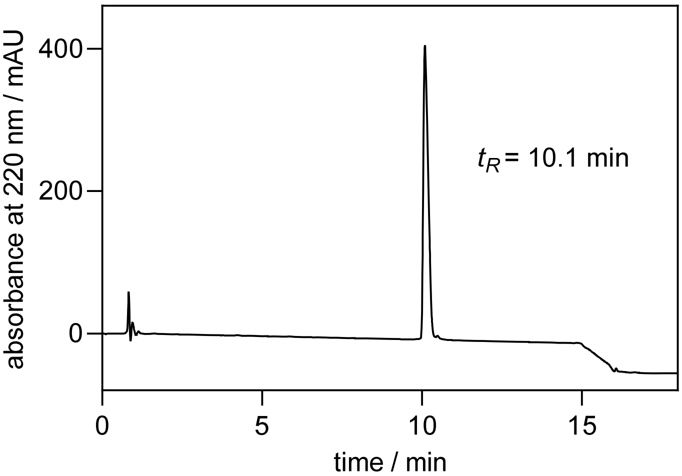

RP-HPLC analysis of compound **21**

6. ^{1}H-NMR spectrum and ^{13}C-NMR spectrum of compound 10 in $CDCl_3$

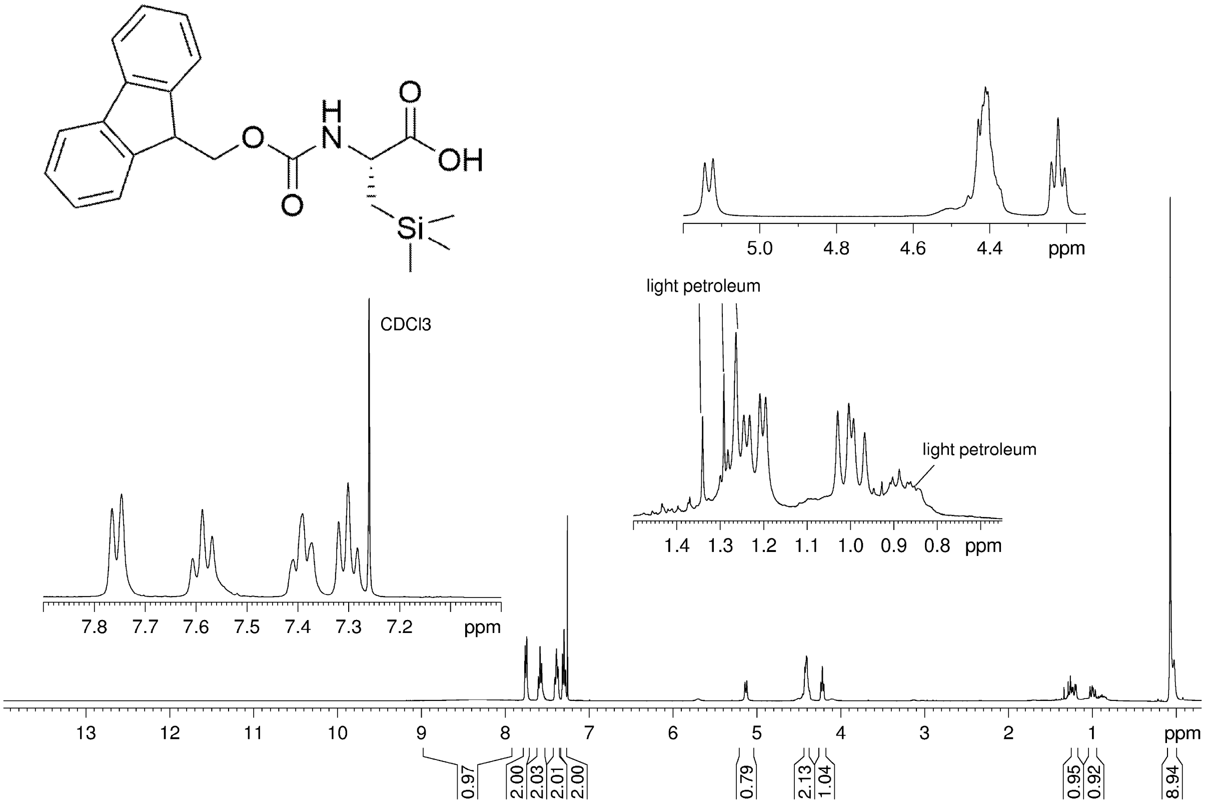

^{1}H-NMR spectrum (400 MHz, $CDCl_3$) of compound **10**

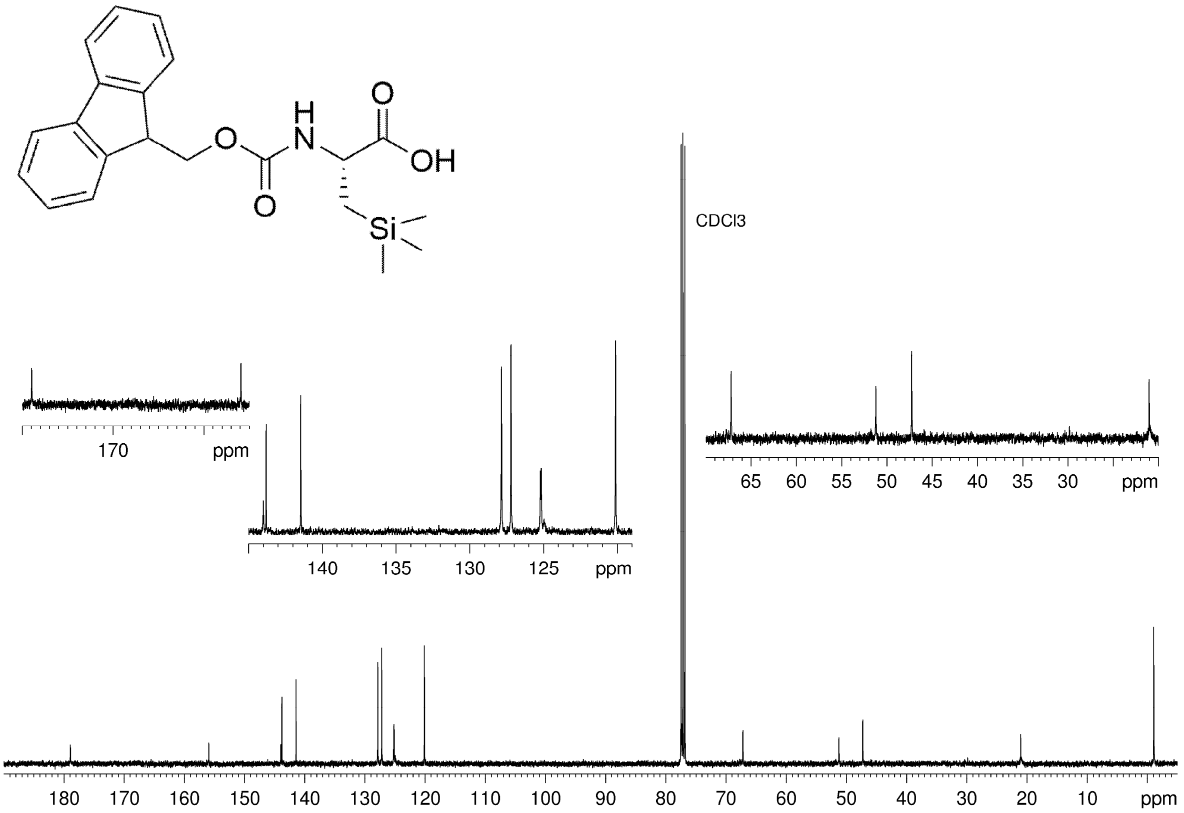

^{13}C-NMR spectrum (100 MHz, $CDCl_3$) of compound **10**

7. ^{1}H-NMR spectra of compounds 14-17b, 19, and 21 and ^{13}C-NMR spectra of compounds 14-17b in DMSO-d$_6$ and DMSO-d$_6$/D$_2$O 4:1 v/v

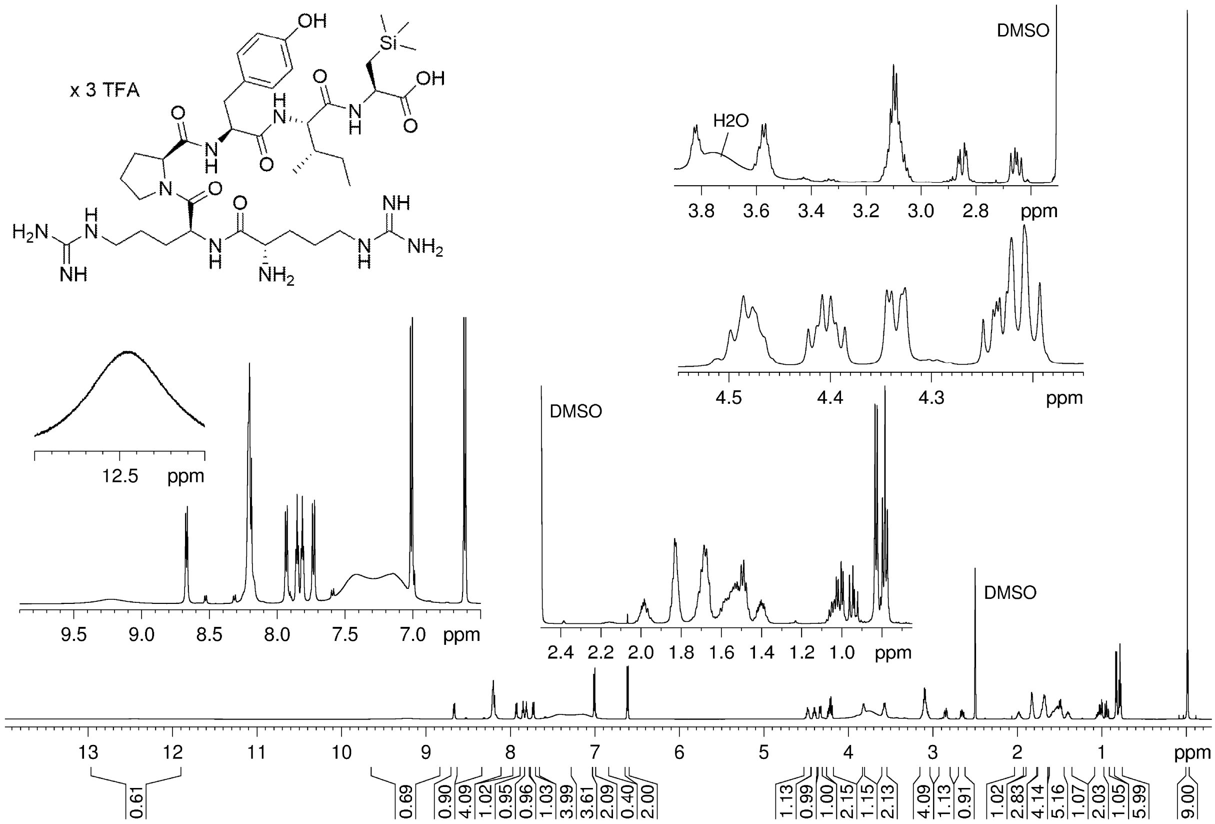

^{1}H-NMR spectrum (600 MHz, DMSO-d$_6$) of compound **14**

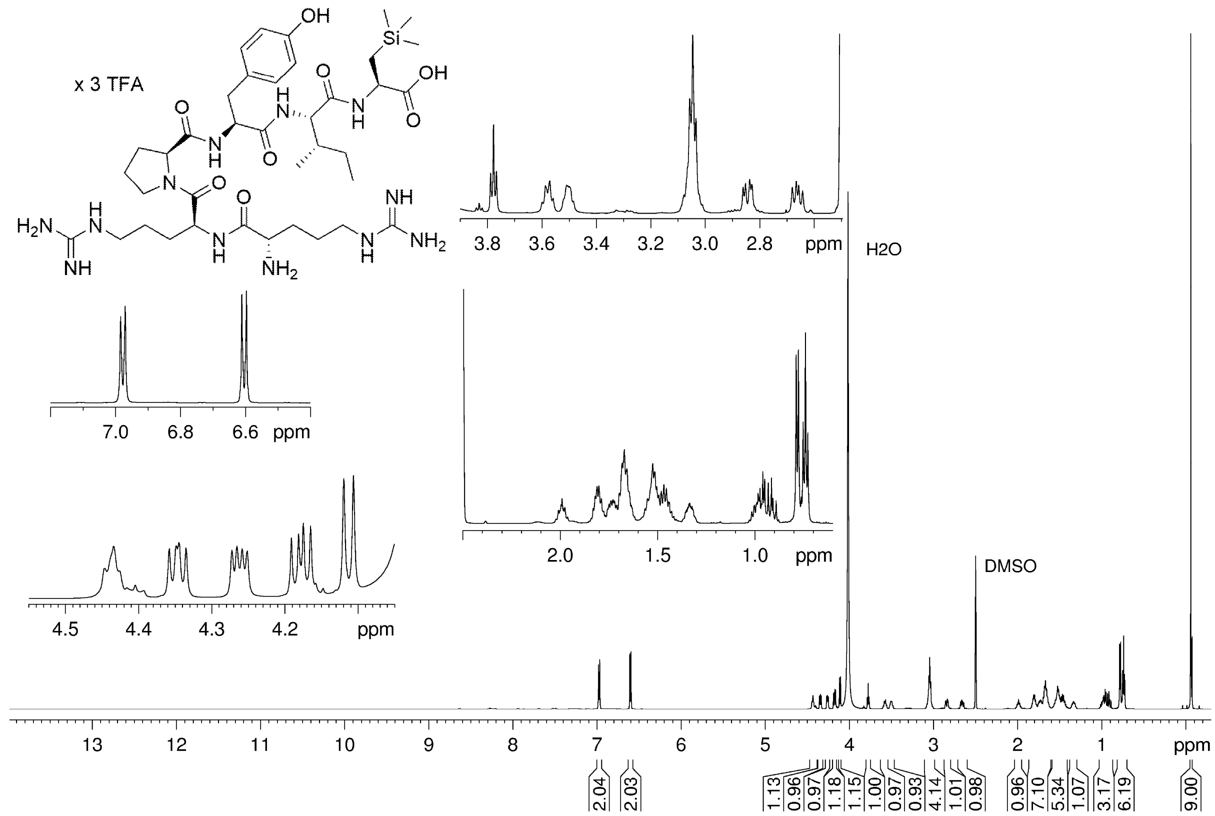

^{1}H-NMR spectrum (600 MHz, DMSO-d$_6$/D$_2$O 4:1 v/v) of compound **14**

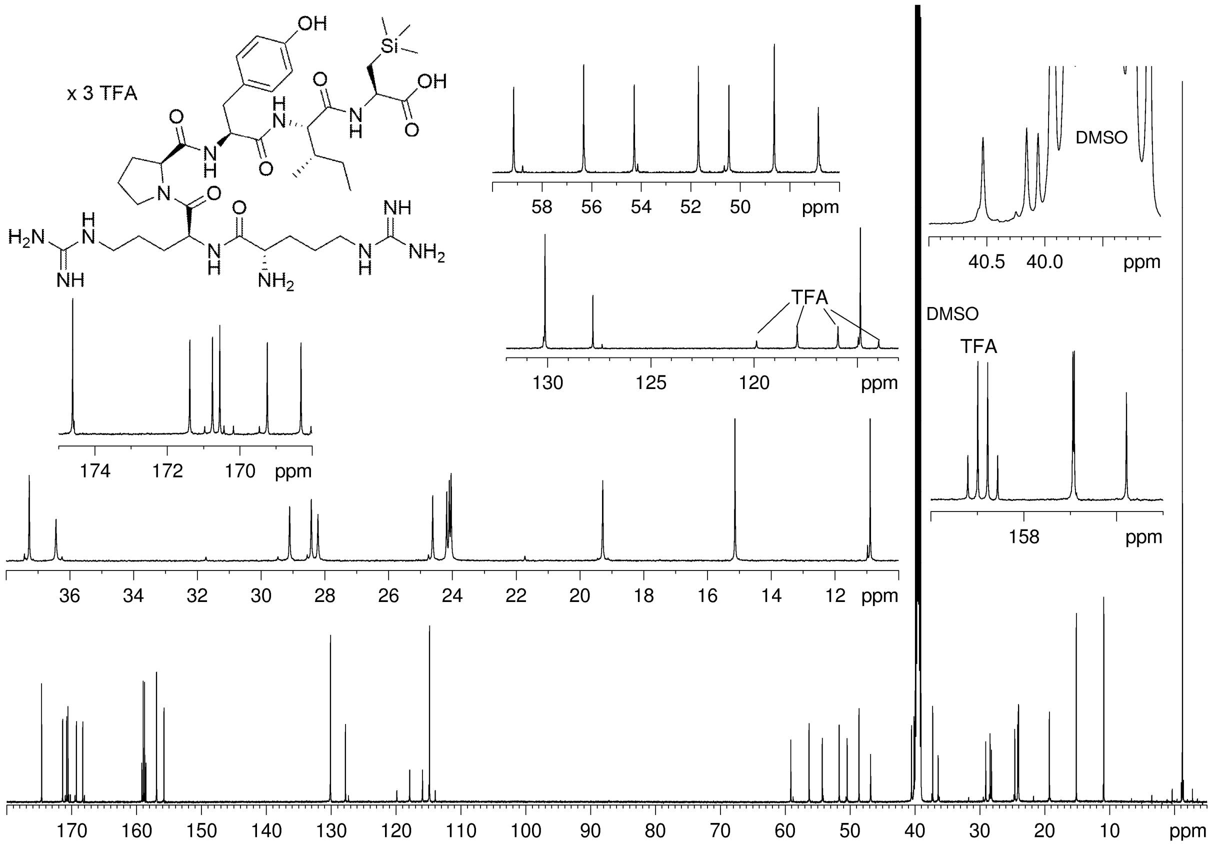

^{13}C-NMR spectrum (150 MHz, DMSO-d$_6$) of compound **14**

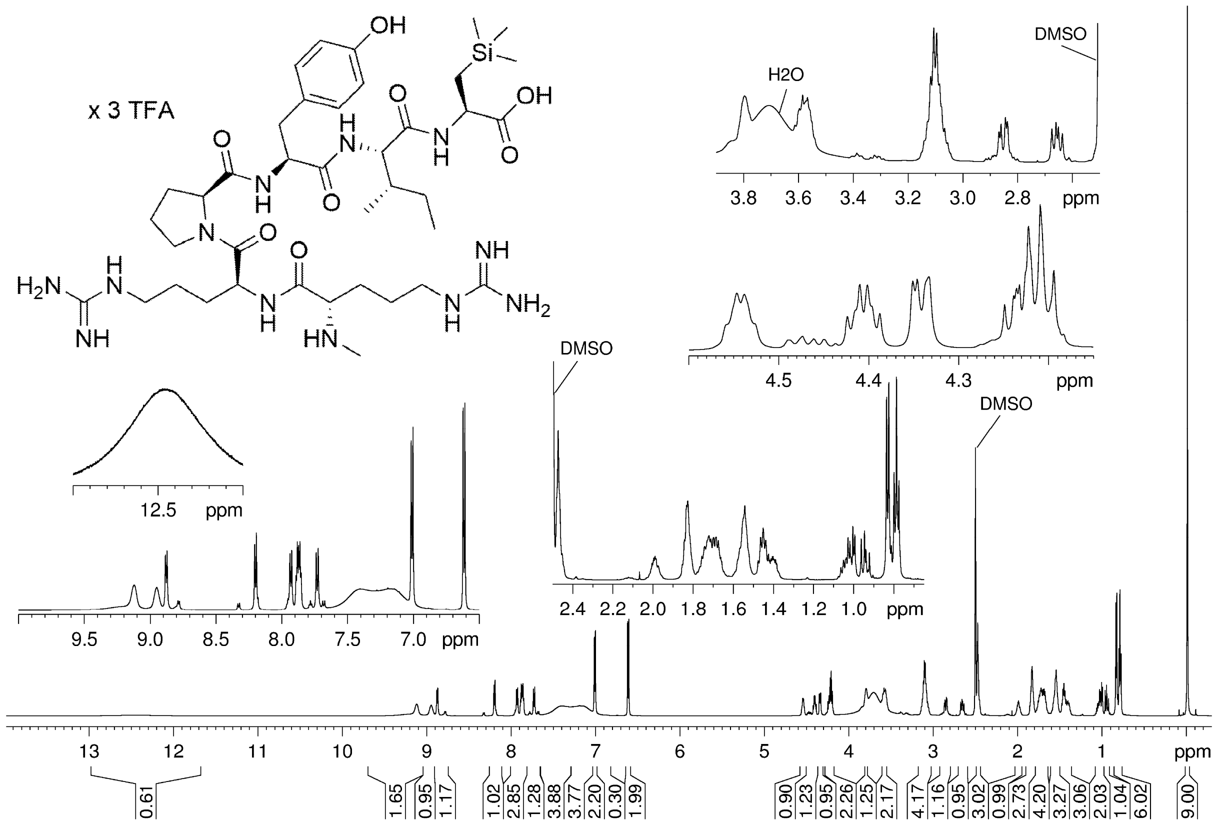

^{1}H-NMR spectrum (600 MHz, DMSO-d$_6$) of compound **15**

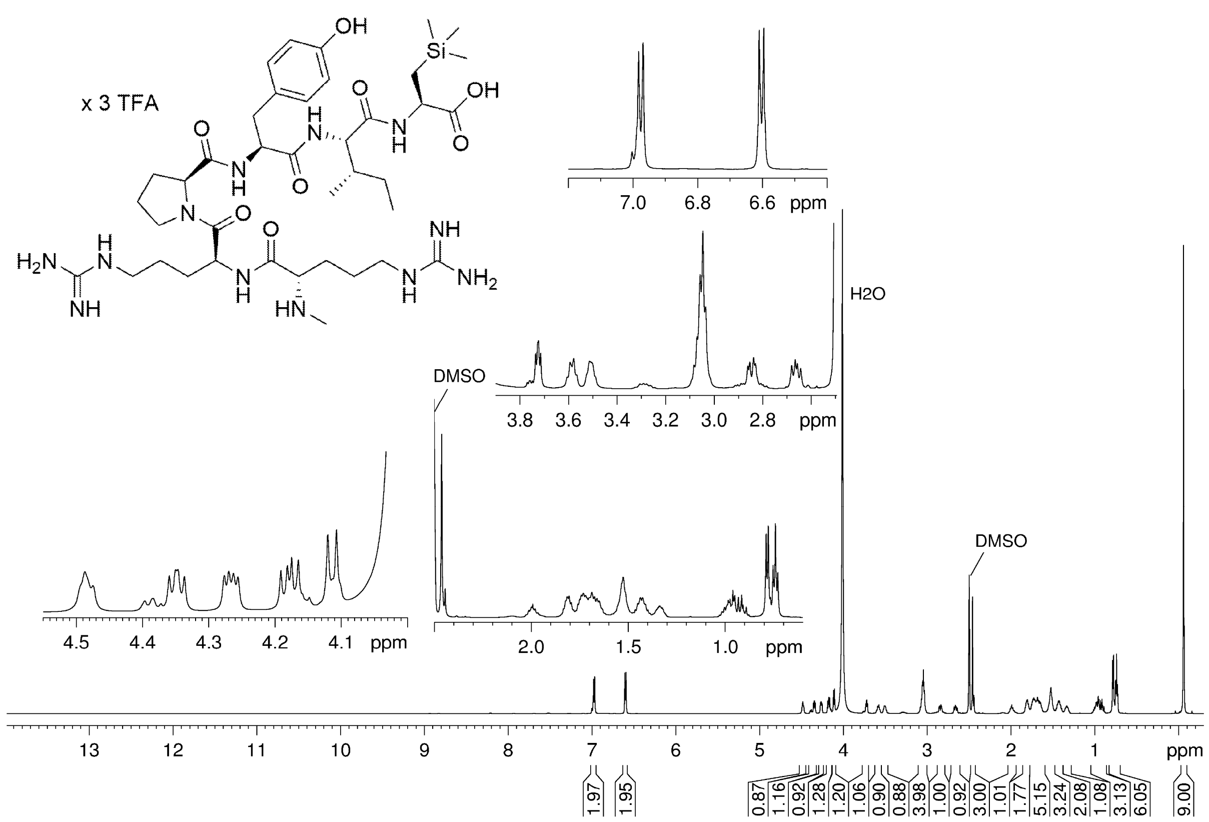

^{1}H-NMR spectrum (600 MHz, DMSO-d$_6$/D$_2$O 4:1 v/v) of compound **15**

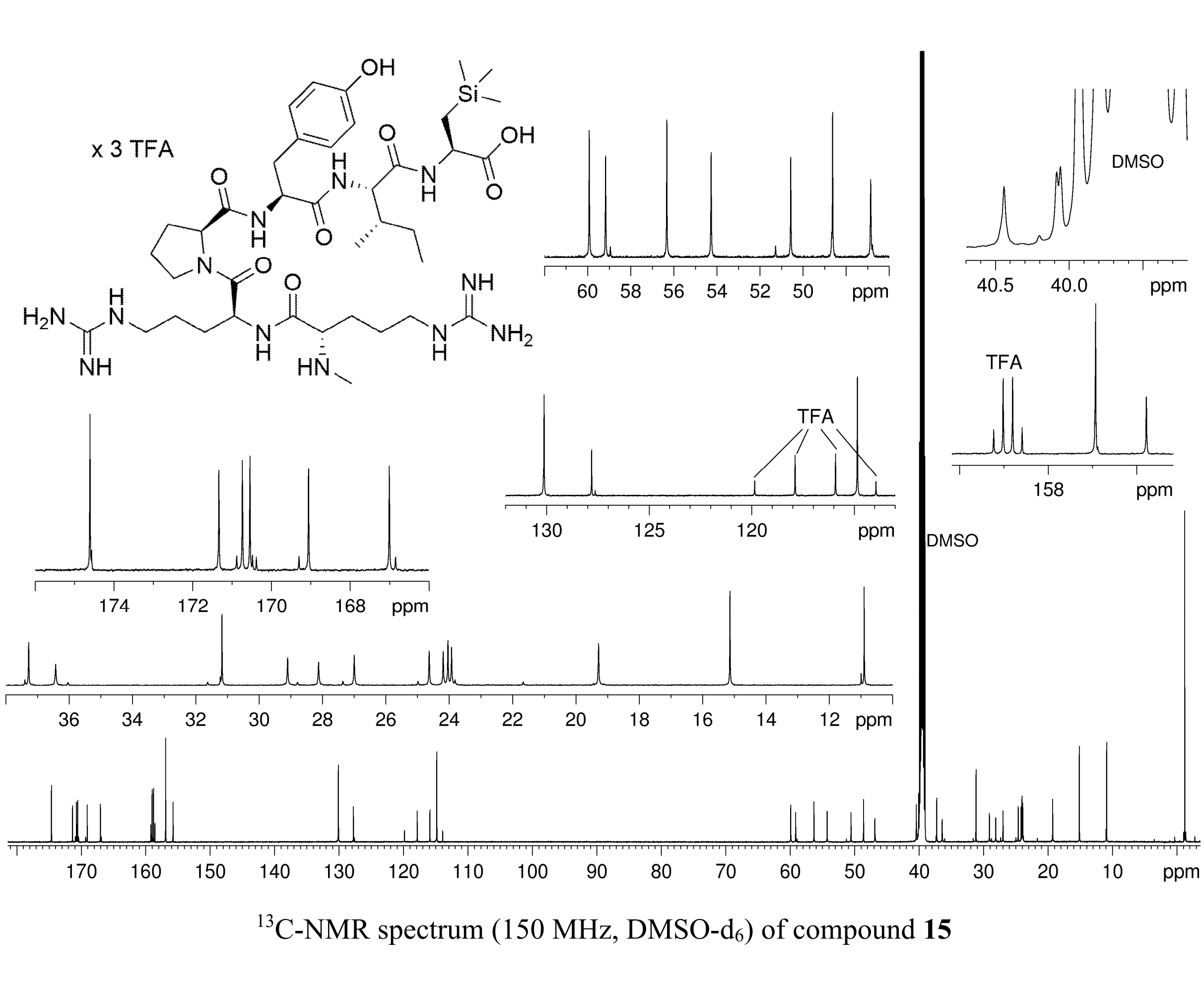

^{13}C-NMR spectrum (150 MHz, DMSO-d$_6$) of compound **15**

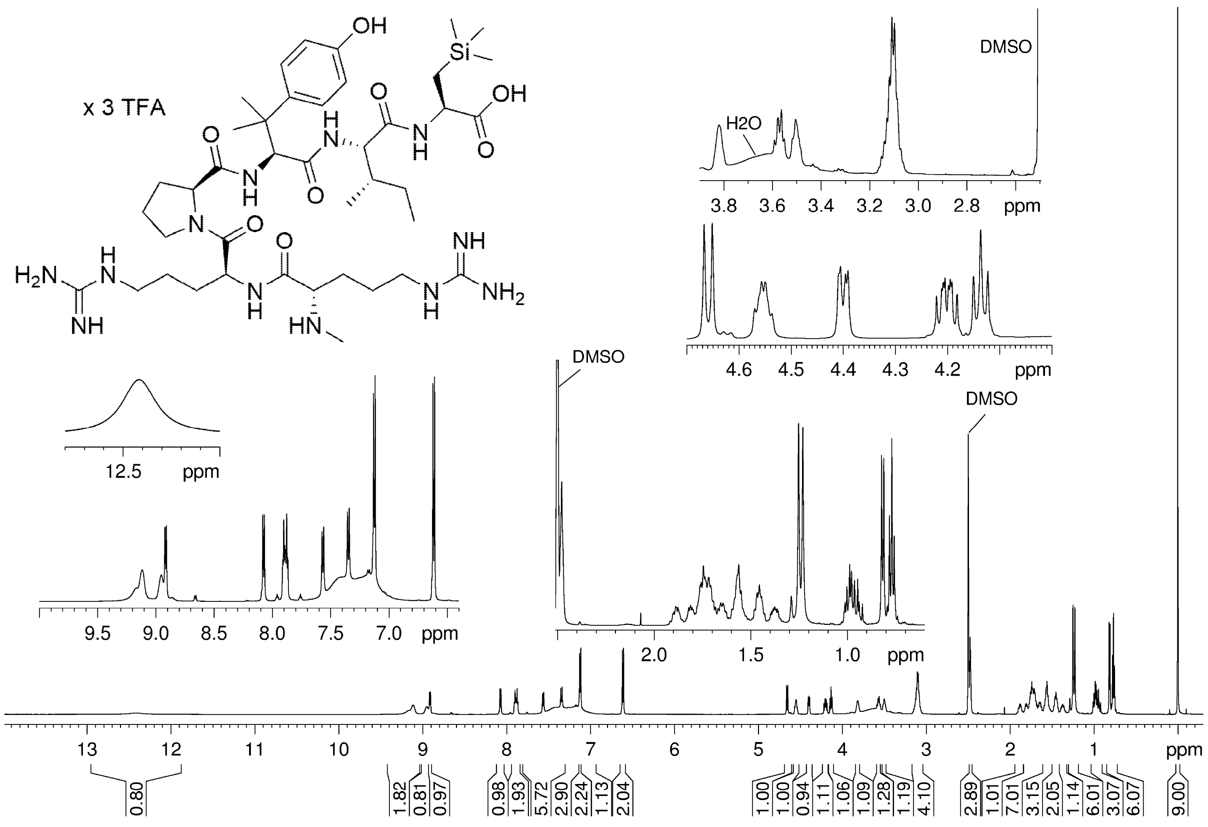

^{1}H-NMR spectrum (600 MHz, DMSO-d$_6$) of compound **16a**

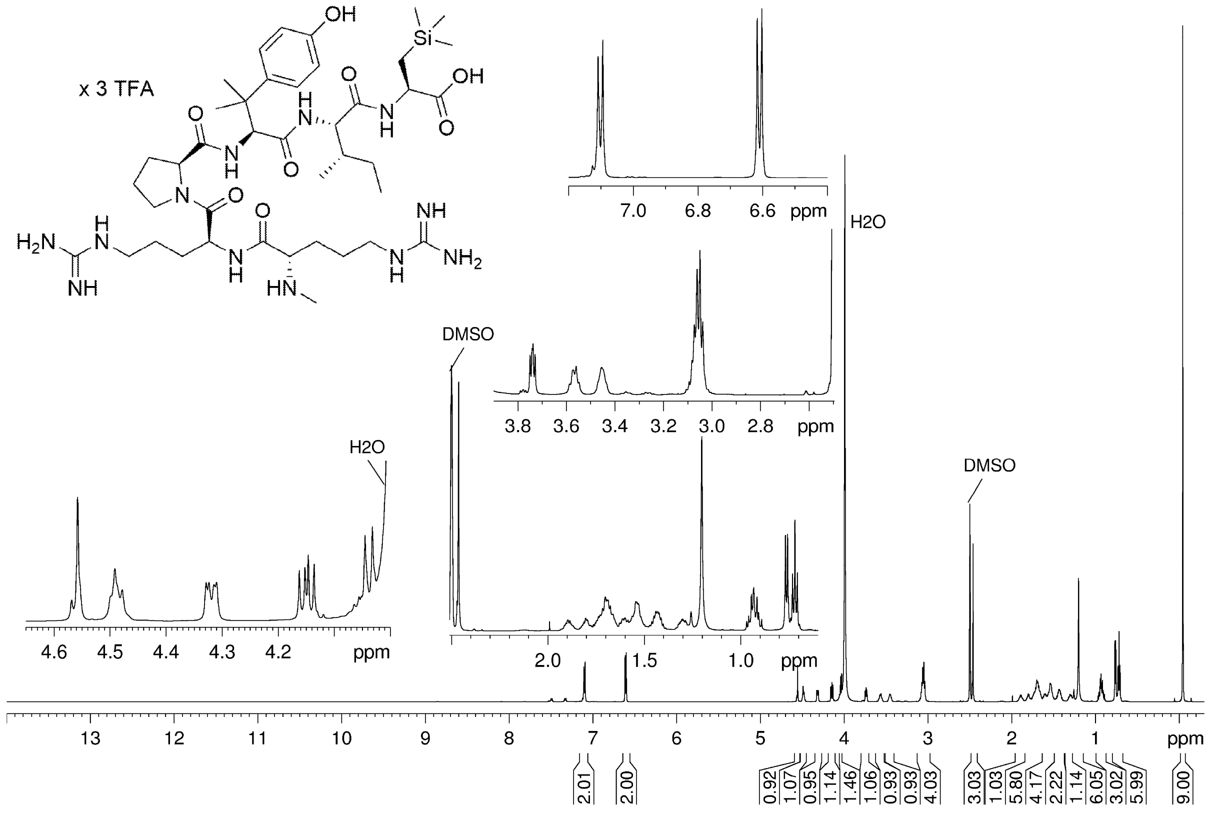

^{1}H-NMR spectrum (600 MHz, DMSO-d$_6$/D$_2$O 4:1 v/v) of compound **16a**

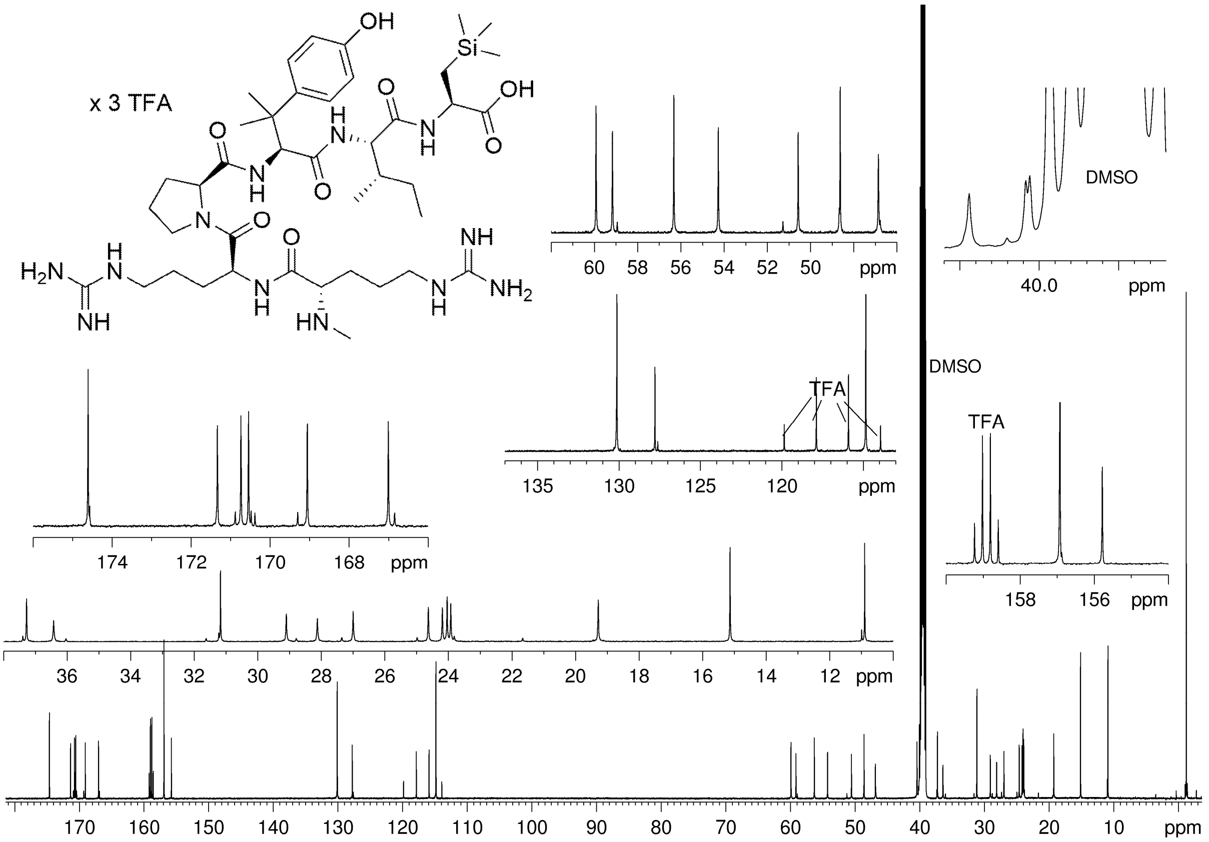

^{13}C-NMR spectrum (150 MHz, DMSO-d$_6$) of compound **16a**

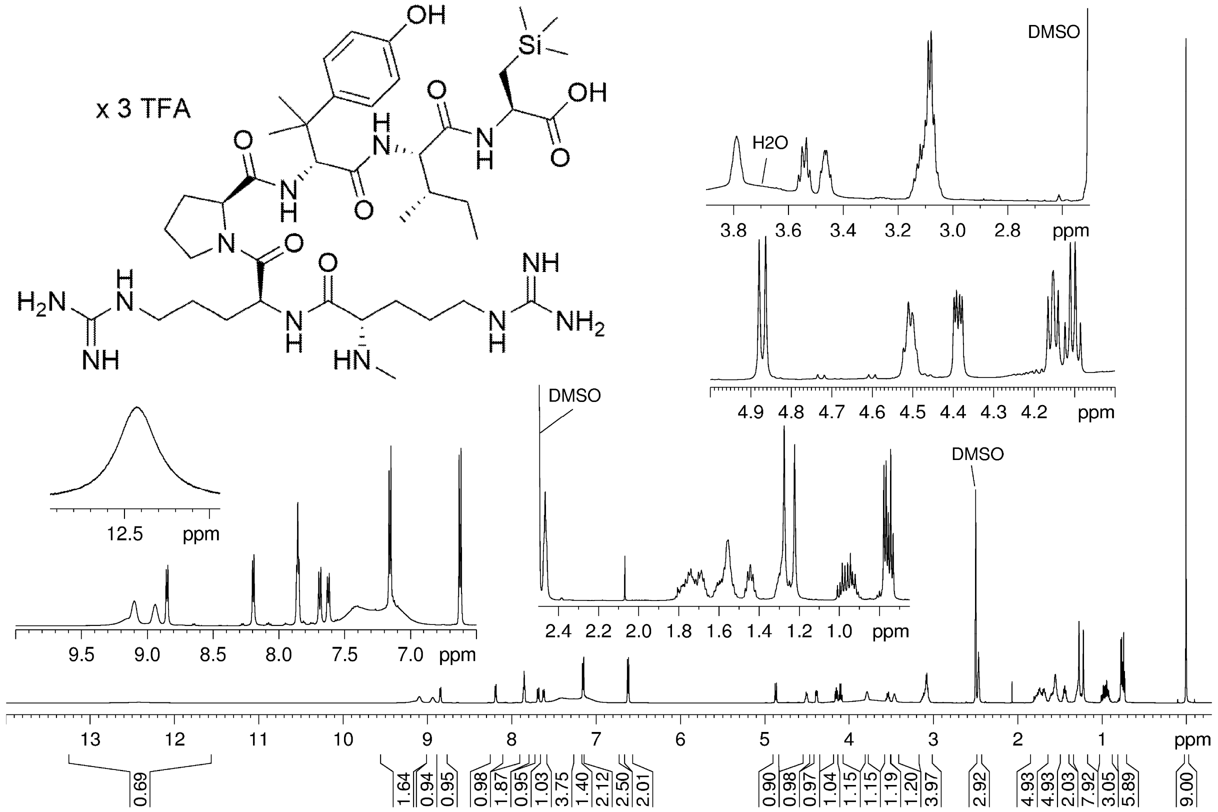

^{1}H-NMR spectrum (600 MHz, DMSO-d$_6$) of compound **16b**

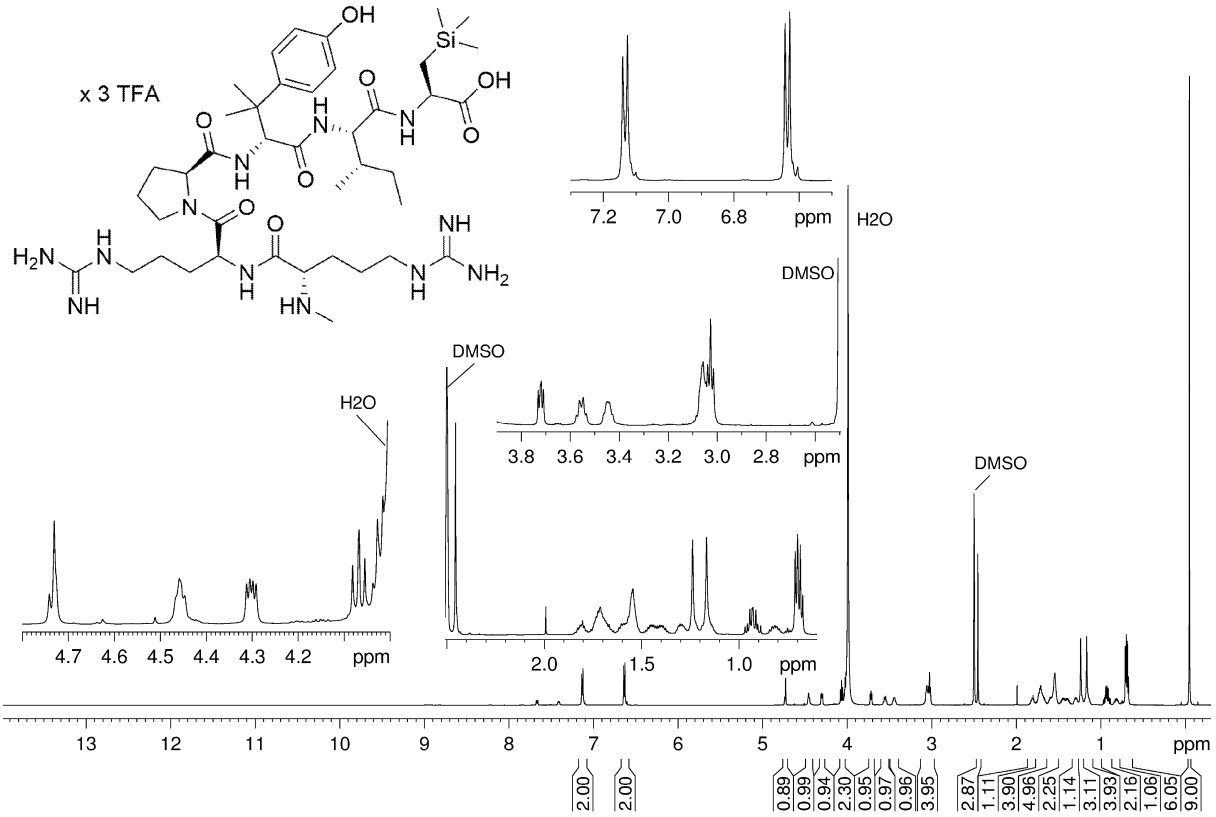

^{1}H-NMR spectrum (600 MHz, DMSO-d$_6$/D$_2$O 4:1 v/v) of compound **16b**

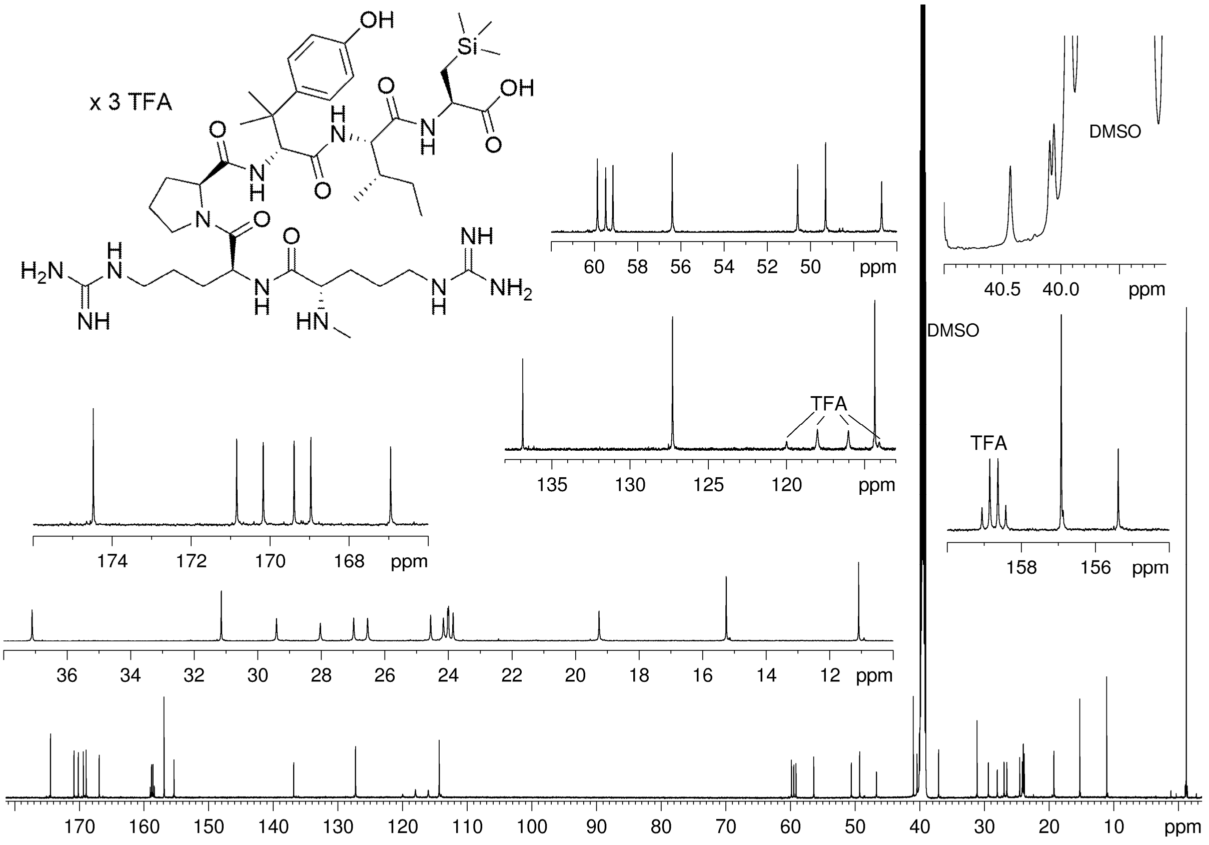

^{13}C-NMR spectrum (150 MHz, DMSO-d$_6$) of compound **16b**

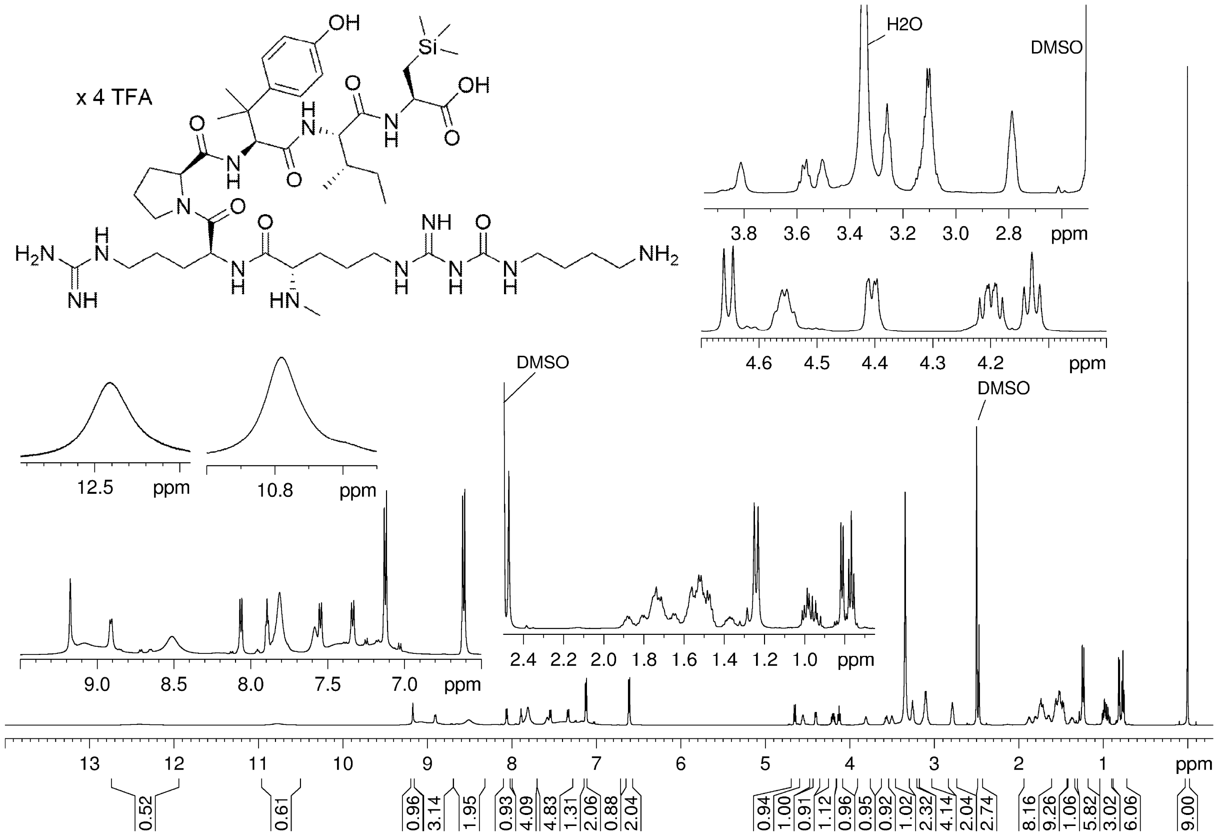

^{1}H-NMR spectrum (600 MHz, DMSO-d_6) of compound **17a**

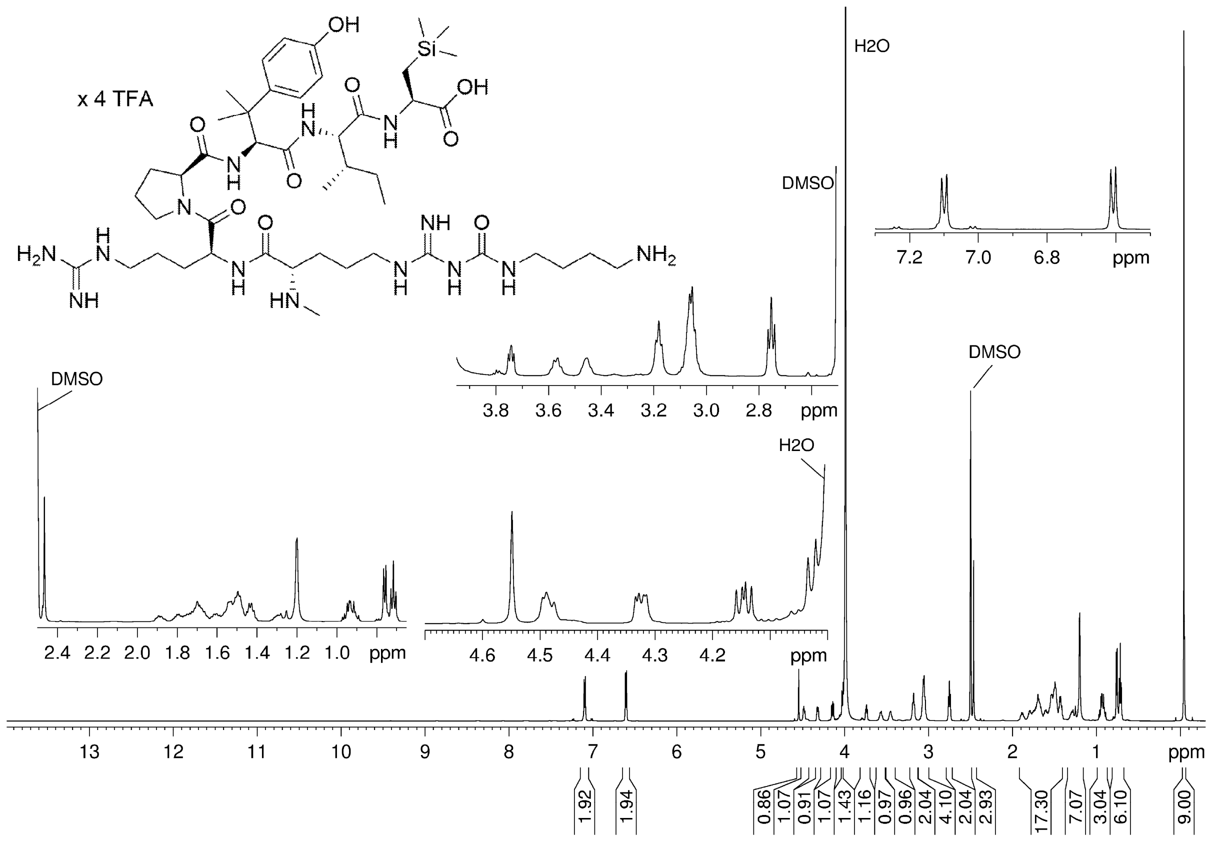

^{1}H-NMR spectrum (600 MHz, DMSO-d_6/D_2O 4:1 v/v) of compound **17a**

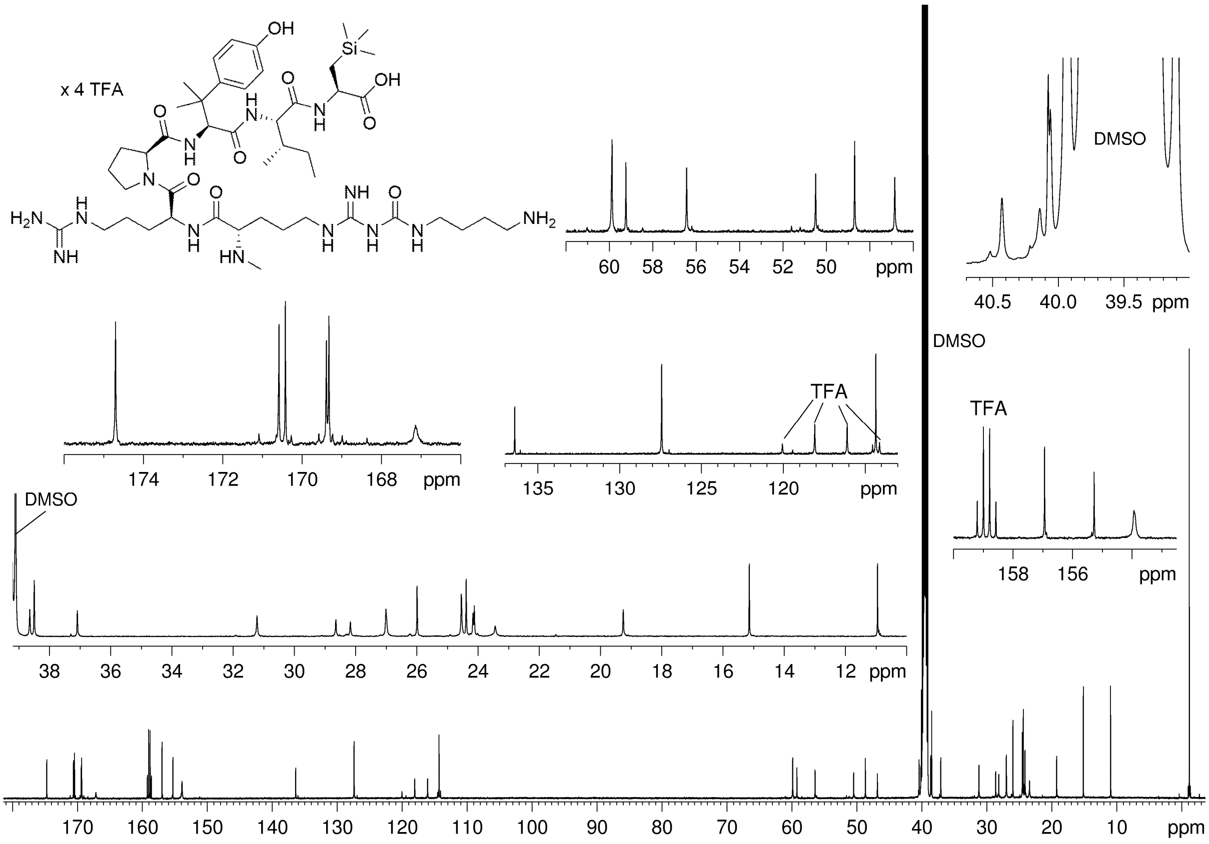

^{13}C-NMR spectrum (150 MHz, DMSO-d$_6$) of compound **17a**

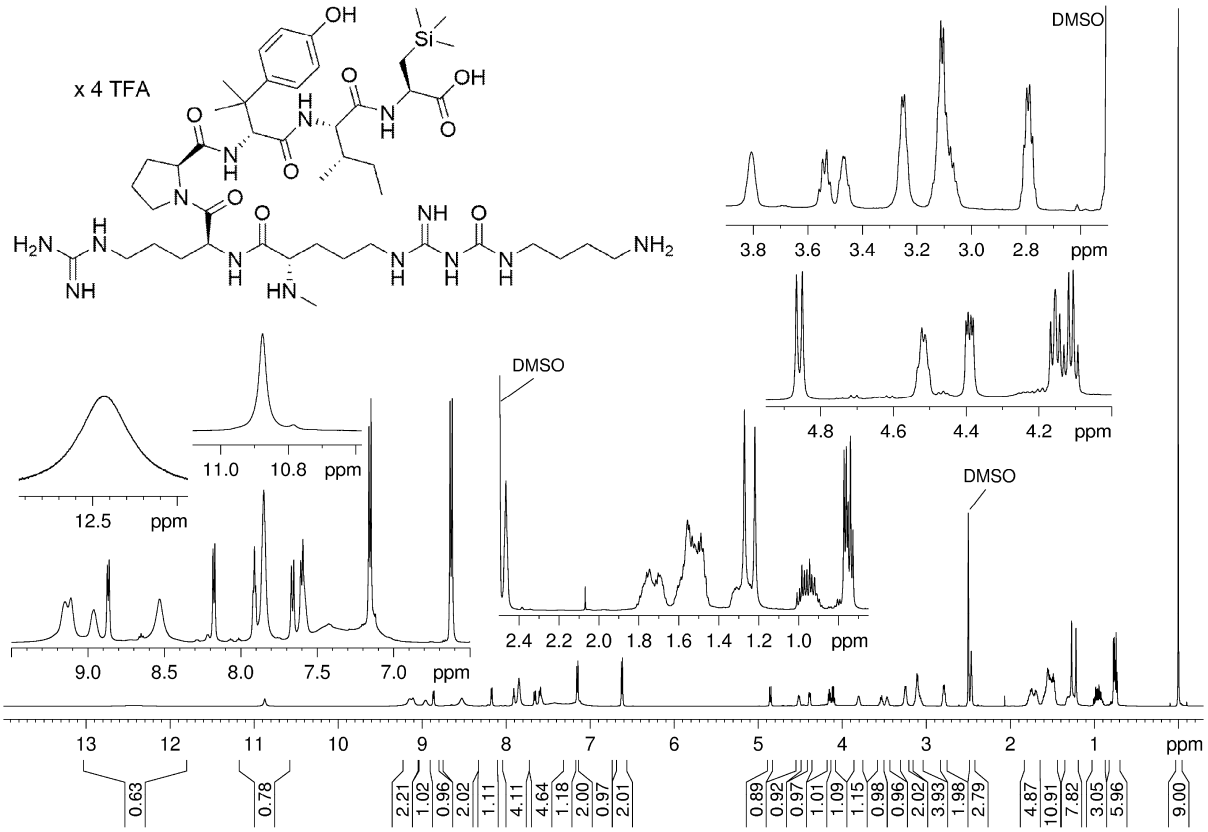

^{1}H-NMR spectrum (600 MHz, DMSO-d$_6$) of compound **17b**

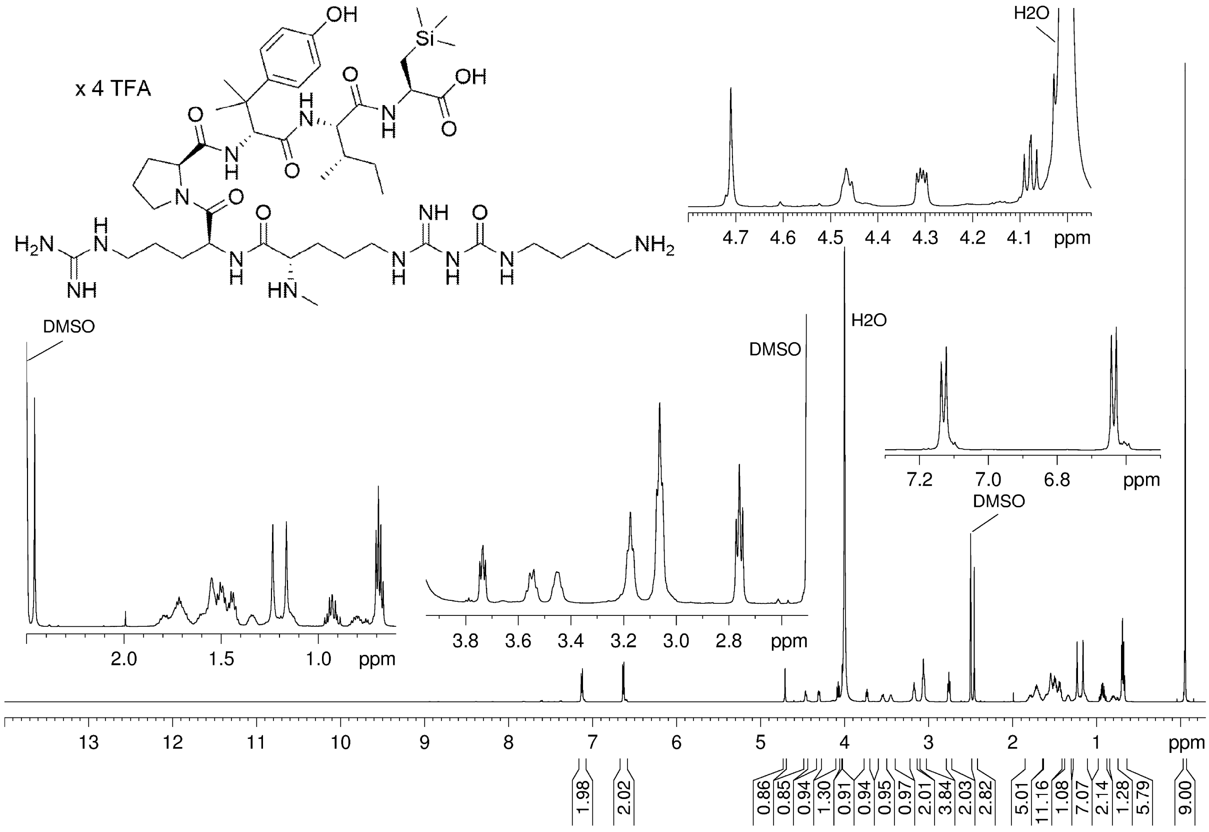

^{1}H-NMR spectrum (600 MHz, DMSO-d$_6$/D$_2$O 4:1 v/v) of compound **17b**

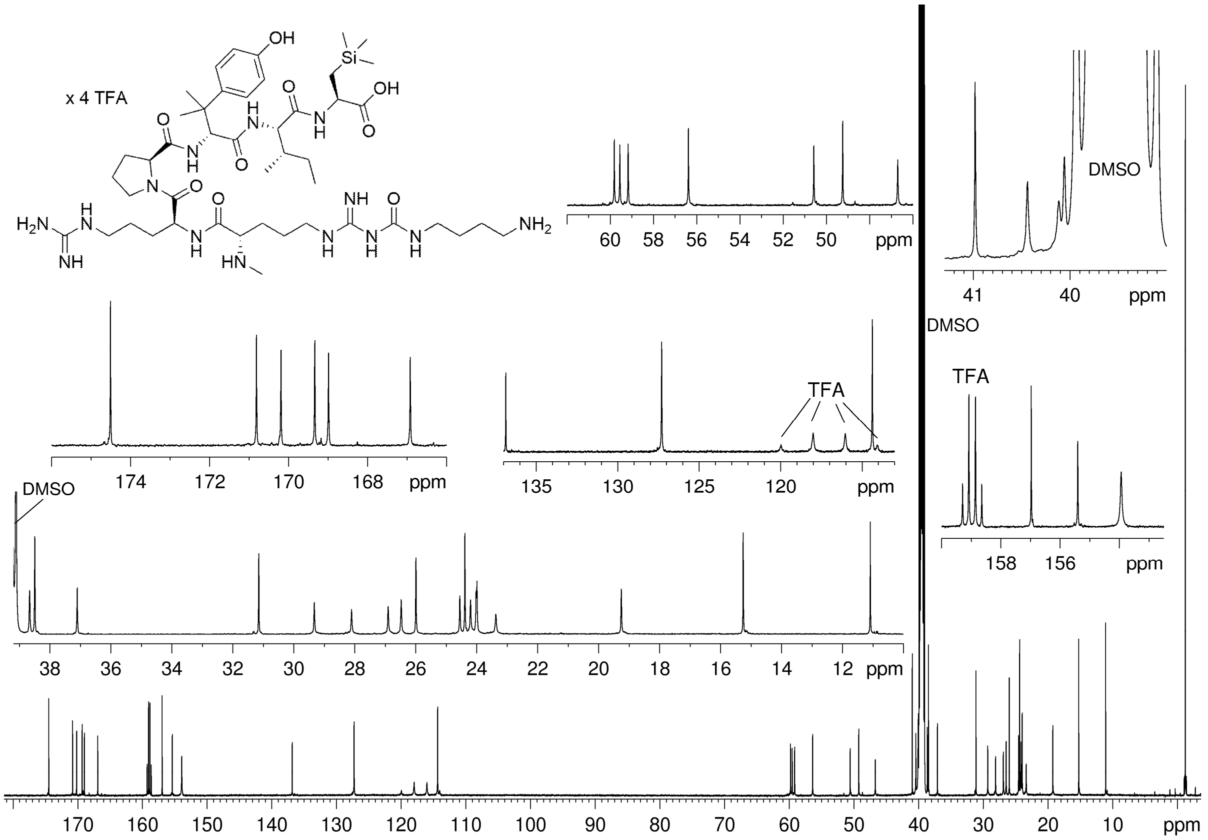

^{13}C-NMR spectrum (150 MHz, DMSO-d$_6$) of compound **17b**

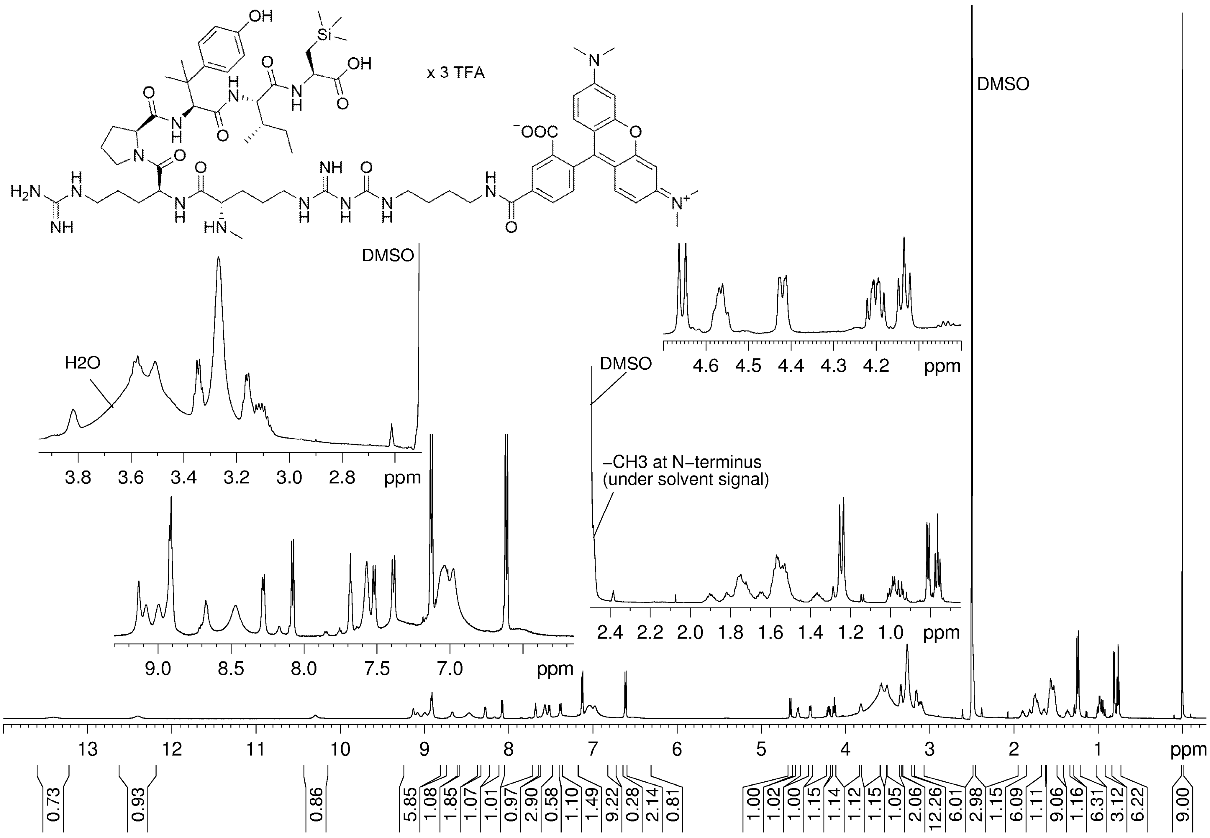

^{1}H-NMR spectrum (600 MHz, DMSO-d_6) of compound **19**

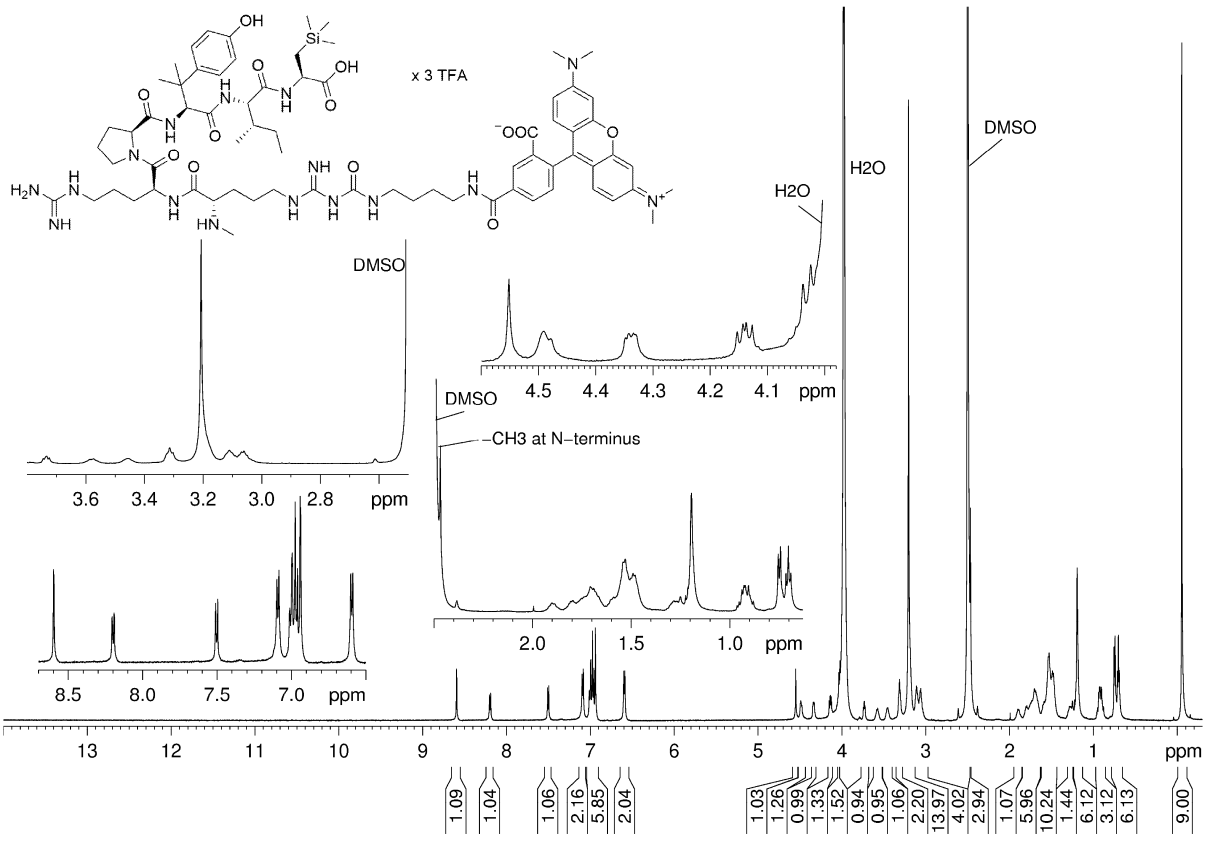

^{1}H-NMR spectrum (600 MHz, DMSO-d_6/D_2O 4:1 v/v) of compound **19**

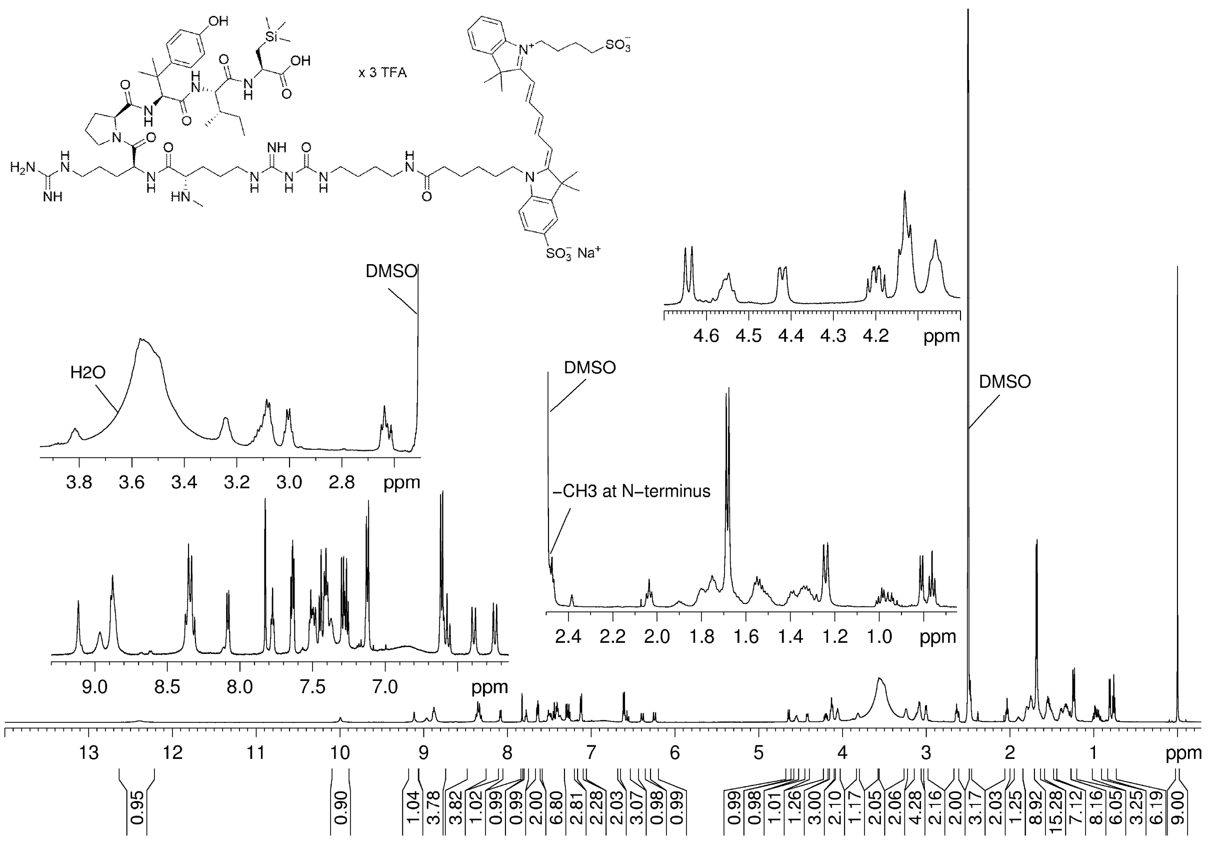

^{1}H-NMR spectrum (600 MHz, DMSO-d$_6$) of compound **21**

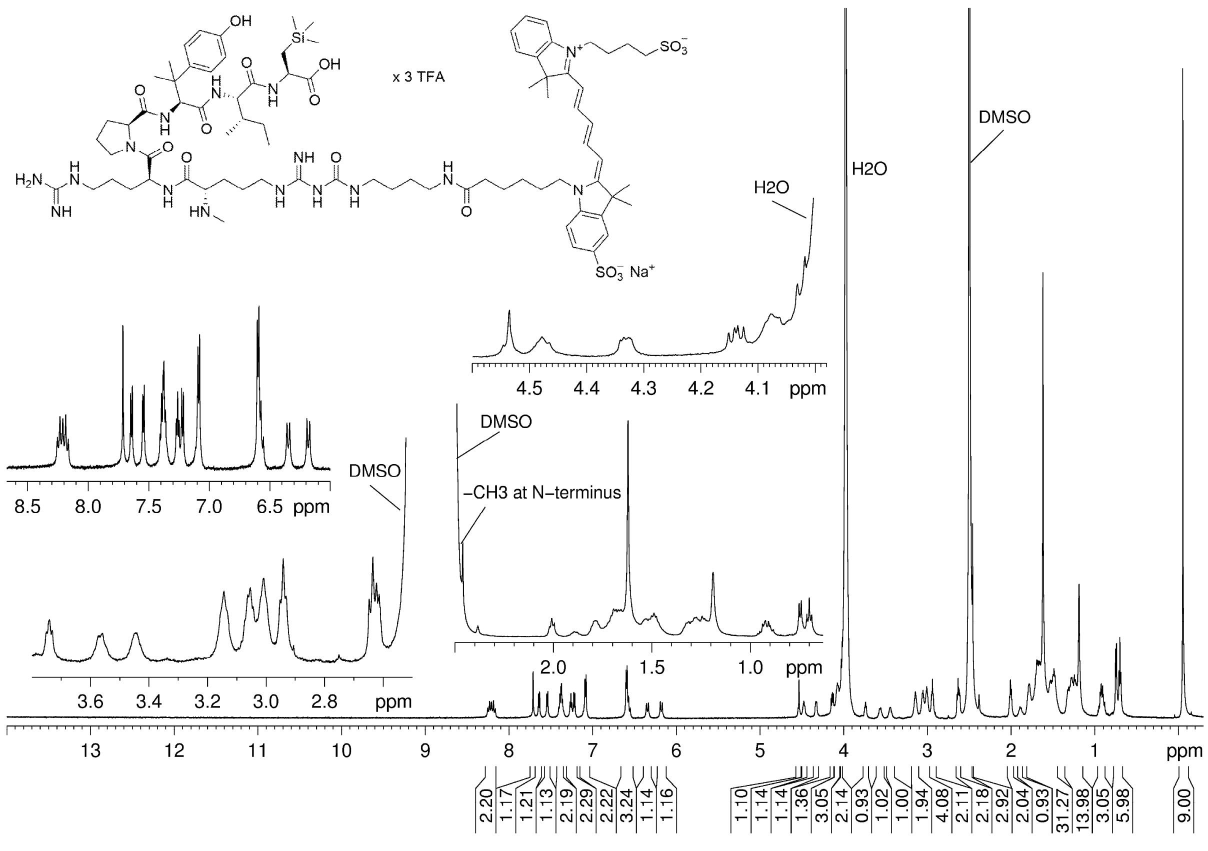

^{1}H-NMR spectrum (600 MHz, DMSO-d$_6$/D$_2$O 4:1 v/v) of compound **21**

8. References

(1) René, A., Vanthuyne, N., Martinez, J., Cavelier, F. (L)-(Trimethylsilyl)alanine synthesis exploiting hydroxypinanone-induced diastereoselective alkylation. *Amino acids* **2013**, *45*, 301-307.

(2) Schindler, L.; Moosbauer, J.; Schmidt, D.; Spruss, T.; Grätz, L.; Ludeke, S.; Hofheinz, F.; Meister, S.; Echtenacher, B.; Bernhardt, G.; Pietzsch, J.; Hellwig, D.; Keller, M. Development of a neurotensin-derived ^{68}Ga-labeled PET ligand with high in vivo stability for imaging of NTS_1 receptor-expressing tumors. *Cancers* **2022**, *14*, 4922.

(3) Keller, M., Kuhn, K. K., Einsiedel, J., Hübner, H., Biselli, S., Mollereau, C., Wifling, D., Svobodovà, J., Bernhardt, G., Cabrele, C., Vanderheyden, P. M. L., Gmeiner, P., Buschauer, A. Mimicking of arginine by functionalized N^{ω}-carbamoylated arginine as a new broadly applicable approach to labeled bioactive peptides: High affinity angiotensin, neuropeptide Y, neuropeptide FF, and neurotensin receptor ligands as examples. *J. Med. Chem.* **2016**, *59*, 1925-1945.

(4) Schneider, C. A., Rasband, W. S:, Eliceiri, K. W. NIH Image to ImageJ: 25 years of image analysis. *Nat. Methods* **2012**, *9*, 671-675.
